# Disassociation of Vitamin D’s Calcemic Activity and Non-calcemic Genomic Activity and Individual Responsiveness: A Randomized Controlled Double-Blind Clinical Trial

**DOI:** 10.1038/s41598-019-53864-1

**Published:** 2019-11-27

**Authors:** Arash Shirvani, Tyler Arek Kalajian, Anjeli Song, Michael F. Holick

**Affiliations:** Boston University School of Medicine, Boston Medical Campus, Section Endocrinology, Diabetes, Nutrition and Weight Management, Department of Medicine, Vitamin D, Skin, and Bone Research Laboratory, Boston, MA 02118 USA

**Keywords:** Gene expression, Endocrinology

## Abstract

The aims of this randomized controlled double-blind clinical trial were to assess the impact of vitamin D supplementation on calcium metabolism and non-calcemic broad gene expression by relating them to the individual’s responsiveness to varying doses of vitamin D_3_. Thirty healthy adults were randomized to receive 600, 4,000 or 10,000 IU/d of vitamin D_3_ for 6 months. Circulating parathyroid hormone (PTH), 25(OH)D, calcium and peripheral white blood cells broad gene expression were evaluated. We observed a dose-dependent increase in 25(OH)D concentrations, decreased PTH and no change in serum calcium. A plateau in PTH levels was achieved at 16 weeks in the 4000 and 10,000 IU/d groups. There was a dose-dependent 25(OH)D alteration in broad gene expression with 162, 320 and 1289 genes up- or down-regulated in their white blood cells, respectively. Our results clearly indicated that there is an individual’s responsiveness on broad gene expression to varying doses of vitamin D_3_. Vitamin D_3_ supplementation at 10,000 IU/d produced genomic alterations several fold higher than 4,000 IU/d even without further changes in PTH levels. Our findings may help explain why there are some inconsistency in the results of different vitamin D’s clinical trials.

## Introduction

Vitamin D sufficiency, insufficiency and deficiency are defined as serum levels of 25-hydroxyvitamin D [25(OH)D] as 30–100 ng/mL (75–250 nmol/L), 21–29 ng/mL (51–74 nmol/L) and <20 ng/mL (<50 nmol/L) respectively by the Endocrine Society’s Practice Guidelines on Vitamin D^1–3^. The influence of vitamin D in maintaining skeletal balance is clearly explained^1–3^. In addition to its important role in calcium balance, a long list of acute and chronic medical conditions including cardiovascular disease^4^, autoimmune diseases^5^, neurocognitive dysfunction^6^, type 2 diabetes^7^, infectious diseases and deadly cancers have been connected to vitamin D deficiency^1,2,8^. Debate continues on what an “optimal” serum concentration of 25(OH)D is and how much supplementation is required to achieve it. The Institute of Medicine (IOM) recommended that 600 IU/d as adequate to sustain a serum concentration of 25(OH)D above 20 ng/mL for most children and adults and prevent negative outcomes for their bone health^1,3^.

Although the IOM’s upper limit of vitamin D for adults is 4000 IU/d, the IOM and the Endocrine Society recognize that up to 10,000 IU/d is completely safe in healthy adults for up to 5 months^1,3,9^. Ekwaru *et al*.^10^ reported that receiving up to 20,000 IU/d for at least one year did not achieve serum concentration of 25(OH)D above 100 ng/mL in adults^10^. Many reference laboratories^11^ as well as the Endocrine Society suggested this serum concentration of 25(OH)D (100 ng/mL) as the upper limit of normal. The most epidemiological studies indicated that serum 25(OH)D > 30 ng/mL were related with immune function improvement and reduced risk of many chronic illnesses^8,12^. However studies evaluating supplementation with vitamin D up to 2000 IU/d with an average of 5 years of follow-up did not find benefit for reducing risk for cardiovascular disease or cancer^13^. In the Vitamin D and Omega-3 Trial (VITAL)^13^, a large primary-prevention trial, supplementation with vitamin D_3_ (at a dose of 2,000 IU/d) did not show any significant association with lower occurrence of cardiovascular events or invasive cancer than placebo^13^. Despite that vitamin D supplementation did not associate with a lower occurrence of total deaths from cancer than placebo, yet found that mortality due to cancer was significantly lower with vitamin D supplementation^13^. Patients’ quality of life in hospital was improved after supplementation with vitamin D_3_ at 10,000 IU/d over 6 months and improved hormonal factors b-type natriuretic peptide (BNP) and PTH as well as inflammation^14^. These findings suggest possible health benefits of doses of vitamin D above the RDA.

However, the influence of vitamin D supplementation on genome-wide expression remains largely unexplored *in vivo,* particularly with respect to dose. We previously evaluated the influence of vitamin D supplementation on genome-wide expression and found differential regulation of about 300 genes because of supplementation with vitamin D_3_^8^. This randomized controlled double-blinded study included healthy young adults who received either 400 IU/d of vitamin D_3_ (the study occurred in 2010 before the IOM released its new recommendation of 600 IU/d) or 2,000 IU/d for 2 months. The alteration of gene regulation in the 400 IU/d group was the same as 2,000 IU/d^8^. However, we detected a greater change in gene regulation in the subjects of the 2,000 IU/d group in comparison the 400 IU/d group^8^. Pathway and functional analysis of the target genes indicated that vitamin D supplementation may be connected with some pathways related to epigenetic modification, immune system and response to stress^8^. Many of these activities of vitamin D have been hypothesized to play roles in disease susceptibility including autoimmune disease and cancer^8,12^. The current clinical trial was designed to evaluate the responsiveness of different doses of vitamin D supplementation (600 IU/d, 4,000 IU/d and 10,000 IU/d) on serum calcium, PTH and broad gene expression in white blood cells. Our previous study supported the theory that higher doses of vitamin D could have important biologic functions at least on the immune cells^8^. The hypothesis that was tested was that as serum concentration of 25(OH)D increased as a result of vitamin D supplementation that there would be a significant decline in PTH levels. Furthermore, we expected that there would be continued alterations in gene expression when the dose was increased to 10,000 IUs daily whereas the PTH levels would plateau at a dose of 4000 IUs daily.

## Results

### Baseline characteristics

**Figure 1:**
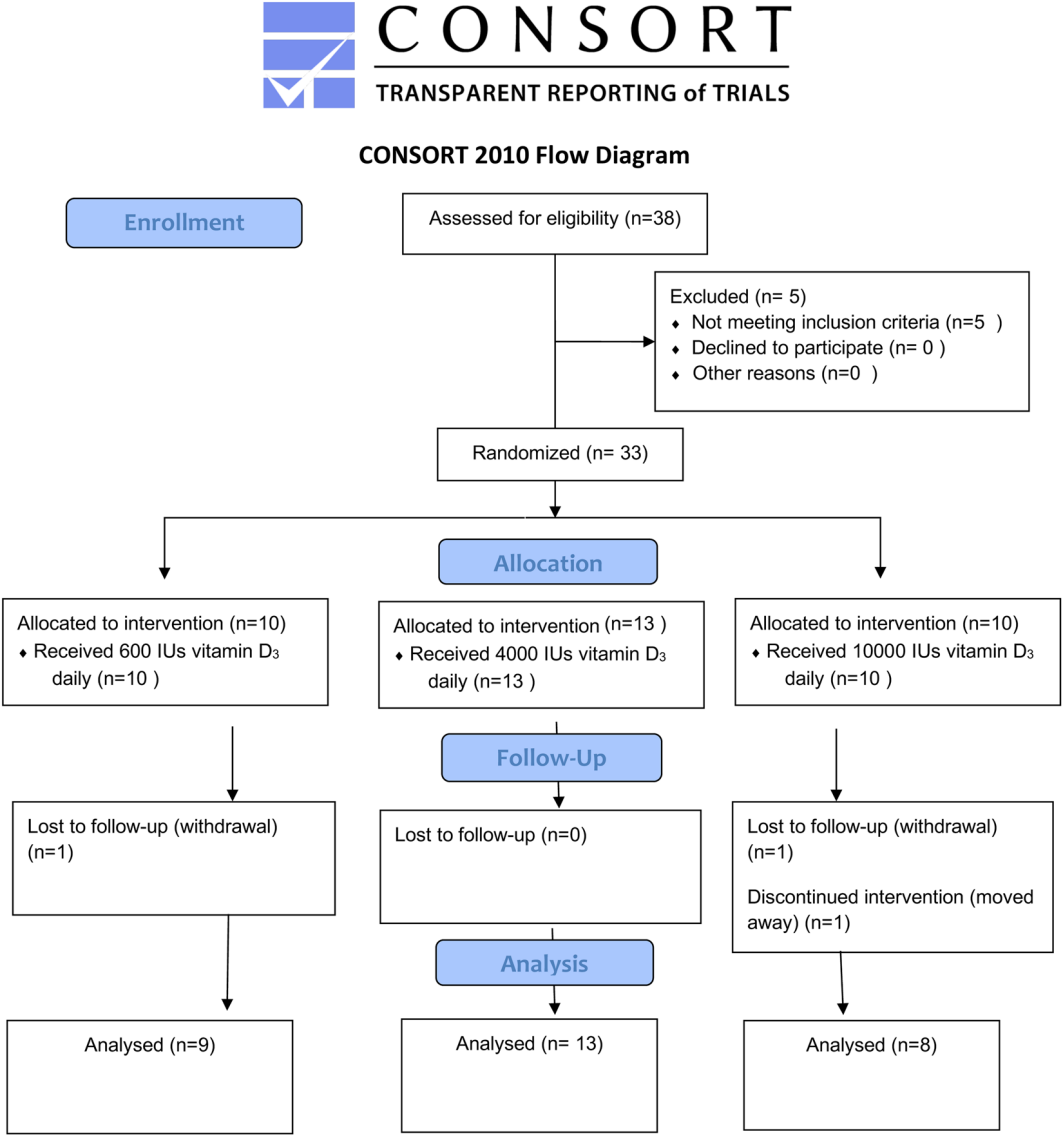
Depicts the CONSORT flow diagram, the adverse events as well as the reasons for withdrawal. With 3 participants withdrawing from the study, the overall dropout rate was 10% with one subject resigning from the arm 1 (to receive 600 IUs of vitamin D_3_), and 2 subjects from the test arm 3(to receive 600 IUs of vitamin D_3_).

Participants were recruited between November 2016 and March 2018. The baseline examination was performed in November 2016 and October 2017. After consideration criteria for inclusion and exclusion, a total of 33 adult subjects recruited. Thirty participants (90%) completed the 6-month trial. The CONSORT flow diagram is shown in Fig 1.

Baseline serum concentrations of calcium, PTH and total 25(OH)D are shown in Table 1.

**Table 1:** Baseline characteristics off the participants by dose group and their total serum 25(OH)D levels before and after 24 weeks vitamin D_3_ supplementation.

CHARACTERISTICS	VITAMIN D_3_ DOSE ASSIGNMENT (IU/D))		
	600 IU/d (N = 9)	4000 IU/d (N = 13)	10,000 IU/D (n = 8)
Sex (Female)	6	8	5
Race (Non-White)	6	5	4
Age (years)	26.3 ± 2	25.3 ± 2.1	26.1 ± 2
25(OH)D(ng/mL) before supplementation	17.1 ± 5.9	22.5 ± 5.7	17.8 ± 3.3
25(OH)D (ng/mL) after supplementation	24.3 ± 4.1	40.8 ± 3.8	78.6 ± 13.5
PTH (pg/mL) before supplementation	26.9 ± 8.9	34.6 ± 11.9	36 ± 12.5
PTH (pg/mL) after supplementation	30.4 ± 14.5	27.7 ± 8.3	24.9 ± 11.9
Calcium (mg/dL) before supplementation	9.4 ± 0.2	9.6 ± 0.1	9.6 ± 0.3
Calcium (mg/dL) after supplementation	9.4 ± 0.4	9.6 ± 0.4	9.7 ± 0.3

### Influence of the supplementation with vitamin D_3_ on circulation levels of PTH and 25(OH)D

**Figure 2:**
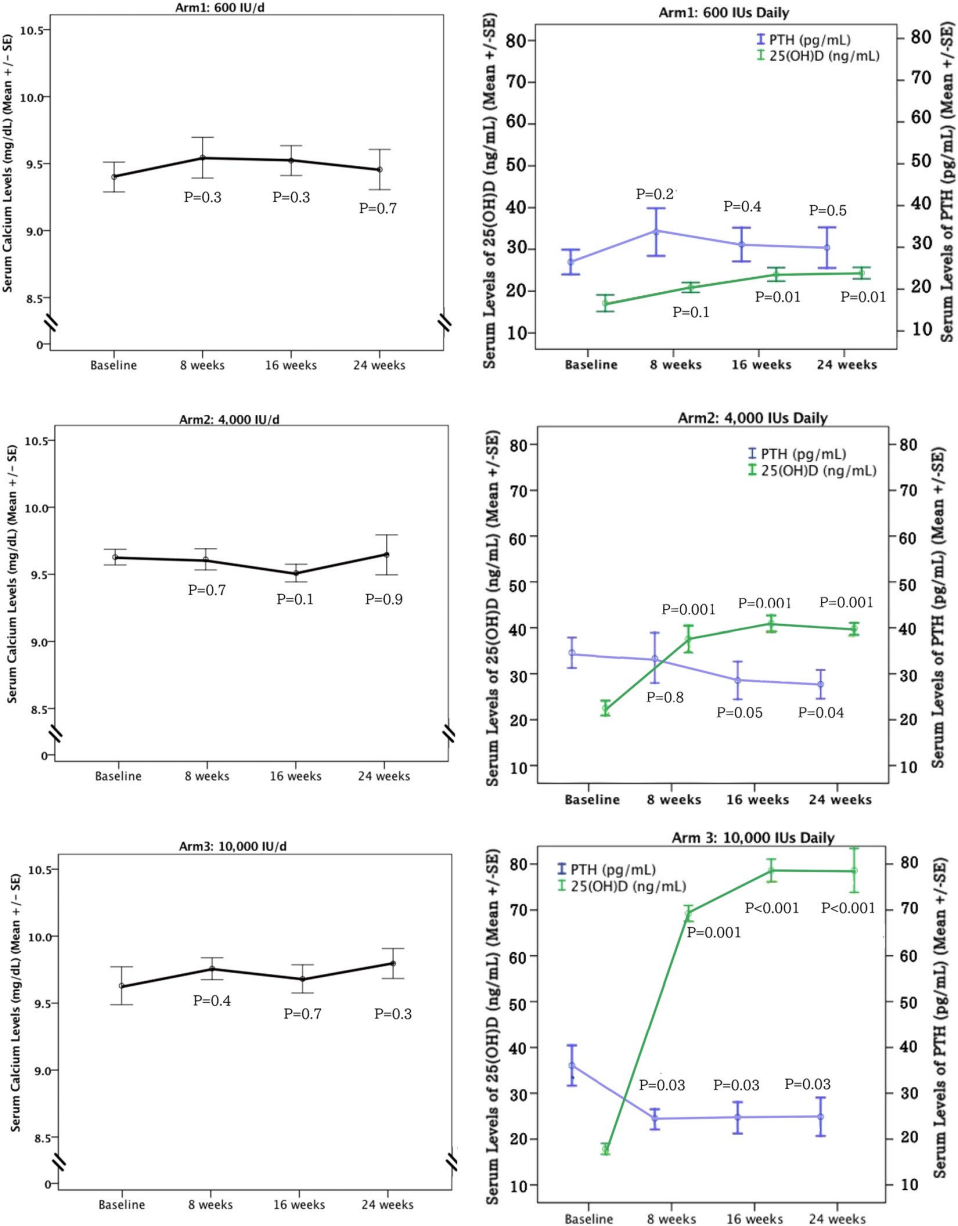
Total concentration of calcium, PTH and 25(OH)D during vitamin D_3_ supplementation at baseline and every eight weeks for 24 weeks. Total 25(OH)D, PTH and calcium levels during vitamin D_3_ supplementation are evaluated at baseline and every eight weeks for 24 weeks. The p-value in the figure represents the difference between in each time point with the baseline.

The average increase in 25(OH)D over 24 weeks was 7 ng/mL (18 nmol/L), 18 ng/mL (45 nmol/L) and 61 ng/mL (153 nmol/L) for 600 IU/d, 4000 IU/d and 10,000 IU/d, respectively (Table 1). Figure 2 depicts serum concentrations of 25(OH)D and PTH by dose group.

Mean serum concentrations of 25(OH)D at baseline were 17.1 ± 5.9 ng/mL (43.8 ± 14.8 nmol/L). After 24 weeks on 600 IU/d serum 25(OH)D significantly (p = 0.01) increased to 24.3 ± 4.1 ng/mL (60.8 ± 10.3 nmol/L) at end-of-study. The 600 IU/d dose was sufficient to correct vitamin D deficiency (25(OH)D above 20 ng/ml) in 71% of subjects but did not result in sufficiency (25(OH)D above 30 ng/ml) and 86% of subjects were still remain insufficient. There was no significant change in serum concentrations of PTH or calcium in the 600 IU/d group (p > 0.05).

The mean serum 25(OH)D level that was achieved for the groups that ingested 4,000 and 10,000 IU/d vitamin D_3_ daily for 24 weeks was 40.8 ± 3.8 ng/mL (102 ± 9.5 nmol/L) and 78.6 ± 13.5 ng/mL (196.5 ± 33.8 nmol/L), respectively. All participants in the 4000 and 10,000 IU/d groups achieved 25(OH)D levels >30 ng/ml (>75 nmol/L). There was no significant change in serum calcium for either group (Table 1). Significant decreases in PTH levels of 17.5% and 33.3% at 16 weeks were found for the 4000 and 10,000 IU/d group, respectively (p = 0.04). PTH levels remained at that level for the remaining 8 weeks (Fig. 2). There were no significant differences between men and women with respect to changes in serum concentration of calcium, 25(OH)D or PTH in response to supplementation with vitamin D_3_.

### Influence of supplementation with vitamin D_3_ on genome wide expression in buffy coat

Differential expression analysis was performed using fold change >1.5 to identify a total of 162, 320 and 1289 differentially expressed genes (DEGs) that were influenced after vitamin D_3_ supplementation with dose of 600, 4,000 and 10,000 IU/d, respectively. The recognized target genes are shown in Supplementary Tables 1 and 2.

The microarray results of gene expression alterations were confirmed by real-time PCR for 7 genes including *HIST1H2BB, TLR1, CYP24A1, LRRN3, RPL3, OCLM* and *HBE1* (Table 2).

**Table 2:** Quantitative Real Time PCR (qRT-PCR) Validation of Microarray Data.

Official Gene Symbol	Vitamin D3 Supplementation Arms					
	600 IU/d (N = 9)		4000 IU/d (N = 13)		10,000 IU/d (n = 8)	
	Fold Change		Fold Change		Fold Change	
	Microarray	RT-PCR	Microarray	RT-PCR	Microarray	RT-PCR
*HIST1H2BB*	1.5	0.9	1.5	1.4	1.9	1.3
*TLR1*	−1.1	−1	−1.3	−0.9	−1.9	−2.7
*CYP24A1*	1.0	2.6	1.1	1.5	1.1	1.6
*LRRN3*	−1.2	−0.8	−1.3	−0.9	−2.2	−2.1
*RPL3*	1.0	1	1.1	0.6	1.2	1.1
*OCLM*	1.2	1.4	1.1	2.8	1.0	0.6
*HBE1*	1.2	1.4	1.1	0.7	1.0	1.1

An evaluation of genome-wide gene expression of genes being influenced by vitamin D_3_ supplementation revealed a greater change in the gene expression for the subjects who received 10,000 IU/d compared to the other two groups. There was a dose-dependent influence on the total number of differentially expressed genes (Table 3).

**Table 3:** The influence of varying doses of vitamin D supplementation on broad gene expression.

Vitamin D_3_ supplementation	Upregulated genes	Down regulated genes	Total	Fold change (range)	Fold change (average)
600 IU/day	86	76	162	1.5–10	1.7
4000 IU/day	188	132	320	1.5–6	1.7
10,000 IU/day	800	489	1289	1.5–23	1.8

Whereas 162 (86 up-regulated, 76 down-regulated) genes in the peripheral white blood cells were influenced the adults who took 600 IU/d for 6 months, there was 2- and 8-fold increase in the number of genes that were influenced in the groups that received 4000 IU/d and 10,000 IU/d, respectively (Table 3).

**Figure 3:**
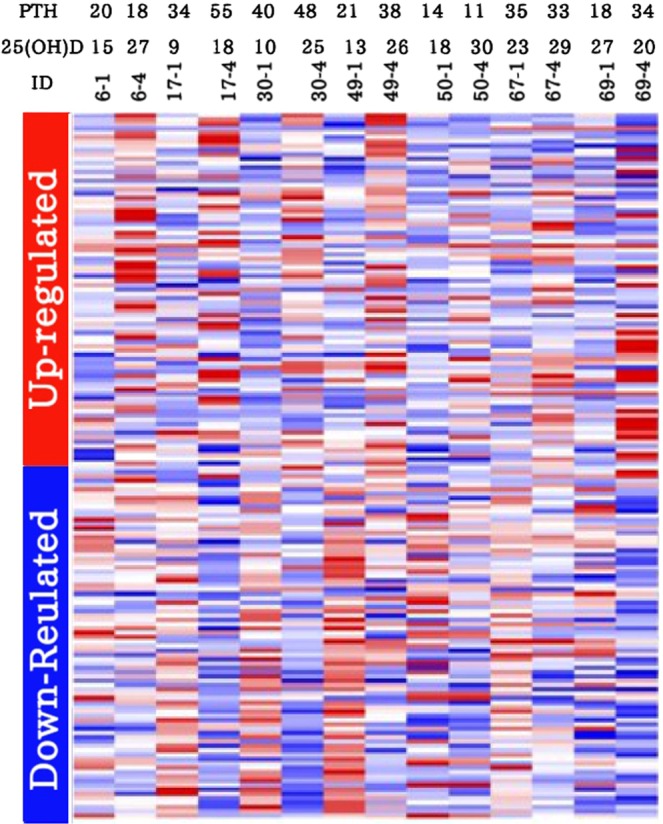
Gene expression alterations in response to 600 IU/d vitamin D_3_ supplementation. The gene-expression alterations are demonstrated by different colors. Upregulation and downregulation of gene expression after 6-months of vitamin D_3_ supplementation are shown by red and blue respectively. Trends in gene expression are seen by a range of colors from light blue to dark red. The red, white and blue represents high, average and low gene expression respectively.

We compared gene expression between the 3 groups and related them to changes in serum concentrations of PTH and 25(OH)D. Broad gene expression analysis revealed that all groups who received vitamin D_3_ supplementation had a significant number of genes that were differentially expressed. The subjects in the 600 IU/d group did not have any significant change in their serum levels of PTH but demonstrated a change in 162 genes after 6 months of supplementation (Fig. 3).

**Figure 4:**
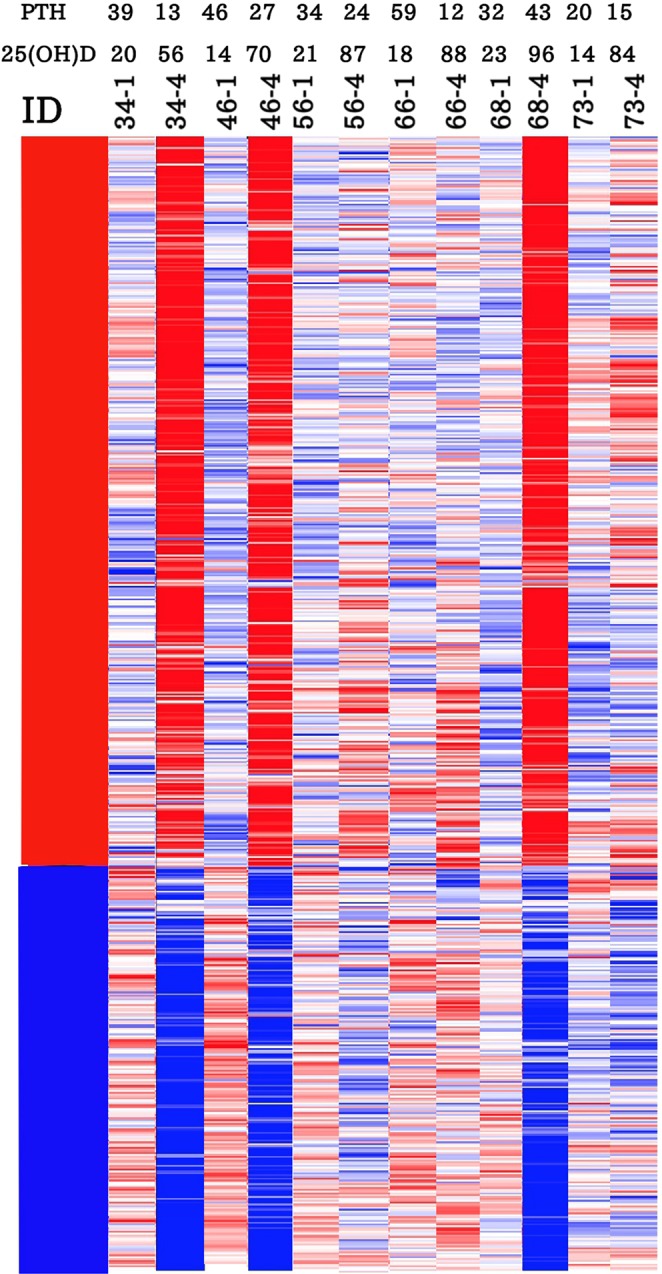
Gene expression alterations in response to 10,000 IU/d of vitamin D_3_ supplementation. The gene-expression alterations are demonstrated by different colors. Upregulation and downregulation of gene expression after 6-months of vitamin D_3_ supplementation are shown by red and blue respectively. Trends in gene expression are seen by a range of colors from light blue to dark red. The red, white and blue represents high, average and low gene expression respectively.

**Figure 5:**
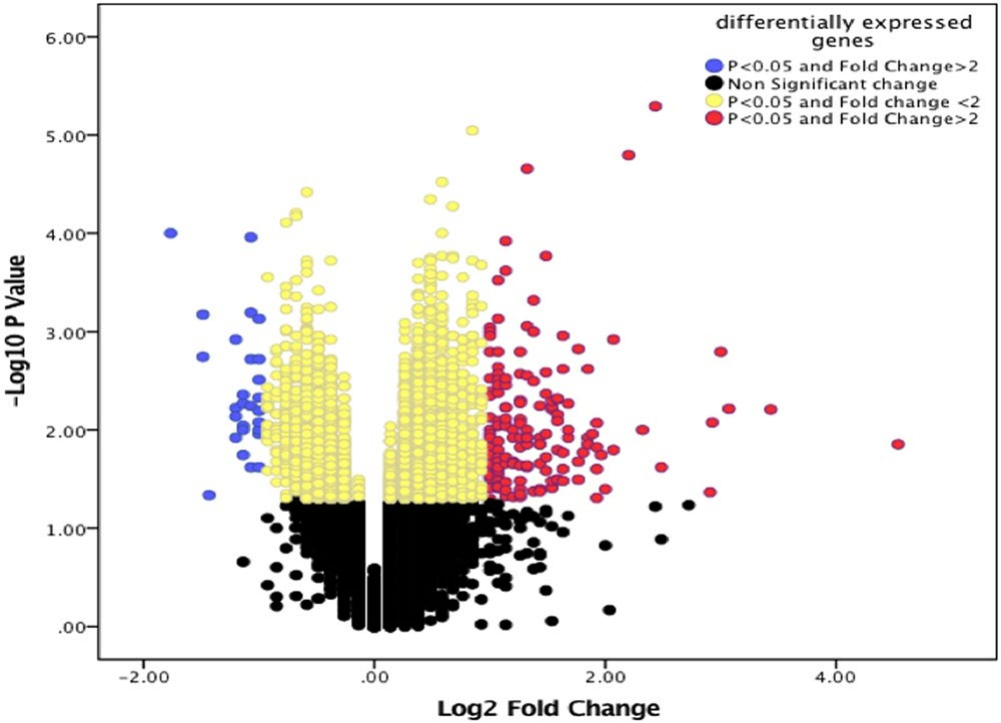
Volcano plot of target genes in the10,000 IU/d group. Log2 fold change was plotted against the p-value (−log base 10). This statistical analysis showed that 1289 genes were differentially expressed after vitamin D_3_ supplementation. Among these DEGs, 800 genes were upregulated and 489 genes were downregulated after 6 months supplementation with vitamin D_3_. Volcano plot of differentially expressed genes (DEGs) between, before and after 24 wks vitamin D_3_ supplementation (10,000 IUs/d). Log2 fold change was plotted against the p-value (−log base 10). Differential expression analysis using a p < 0.05 cutoff identified 1289 DEGs after vitamin D_3_ supplementation. Among these DEGs, 800 genes were upregulated and 489 genes were downregulated after vitamin D_3_ supplementation.

A similar pattern of variable genomic response to vitamin D_3_ supplementation was detected in the 2 groups treated with higher doses of vitamin D_3_ supplements (4,000 IU/d and 10,000 IU/d). The gene expression alterations in the group of 4,000 IU/d is shown in Supplementary Fig. 1. A similar observation was made for those subjects in the 10,000 IU/d group (Fig. 4). The statistical analysis showed that 1,289 genes were differentially expressed after vitamin D_3_ supplementation. Among these DEGs, 800 genes were upregulated and 489 genes were downregulated after vitamin D_3_ supplementation (Fig. 5).

### Variable pattern of genomic wide expression in response to supplementation with Vitamin D_3_

We compared gene expression between dose groups and related this data to changes in circulating levels of 25(OH)D and PTH to provide a clearer understanding of the biologic responsiveness to different doses of vitamin D_3_. The pattern of gene expression in response to vitamin D_3_ supplementation showed an inter-individual variation. Approximately 30% of the adults who received different doses of vitamin D_3_ supplement (600, 4000 or 10000 IU/d) for 6 months and raised serum 25(OH)D levels to the same degree as the other 70% demonstrated much less of a genomic response, despite similar increases in 25(OH)D. This variable pattern of expression is shown in Figs. 3 and 4, which displays some subjects with a very strong genomic response to vitamin D_3_ supplementation when compared to others with a weak response. Although after receiving vitamin D_3_ the broad gene expression significantly was changed in all subjects, the fold change of gene expression and the number of the differently expressed gene were different between these two groups of subjects with very strong genomic response comparing weak genomic response to vitamin D_3_.

Figure 3 depicts gene expression changes in subjects on 600 IU/d, and demonstrated that five subjects (ID# 6, 17, 30, 49 and 69) had a very strong genomic response to vitamin D_3_ supplementation when compared to two subjects (ID: 50 and 67) with a weak response. Figure 4 depicts gene expression changes in subjects on 10,000 IU/d, and demonstrated that three subjects (ID: 34, 46 and 68) had a very strong genomic response to vitamin D_3_ supplementation with many genes being up or down regulated, in comparison with one subject (ID: 73) with a moderate response and two subjects (ID: 56 and 66) with a weak response. Subject 34 had a baseline 25(OH)D of 20 ng/mL (50 nmol/L) which increased to 56 ng/mL (140 nmol/L). This subject had a robust gene expression in response when comparing this subject’s heat map at baseline and at 6 months (Fig. 4). Subject 56 had a baseline 25(OH)D of 21 ng/mL (52 nmol/L) that was comparable to subject 34. After 6 months on 10,000 IU/d this subject achieved a higher blood level of 25(OH)D (87 ng/mL ;218 nmol/L) yet had a much more subdued genomic response (Fig. 4). Approximately one third of the 10,000 IU/d group achieved similar serum 25(OH)D and PTH levels as the other two thirds of subjects, but had much less of a genomic response (Fig. 4).

This suggests that there are other factors involved in an individual’s responsiveness to the non-calcemic actions of vitamin D beyond vitamin D dose and achieved 25(OH)D concentration.

There was no significant change in expression of genes involved in vitamin D metabolism including vitamin D receptor (*VDR*), vitamin D binding protein (*DBP or GC*) and Cytochrome P450, family 24, subfamily A, polypeptide 1 (*CYP24A1*). There was no significant change from baseline expression after vitamin D_3_ supplementation between subjects with strong or weak genomic response to vitamin D_3_. Interestingly, when we compared baseline broad gene expression between subjects with a strong or weak genomic responses, the expression of 6 genes dramatically were different. These genes include Major histocompatibility complex, class II, DR beta 5 (*HLA-DRB5*) and DR beta 1 (*HLA-DRB1*), Purinergic receptor P2Y, G-protein coupled, 14 (*P2RY14*), Ribosomal protein S4, Y-linked 1(*RPS4Y1*), small nucleolar RNA, C/D box 33 (*SNORD33*), and two domains of Killer cell immunoglobulin-like receptor, long cytoplasmic tail, 1 (*KIR2DL1*) and Early growth response 2 (*EGR2*).

### Pathway and functional analysis of the genes that are differentially expressed

**Figure 6:**
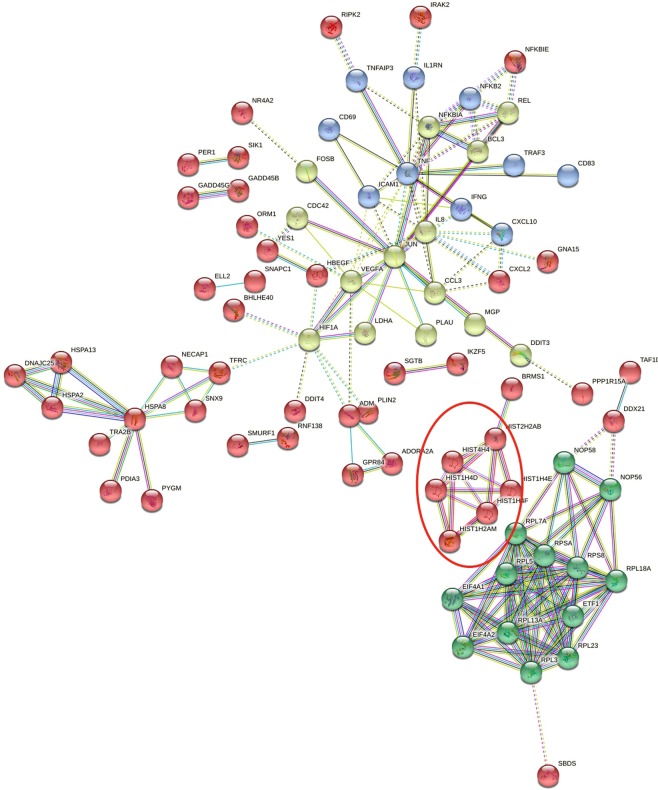
Protein-protein interaction (PPI) network was created from significantly upregulated genes after 6-months of supplementation with vitamin D_3_. Protein-protein interaction (PPI) network was constructed from differentially upregulated genes after vitamin D_3_ supplementation. We mapped differentially upregulated genes to the STRING database (the hub protein was selected according to the node degree) and screened significant interactions with a score >0.7. The key genes in these clusters were *HIST1H2B, JUN, NFKB, TNF, IL8, HSPA8, EIF4A* and *PRS*. Network nodes represent proteins. Each node represents all the proteins produced by a single protein-coding gene locus. Edges represent protein-protein associations. Associations are meant to be specific and meaningful, i.e. proteins that jointly contribute to a shared function; this does not necessarily mean they are physically binding each other. Line color indicates the type of interaction evidence. The navy, cornflower and Olympic-blue lines indicate predicted gene co-occurrence, known interactions from curated databases and protein homology respectively. The red and purple lines indicate gene fusions and known experimentally determined interactions. Also, the green and light green lines indicate predicted gene neighborhood and textmining. Finally, the black line indicates co-expression. Line thickness indicates the strength of data support and line shape indicates the predicted mode of action^17,18^. Furthermore, the network is clustered to 4 specified clusters by kmeans clustering^17,18^ of which one is related to histone modification (red circle).

The important applicable molecular pathways resulting from the alteration of gene regulation in response to 6 months of supplementation with vitamin D_3_ is shown in Fig. 6. Protein-protein interaction (PPI) network was created from the target genes^8^. We mapped upregulated genes to the STRING database^8^ and selected high confidence for interactions score. The result was significant for PPI enrichment (p-value = 9.33e^−14^). The key genes in these clusters were *HIST1H2B, JUN, NFKB, TNF, IL8, HSPA8, EIF4A* and *PRS* (Fig. 6).

These significant interactions in our molecular network indicated that these proteins might be associated to a specific pathway and biologically should be connected^8,15^. This network includes 4 clusters of which one is related to histone modification (red circle in Fig. 6). Epigenetic modification such as histone modification and chromatin regulation are necessary mechanisms to control gene expression^15^. Gene Ontology showed that these genes in this network may regulate DNA accessibility and stability of chromosomes (red circle in Fig. 6) via histone modification as well as remodel the nucleosomes^17^. This finding may explain the role of vitamin D supplementation on chromatin accessibility. Furthermore, the accessibility of chromatin and vitamin D supplementation might be considered as a factor involved in individual alteration pattern of broad gene expression. The other clusters in this network are related to signaling pathways of *NF-kappa B*, *TNF*, NOD-like receptor, T cell receptor, *mTOR*, Chemokine, *MAPK*, Toll-like receptor and pathways in cancer.

PPI network was also constructed from differentially downregulated genes. The key genes in this network are *TLR1*, *CD180* and *LRRN3* (Supplementary Fig. 2).

### Adverse events

There was no significant change in the serum calcium levels in any dose group. There was no sign of toxicity over 24 weeks of the study.

## Discussion

**Figure 7:**
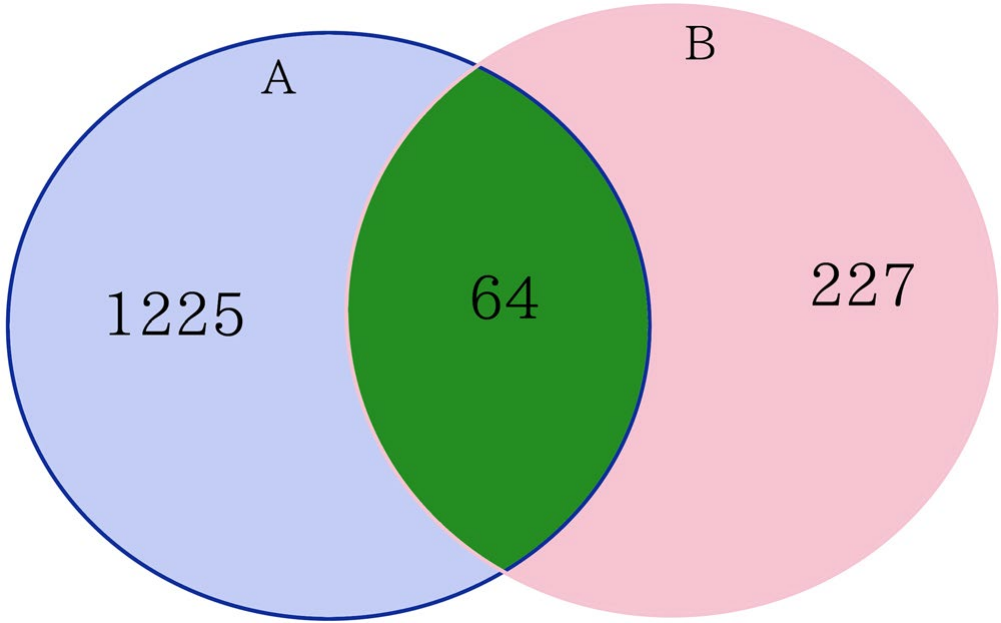
Venn diagram for comparing the number of affected genes between current study and our previous study^8^. (**A**) Differential expression analysis identified 1289 DEGs (differentially expressed genes) after 24 wks vitamin D_3_ supplementation (10,000 IUs/d). (**B**) Differential expression analysis identified 291 DEGs after 8 wks vitamin D_3_ supplementation (400 or 2,000 IUs/d). Venn diagram for comparing the number of differentially expressed genes (DEGs) between current study and our previous study^4^. (**A**) Differential expression analysis identified 1289 DEGs after 24 wks vitamin D_3_ supplementation (10,000 IUs/d). (**B**) Differential expression analysis identified 291 DEGs after 8 wks vitamin D_3_ supplementation (400 or 2,000 IUs/d).

This broad gene expression study describes the first insight into the genome wide regulation activity of different doses of vitamin D_3_ supplementation in human white blood cells. As shown in Table 3, differential expression analysis was performed using fold change >1.5 to identify a total of 162, 320 and 1,289 differentially expressed genes (DEGs) that were affected after vitamin D_3_ supplementation with doses of 600, 4,000 and 10,000 IU/d, respectively. These genes are related to epigenetic modification and immune function (Fig. 6). This observation is consistent with our previous study that showed that supplementation with vitamin D_3_ influenced the regulation of about 300 genes that were related to epigenetic modification and immune function (Fig. 7)^8^.

The effectiveness of vitamin D_3_ supplementation is connected to its ability to produce adequate substrate [25(OH)D] for its metabolism to 1,25-dihydroxyvitamin D [1,25(OH)_2_D] in the kidneys and other tissues^2,4,8^.

*In vitro* studies showed that 1,25(OH)_2_D_3_ regulated about two hundred genes that are involved in immune function, cellular proliferation and differentiation as well as angiogenesis^18–20^. Our results confirm previous findings that have indicated that VDR stimulation may affect genome-wide expression^18–23^. A human genome study showed that there are about 2,776 VDREs by the length of the human genome^22,23^.

There continues to be controversy as to whether reaching blood concentrations of 25(OH)D above 30 ng/mL would have any additional health benefits^3,12^. Our results demonstrated that PTH plateaued when 25(OH)D ≥ 30 ng/mL (75 nmol/L) and confirms previous observations that serum concentrations of PTH continued to decrease and reach a plateau when circulating levels of 25(OH)D > 30 ng/mL^24–26^. The effect of increasing vitamin D_3_ from 4,000 IU/d to 10,000 IU/d had no significant additional effect on the PTH levels (Fig. 2). However, the gene expression analysis demonstrated a dose dependent effect. Even for subjects who took 600 IU/d of vitamin D_3_ for 24 weeks, a dose that had little effect on PTH levels, this dose significantly affected the expression of more than 100 genes. In comparison, the groups who received vitamin D_3_ supplement 4,000 and 10,000 IU/d for 24 weeks had a similar effect on lowering the blood levels of PTH, but the group who received 10,000 IU/d had 4-fold greater effect on gene expression, influencing ~1,200 genes compared to the group who took 4,000 IU/d (about 300 genes). These results indicated that even a small increase in vitamin D_3_ intake of 600 IU/d for 24 weeks, a dose that did not alter the PTH levels, exerted significant genomic effects. Therefore, randomized controlled trials that include a “placebo” group receiving the RDA of 600 IU/d are confounded by unanticipated changes in gene expression. Our findings showed that there was a dissociation between the calcemic and non-calcemic biologic actions of vitamin D_3_, especially on functions involved in immune activity.

These results may help explain the disparity of conclusions regarding the studies that have evaluated the impact of supplementation with vitamin D_3_ on serum 25(OH)D improvement and clinical outcomes. An individuals response to vitamin D_3_ is related to the individual’s ability to convert vitamin D to its active metabolite and 1,25(OH)_2_D’s interaction with its receptor and response elements^8,27,28^.

Similar to our findings, two recent studies (VitDmet and VitDbol trials) indicated that there are individual differences in response to supplementation with vitamin D^23,27,28^. Based on their conclusion, this response to vitamin D is explain by epigenetic and genetic individual differences^27,28^. Our results support this conclusion that indicated in subjects with a lower genomic response to vitamin D_3_ supplementation and there was a response in ~2–5% of the genome while in more responsive subjects >5% of the genome responded to vitamin D_3_.

The most pronounced vitamin D response occurred in genes involved in genetic regulation (Fig. 5 and Supplementary Fig. 2). The major upregulated genes were *HIST1H2B, JUN, NFKB, TNF, IL8, HSPA8, EIF4A* and *PRS* and the major down regulated genes were *TLR1*, *CD180* and *LRRN3*. The upregulated genes were involved in 4 main clusters, one of which is related to histone modification (red circle in Fig. 6). Epigenetic modification, such as histone modification and chromatin regulation, are essential parts of gene regulation^16,17^. These findings are consistent with our previous study^8^ and several other studies^29,30^ that showed that vitamin D supplementation caused alteration in some genes related to epigenetic modification.

The small sample size of participants is a major limitation of the current clinical trial. In spite of this small sample size, the results of broad genomic expression are associated with an acceptable FDR. The use of microarray technology for evaluating gene expression is another limitation of our study. RNA-sequencing is more popular than microarray for evaluating broad gene expression. Nevertheless, RNA-sequencing has been associated with some limitations and biases^31^.

Peripheral white blood cells are an acceptable source of RNA for evaluating gene expression in response to drug or other biological exposure^32^. The changes in gene regulation patterns in the peripheral white blood cells, as a result of the 6-month supplementation of vitamin D_3_, represented alterations in cells that were short lived and that were constantly replenished by bone marrow precursors. Thus, changes in gene expression patterns after 6 months does not related to the same cells, due to the short life-cycle of circulating white blood cells.

However, there are several published transcriptome studies of peripheral white blood cells and purified monocytes obtained from adults that relate gene expression to vitamin D^33–35^.

In addition, the influence of vitamin D_3_ active metabolite, 1,25(OH)_2_D_3_, on broad gene expression in monocytes was evaluated by RNA-seq^36,37^. The study showed that about 9% of gene expression profile of monocytes were altered by of 1,25(OH)_2_D_3_^36^.

This research program emphasizes the importance of personalized medicine. Vitamin D supplementation at 10,000 IU/d for 6 months was safe, had optimally regulated PTH levels and a pronounced effect on genetic expression of more than 1,200 genes. Furthermore, broad gene expression may predict individual responsiveness to vitamin D_3_ supplementation.

## Methods

The protocol of the current study approved by the Institutional Review Board (IRB) of Boston University Medical Campus (H-35506) and was completed in accordance with the CONSORT statement^38^.

This clinical trial is listed at the ClinicalTrials.gov (NCT02856776; date of registration 05/08/2016). All participants provided written informed consent.

A deidentified bottle that contained 60 capsules of vitamin D_3_ was given to each participant that was formulated with one of the doses of vitamin D_3_ (600 IU/d, 4,000 IU/d or 10,000 IU/d). All vitamin D_3_ supplements were provided by Solgar Inc (Leonia, NJ) and the contents evaluated by HPLC as previously described^5^ and were found to contain concentrations within 10% of their specified content. The bottles were returned at each visit when the vitamin D_3_ capsules were counted to track compliance.

The participants were randomized by a computer-generated randomization program into one of the three study groups (Fig. 1)^35^.

### Recruitment

Healthy young adult males and non-pregnant females were prescreened for serum concentrations of 25(OH)D deemed insufficient (below 30 ng/mL). Inclusion criteria included healthy young healthy black and white adults with a BMI <30 kg/m^2^ without disorders or medications affecting vitamin D metabolism. To reduce the effect on 25(OH)D levels recruitment started in October and supplementation was completed by March. All subjects signed an informed accepted by the IRB of Boston University Medical Campus.

The exclusion criteria consistent with our previous study^8^ were: history of elevated serum calcium (>10.5 mg%); vitamin D supplementation with a dose of 600 IU/d or more; direct exposure to artificial UVB or solar radiation during the past month for greater than eight hours; any kind of malabsorption; history of chronic or acute renal or hepatic disease; current antiseizure medications or glucocorticoids; pregnant/lactating women; and reluctance to consent to the study.

### Study visits and blood sample collection

All participants visited in the GCRU (a unit for clinical research at Boston University Medical Campus). At baseline and every eight weeks, blood was drawn from each subject to determine serum calcium, albumin, creatinine, PTH and total 25(OH)D concentrations. At baseline and after 24 weeks additional blood was obtained for collecting the buffy coat for the RNA extraction to evaluate broad gene expression by microarray analysis. Buffy coat including peripheral white blood cells isolated as soon after the blood draw as possible (usually <30 minutes after the blood drawn) to obtain total RNA. Quest Diagnostics performed the 25(OH)D and PTH assays as previously described^4^. The other blood biochemistries were performed by Boston Medical Center Clinical Laboratory.

### Microarray analysis

RNA extraction was performed by using a QIAGEN RNA extraction kit (QIAGEN’s RNeasy kit; Qiagen, Valencia, CA). The isolated RNA kept at −80 degrees Celsius till using for expression analysis. All stored RNA sent to the Boston University Microarray Resource Facility for analysis. All procedures are described in the GeneChip® Whole Transcript (WT) Sense Target Labeling Assay Manual (Affymetrix, Santa Clara, CA) and also in our previous article^8^ and supplementary information.

### Real time –PCR verification

The microarray results of gene expression alterations were confirmed by real-time PCR using the Quant Studio 7 Real-Time PCR system. Total 7 genes including *HIST1H2BB, TLR1, CYP24A1, LRRN3, RPL3, OCLM* and *HBE1* selected for validation based on their differential expression in the Microarray analysis and 2 housekeeping genes (Table 2).

For 7 genes (Table 2). All procedures described in our previous article^8^ and supplementary information.

### Safety and compliance

The amount of vitamin D_3_ that given was within the guidelines recommended by the Institute of Medicine (now National Academy of Medicine) and the Endocrine Society Guidelines^1,3^. Serum calcium, albumin and creatinine were determined every 8 weeks to evaluate for potential toxicity. Any participant discovered to have serum calcium level above 10.5 mg/dL or serum creatinine increased by >20% would immediately be removed from the study and the primary care physician informed.

A deidentified bottle containing 60 capsules of vitamin D_3_ was given to each participant who had one of the doses of vitamin D_3_ (600 IU/d, 4,000 IU/d or 10,000 IU/d). The bottles were returned at each visit when the vitamin D_3_ capsules were counted to track compliance.

### Statistical and functional analysis

The microarray data were normalized by using RMA method and the quality control and similarity was checked by using Principal Component Analysis (PCA) method as described previously^8^. To compare the differentially expressed genes between groups, a 2-way ANOVA in the linear model was applied. A p value threshold of 0.05 and also a False Discovery Rate (FDR) of 0.1 considered as significant results. Pathway enrichment analysis was performed as previously described^8,39,40^.

### Power

The sample size of current clinical trial was calculated based on the data of our previous study of the impact of vitamin D on broad gene expression^8^. By considering 0.05 of a type 1 error(α) and power of 80%) and changing 2-fold change in gene expression based on previous results^8^ with a standard deviation of 1, the sample size (N) for was estimated to N = 4 for each arm. Assuming a 2-fold difference in gene expression between the groups being compared, 2 time points for comparison (baseline, and 24 weeks), with N = 4/arm, we are able to achieve >80% power. Assuming a dropout rate of 10–20% and design effect 1.5, we enrolled a minimum of 8 subjects per arm.

## Supplementary information

Supplementary Information

## Data Availability

The datasets are available in the Microarray & Sequencing Resource Core Facility repository and directly accessible at http://microarray.bu.edu/~yurik/HolickM/2018-02-27_Holick_Hu1-0ST.zip.
