# Supplementary material for: Disassociation of Vitamin D’s Calcemic Activity and Non-calcemic Genomic Activity and Individual Responsiveness: A Randomized Controlled Double-Blind Clinical Trial
Source: Sci Rep. 2019 Nov 27;9:17685. doi: 10.1038/s41598-019-53864-1 (PMC6881448; doi:10.1038/s41598-019-53864-1)
Supplement: Supplementary file 1 — Supplementary Information [file 41598_2019_53864_MOESM1_ESM.docx]

**Supplementary information:**

# **Disassociation of Vitamin D’s Calcemic Activity and Non-calcemic Genomic Activity and Individual Responsiveness: A Randomized Controlled Double-Blind Clinical Trial**

Arash Shirvani^1^, Tyler Arek Kalajian^1^, Anjeli Song^1^ and Michael. F. Holick^1,*^

^Boston University School of Medicine, Boston Medical Center, Section Endocrinology, Diabetes, Nutrition and Weight Management, Department of Medicine, Vitamin D, Skin, and Bone Research Laboratory, Boston, MA 02118 USA^

^Boston University School of Medicine, Boston Medical Center, Section Endocrinology, Diabetes, Nutrition and Weight Management, Department of Medicine, Vitamin D, Skin, and Bone Research Laboratory, Boston, MA 02118 USA^

^11^Boston University School of Medicine, Boston Medical Center, Section Endocrinology, Diabetes, Nutrition and Weight Management, Department of Medicine, Vitamin D, Skin, and Bone Research Laboratory, Boston, MA 02118 USA

*Corresponding Author:

Michael F. Holick, Ph.D., M.D.

Boston University School of Medicine

Vitamin D Lab

85 E Newton St. M-1013

Boston, MA, 02118

Email: [mfholick@bu.edu](mailto:mfholick@bu.edu)

Phone: 617-358-6139

**Figure 1: Heatmaps of vitamin D responsive genes whose expression levels change after 6 months’ vitamin D_3_ supplementation (4,000 IUs/day).** Two groups of gene-expression changes are seen based on upregulation (red) or downregulation (blue) of gene expression post vitamin D_3_ supplementation. (Colors ranged from blue to red; High expression = red, average expression = white, low expression = blue). (Right) Clustering of the 309 genes affected by 4,000 IUs/day vitamin D_3_ supplementation was based on upregulation (red) or down regulation (blue) of gene expression.


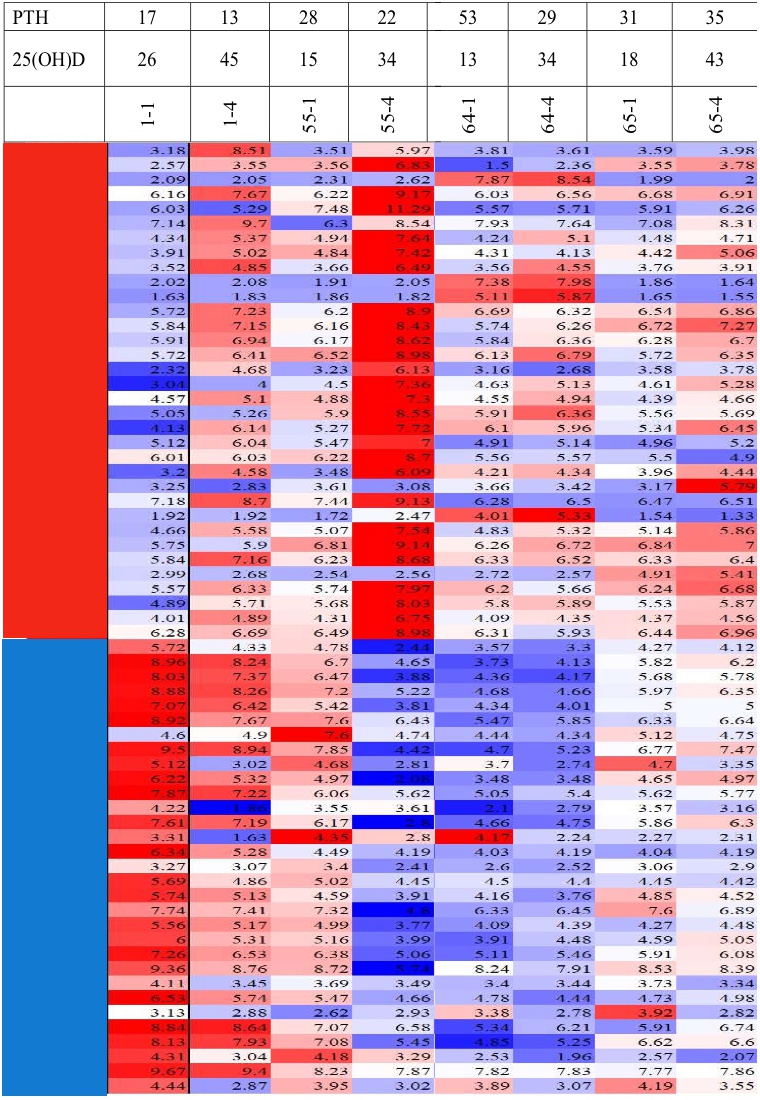


**Figure 2. Protein-protein interaction (PPI) network was constructed from differentially downregulated genes after vitamin D_3_ supplementation.** We mapped differentially downregulated genes to the STRING database (the hub protein was selected according to the node degree) and screened significant interactions with a score >0.7 The key genes in these clusters are TLR1, CD180 and LRRN3.


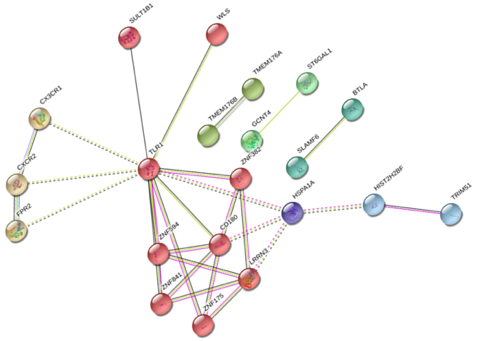


**Table 1. A list of vitamin D responsive genes whose expression levels upregulated after 6 months’ vitamin D_3_ supplementation (10,000 IU/d).**

| **Entrez Gene ID** | **Symbol** | **Description** |
| --- | --- | --- |
| [677822](http://www.ncbi.nlm.nih.gov/gene/677822) | SNORA40 | small nucleolar RNA, H/ACA box 40 |
| [84419](http://www.ncbi.nlm.nih.gov/gene/84419) | C15orf48 | chromosome 15 open reading frame 48 |
| [4929](http://www.ncbi.nlm.nih.gov/gene/4929) | NR4A2 | nuclear receptor subfamily 4, group A, member 2 |
| [677808](http://www.ncbi.nlm.nih.gov/gene/677808) | SNORA23 | small nucleolar RNA, H/ACA box 23 |
| [7128](http://www.ncbi.nlm.nih.gov/gene/7128) | TNFAIP3 | tumor necrosis factor, alpha-induced protein 3 |
| [643036](http://www.ncbi.nlm.nih.gov/gene/643036) | SLED1 | proteoglycan 3 pseudogene |
| [53831](http://www.ncbi.nlm.nih.gov/gene/53831) | GPR84 | G protein-coupled receptor 84 |
| [2920](http://www.ncbi.nlm.nih.gov/gene/2920) | CXCL2 | chemokine (C-X-C motif) ligand 2 |
| [29923](http://www.ncbi.nlm.nih.gov/gene/29923) | HILPDA | hypoxia inducible lipid droplet-associated |
| [100151683](http://www.ncbi.nlm.nih.gov/gene/100151683) | RNU4ATAC | RNA, U4atac small nuclear (U12-dependent splicing) |
| [407006](http://www.ncbi.nlm.nih.gov/gene/407006) | MIR221 | microRNA 221 |
| [3725](http://www.ncbi.nlm.nih.gov/gene/3725) | JUN | jun proto-oncogene |
| [26855](http://www.ncbi.nlm.nih.gov/gene/26855) | RNU2-2 | RNA, U2 small nuclear 2 |
| [5996](http://www.ncbi.nlm.nih.gov/gene/5996) | RGS1 | regulator of G-protein signaling 1 |
| [5187](http://www.ncbi.nlm.nih.gov/gene/5187) | PER1 | period homolog 1 (Drosophila) |
| [85391](http://www.ncbi.nlm.nih.gov/gene/85391) | SNORD14E | small nucleolar RNA, C/D box 14E |
| [692198](http://www.ncbi.nlm.nih.gov/gene/692198) | SNORD78 | small nucleolar RNA, C/D box 78 |
| [6044](http://www.ncbi.nlm.nih.gov/gene/6044) | SNORA62 | small nucleolar RNA, H/ACA box 62 |
| [3383](http://www.ncbi.nlm.nih.gov/gene/3383) | ICAM1 | intercellular adhesion molecule 1 |
| [84807](http://www.ncbi.nlm.nih.gov/gene/84807) | NFKBID | nuclear factor of kappa light polypeptide gene enhancer in B-cells inhibitor, delta |
| [9308](http://www.ncbi.nlm.nih.gov/gene/9308) | CD83 | CD83 molecule |
| [6083](http://www.ncbi.nlm.nih.gov/gene/6083) | SNORD21 | small nucleolar RNA, C/D box 21 |
| [5252](http://www.ncbi.nlm.nih.gov/gene/5252) | PHF1 | PHD finger protein 1 |
| [23645](http://www.ncbi.nlm.nih.gov/gene/23645) | PPP1R15A | protein phosphatase 1, regulatory subunit 15A |
| [50486](http://www.ncbi.nlm.nih.gov/gene/50486) | G0S2 | G0/G1switch 2 |
| [11221](http://www.ncbi.nlm.nih.gov/gene/11221) | DUSP10 | dual specificity phosphatase 10 |
| [1119](http://www.ncbi.nlm.nih.gov/gene/1119) | CHKA | choline kinase alpha |
| [3656](http://www.ncbi.nlm.nih.gov/gene/3656) | IRAK2 | interleukin-1 receptor-associated kinase 2 |
| [1263](http://www.ncbi.nlm.nih.gov/gene/1263) | PLK3 | polo-like kinase 3 |
| [94056](http://www.ncbi.nlm.nih.gov/gene/94056) | SYAP1 | synapse associated protein 1 |
| [25790](http://www.ncbi.nlm.nih.gov/gene/25790) | CCDC19 | coiled-coil domain containing 19 |
| [4616](http://www.ncbi.nlm.nih.gov/gene/4616) | GADD45B | growth arrest and DNA-damage-inducible, beta |
| [8360](http://www.ncbi.nlm.nih.gov/gene/8360) | HIST1H4D | histone cluster 1, H4d |
| [3576](http://www.ncbi.nlm.nih.gov/gene/3576) | IL8 | interleukin 8 |
| [10950](http://www.ncbi.nlm.nih.gov/gene/10950) | BTG3 | BTG family, member 3 |
| [6348](http://www.ncbi.nlm.nih.gov/gene/6348) | CCL3 | chemokine (C-C motif) ligand 3 |
| [54541](http://www.ncbi.nlm.nih.gov/gene/54541) | DDIT4 | DNA-damage-inducible transcript 4 |
| [4792](http://www.ncbi.nlm.nih.gov/gene/4792) | NFKBIA | nuclear factor of kappa light polypeptide gene enhancer in B-cells inhibitor, alpha |
| [2354](http://www.ncbi.nlm.nih.gov/gene/2354) | FOSB | FBJ murine osteosarcoma viral oncogene homolog B |
| [969](http://www.ncbi.nlm.nih.gov/gene/969) | CD69 | CD69 molecule |
| [64061](http://www.ncbi.nlm.nih.gov/gene/64061) | TSPYL2 | TSPY-like 2 |
| [80149](http://www.ncbi.nlm.nih.gov/gene/80149) | ZC3H12A | zinc finger CCCH-type containing 12A |
| [26815](http://www.ncbi.nlm.nih.gov/gene/26815) | SNORD36A | small nucleolar RNA, C/D box 36A |
| [26770](http://www.ncbi.nlm.nih.gov/gene/26770) | SNORD79 | small nucleolar RNA, C/D box 79 |
| [23308](http://www.ncbi.nlm.nih.gov/gene/23308) | ICOSLG | inducible T-cell co-stimulator ligand |
| [644975](http://www.ncbi.nlm.nih.gov/gene/644975) | FLJ30064 | uncharacterized LOC644975 |
| [8140](http://www.ncbi.nlm.nih.gov/gene/8140) | SLC7A5 | solute carrier family 7 (amino acid transporter light chain, L system), member 5 |
| [123](http://www.ncbi.nlm.nih.gov/gene/123) | PLIN2 | perilipin 2 |
| [85389](http://www.ncbi.nlm.nih.gov/gene/85389) | SNORD14C | small nucleolar RNA, C/D box 14C |
| [9929](http://www.ncbi.nlm.nih.gov/gene/9929) | JOSD1 | Josephin domain containing 1 |
| [51733](http://www.ncbi.nlm.nih.gov/gene/51733) | UPB1 | ureidopropionase, beta |
| [3627](http://www.ncbi.nlm.nih.gov/gene/3627) | CXCL10 | chemokine (C-X-C motif) ligand 10 |
| [55422](http://www.ncbi.nlm.nih.gov/gene/55422) | ZNF331 | zinc finger protein 331 |
| [200232](http://www.ncbi.nlm.nih.gov/gene/200232) | FAM209A | family with sequence similarity 209, member A |
| [135](http://www.ncbi.nlm.nih.gov/gene/135) | ADORA2A | adenosine A2a receptor |
| [998](http://www.ncbi.nlm.nih.gov/gene/998) | CDC42 | cell division cycle 42 (GTP binding protein, 25kDa) |
| [1649](http://www.ncbi.nlm.nih.gov/gene/1649) | DDIT3 | DNA-damage-inducible transcript 3 |
| [388799](http://www.ncbi.nlm.nih.gov/gene/388799) | FAM209B | family with sequence similarity 209, member B |
| [100124536](http://www.ncbi.nlm.nih.gov/gene/100124536) | SNORA38B | small nucleolar RNA, H/ACA box 38B |
| [9298](http://www.ncbi.nlm.nih.gov/gene/9298) | SNORD31 | small nucleolar RNA, C/D box 31 |
| [94163](http://www.ncbi.nlm.nih.gov/gene/94163) | SNORD38B | small nucleolar RNA, C/D box 38B |
| [4729](http://www.ncbi.nlm.nih.gov/gene/4729) | NDUFV2 | NADH dehydrogenase (ubiquinone) flavoprotein 2, 24kDa |
| [619498](http://www.ncbi.nlm.nih.gov/gene/619498) | SNORD74 | small nucleolar RNA, C/D box 74 |
| [23210](http://www.ncbi.nlm.nih.gov/gene/23210) | JMJD6 | jumonji domain containing 6 |
| [64651](http://www.ncbi.nlm.nih.gov/gene/64651) | CSRNP1 | cysteine-serine-rich nuclear protein 1 |
| [26817](http://www.ncbi.nlm.nih.gov/gene/26817) | SNORD34 | small nucleolar RNA, C/D box 34 |
| [677775](http://www.ncbi.nlm.nih.gov/gene/677775) | SCARNA5 | small Cajal body-specific RNA 5 |
| [677771](http://www.ncbi.nlm.nih.gov/gene/677771) | SCARNA4 | small Cajal body-specific RNA 4 |
| [4791](http://www.ncbi.nlm.nih.gov/gene/4791) | NFKB2 | nuclear factor of kappa light polypeptide gene enhancer in B-cells 2 (p49/p100) |
| [441194](http://www.ncbi.nlm.nih.gov/gene/441194) | PMS2CL | PMS2 C-terminal like pseudogene |
| [7071](http://www.ncbi.nlm.nih.gov/gene/7071) | KLF10 | Kruppel-like factor 10 |
| [79912](http://www.ncbi.nlm.nih.gov/gene/79912) | PYROXD1 | pyridine nucleotide-disulphide oxidoreductase domain 1 |
| [6018](http://www.ncbi.nlm.nih.gov/gene/6018) | RLF | rearranged L-myc fusion |
| [5328](http://www.ncbi.nlm.nih.gov/gene/5328) | PLAU | plasminogen activator, urokinase |
| [677820](http://www.ncbi.nlm.nih.gov/gene/677820) | SNORA38 | small nucleolar RNA, H/ACA box 38 |
| [64332](http://www.ncbi.nlm.nih.gov/gene/64332) | NFKBIZ | nuclear factor of kappa light polypeptide gene enhancer in B-cells inhibitor, zeta |
| [9302](http://www.ncbi.nlm.nih.gov/gene/9302) | SNORD26 | small nucleolar RNA, C/D box 26 |
| [7422](http://www.ncbi.nlm.nih.gov/gene/7422) | VEGFA | vascular endothelial growth factor A |
| [84919](http://www.ncbi.nlm.nih.gov/gene/84919) | PPP1R15B | protein phosphatase 1, regulatory subunit 15B |
| [84124](http://www.ncbi.nlm.nih.gov/gene/84124) | ZNF394 | zinc finger protein 394 |
| [9299](http://www.ncbi.nlm.nih.gov/gene/9299) | SNORD30 | small nucleolar RNA, C/D box 30 |
| [23130](http://www.ncbi.nlm.nih.gov/gene/23130) | ATG2A | autophagy related 2A |
| [100499177](http://www.ncbi.nlm.nih.gov/gene/100499177) | THAP9-AS1 | THAP9 antisense RNA 1 |
| [2107](http://www.ncbi.nlm.nih.gov/gene/2107) | ETF1 | eukaryotic translation termination factor 1 |
| [54882](http://www.ncbi.nlm.nih.gov/gene/54882) | ANKHD1 | ankyrin repeat and KH domain containing 1 |
| [6023](http://www.ncbi.nlm.nih.gov/gene/6023) | RMRP | RNA component of mitochondrial RNA processing endoribonuclease |
| [121504](http://www.ncbi.nlm.nih.gov/gene/121504) | HIST4H4 | histone cluster 4, H4 |
| [677823](http://www.ncbi.nlm.nih.gov/gene/677823) | SNORA42 | small nucleolar RNA, H/ACA box 42 |
| [6080](http://www.ncbi.nlm.nih.gov/gene/6080) | SNORA73A | small nucleolar RNA, H/ACA box 73A |
| [90637](http://www.ncbi.nlm.nih.gov/gene/90637) | ZFAND2A | zinc finger, AN1-type domain 2A |
| [26780](http://www.ncbi.nlm.nih.gov/gene/26780) | SNORA68 | small nucleolar RNA, H/ACA box 68 |
| [6690](http://www.ncbi.nlm.nih.gov/gene/6690) | SPINK1 | serine peptidase inhibitor, Kazal type 1 |
| [94161](http://www.ncbi.nlm.nih.gov/gene/94161) | SNORD46 | small nucleolar RNA, C/D box 46 |
| [79729](http://www.ncbi.nlm.nih.gov/gene/79729) | SH3D21 | SH3 domain containing 21 |
| [29080](http://www.ncbi.nlm.nih.gov/gene/29080) | CCDC59 | coiled-coil domain containing 59 |
| [49854](http://www.ncbi.nlm.nih.gov/gene/49854) | ZNF295 | zinc finger protein 295 |
| [9034](http://www.ncbi.nlm.nih.gov/gene/9034) | CCRL2 | chemokine (C-C motif) receptor-like 2 |
| [284695](http://www.ncbi.nlm.nih.gov/gene/284695) | ZNF326 | zinc finger protein 326 |
| [4794](http://www.ncbi.nlm.nih.gov/gene/4794) | NFKBIE | nuclear factor of kappa light polypeptide gene enhancer in B-cells inhibitor, epsilon |
| [692148](http://www.ncbi.nlm.nih.gov/gene/692148) | SCARNA10 | small Cajal body-specific RNA 10 |
| [114599](http://www.ncbi.nlm.nih.gov/gene/114599) | SNORD15B | small nucleolar RNA, C/D box 15B |
| [116938](http://www.ncbi.nlm.nih.gov/gene/116938) | SNORD83B | small nucleolar RNA, C/D box 83B |
| [57154](http://www.ncbi.nlm.nih.gov/gene/57154) | SMURF1 | SMAD specific E3 ubiquitin protein ligase 1 |
| [7718](http://www.ncbi.nlm.nih.gov/gene/7718) | ZNF165 | zinc finger protein 165 |
| [85495](http://www.ncbi.nlm.nih.gov/gene/85495) | RPPH1 | ribonuclease P RNA component H1 |
| [84879](http://www.ncbi.nlm.nih.gov/gene/84879) | MFSD2A | major facilitator superfamily domain containing 2A |
| [57471](http://www.ncbi.nlm.nih.gov/gene/57471) | ERMN | ermin, ERM-like protein |
| [602](http://www.ncbi.nlm.nih.gov/gene/602) | BCL3 | B-cell CLL/lymphoma 3 |
| [158830](http://www.ncbi.nlm.nih.gov/gene/158830) | CXorf65 | chromosome X open reading frame 65 |
| [8553](http://www.ncbi.nlm.nih.gov/gene/8553) | BHLHE40 | basic helix-loop-helix family, member e40 |
| [2769](http://www.ncbi.nlm.nih.gov/gene/2769) | GNA15 | guanine nucleotide binding protein (G protein), alpha 15 (Gq class) |
| [594837](http://www.ncbi.nlm.nih.gov/gene/594837) | SNORD101 | small nucleolar RNA, C/D box 101 |
| [7037](http://www.ncbi.nlm.nih.gov/gene/7037) | TFRC | transferrin receptor (p90, CD71) |
| [137964](http://www.ncbi.nlm.nih.gov/gene/137964) | AGPAT6 | 1-acylglycerol-3-phosphate O-acyltransferase 6 (lysophosphatidic acid acyltransferase, zeta) |
| [1839](http://www.ncbi.nlm.nih.gov/gene/1839) | HBEGF | heparin-binding EGF-like growth factor |
| [3939](http://www.ncbi.nlm.nih.gov/gene/3939) | LDHA | lactate dehydrogenase A |
| [619568](http://www.ncbi.nlm.nih.gov/gene/619568) | SNORA4 | small nucleolar RNA, H/ACA box 4 |
| [677826](http://www.ncbi.nlm.nih.gov/gene/677826) | SNORA45 | small nucleolar RNA, H/ACA box 45 |
| [84930](http://www.ncbi.nlm.nih.gov/gene/84930) | MASTL | microtubule associated serine/threonine kinase-like |
| [123720](http://www.ncbi.nlm.nih.gov/gene/123720) | WHAMM | WAS protein homolog associated with actin, golgi membranes and microtubules |
| [5366](http://www.ncbi.nlm.nih.gov/gene/5366) | PMAIP1 | phorbol-12-myristate-13-acetate-induced protein 1 |
| [113115](http://www.ncbi.nlm.nih.gov/gene/113115) | FAM54A | family with sequence similarity 54, member A |
| [10769](http://www.ncbi.nlm.nih.gov/gene/10769) | PLK2 | polo-like kinase 2 |
| [26824](http://www.ncbi.nlm.nih.gov/gene/26824) | RNU11 | RNA, U11 small nuclear |
| [9249](http://www.ncbi.nlm.nih.gov/gene/9249) | DHRS3 | dehydrogenase/reductase (SDR family) member 3 |
| [59348](http://www.ncbi.nlm.nih.gov/gene/59348) | ZNF350 | zinc finger protein 350 |
| [340529](http://www.ncbi.nlm.nih.gov/gene/340529) | PABPC1L2A | poly(A) binding protein, cytoplasmic 1-like 2A |
| [25764](http://www.ncbi.nlm.nih.gov/gene/25764) | C15orf63 | chromosome 15 open reading frame 63 |
| [55361](http://www.ncbi.nlm.nih.gov/gene/55361) | PI4K2A | phosphatidylinositol 4-kinase type 2 alpha |
| [92344](http://www.ncbi.nlm.nih.gov/gene/92344) | GORAB | golgin, RAB6-interacting |
| [677840](http://www.ncbi.nlm.nih.gov/gene/677840) | SNORA71D | small nucleolar RNA, H/ACA box 71D |
| [5004](http://www.ncbi.nlm.nih.gov/gene/5004) | ORM1 | orosomucoid 1 |
| [100533106](http://www.ncbi.nlm.nih.gov/gene/100533106) | ZHX1-C8ORF76 | ZHX1-C8ORF76 readthrough |
| [90799](http://www.ncbi.nlm.nih.gov/gene/90799) | CEP95 | centrosomal protein 95kDa |
| [8767](http://www.ncbi.nlm.nih.gov/gene/8767) | RIPK2 | receptor-interacting serine-threonine kinase 2 |
| [1073](http://www.ncbi.nlm.nih.gov/gene/1073) | CFL2 | cofilin 2 (muscle) |
| [84270](http://www.ncbi.nlm.nih.gov/gene/84270) | C9orf89 | chromosome 9 open reading frame 89 |
| [677781](http://www.ncbi.nlm.nih.gov/gene/677781) | SCARNA16 | small Cajal body-specific RNA 16 |
| [407007](http://www.ncbi.nlm.nih.gov/gene/407007) | MIR222 | microRNA 222 |
| [9297](http://www.ncbi.nlm.nih.gov/gene/9297) | SNORD29 | small nucleolar RNA, C/D box 29 |
| [26801](http://www.ncbi.nlm.nih.gov/gene/26801) | SNORD48 | small nucleolar RNA, C/D box 48 |
| [3458](http://www.ncbi.nlm.nih.gov/gene/3458) | IFNG | interferon, gamma |
| [133746](http://www.ncbi.nlm.nih.gov/gene/133746) | JMY | junction mediating and regulatory protein, p53 cofactor |
| [402778](http://www.ncbi.nlm.nih.gov/gene/402778) | IFITM10 | interferon induced transmembrane protein 10 |
| [3557](http://www.ncbi.nlm.nih.gov/gene/3557) | IL1RN | interleukin 1 receptor antagonist |
| [9188](http://www.ncbi.nlm.nih.gov/gene/9188) | DDX21 | DEAD (Asp-Glu-Ala-Asp) box helicase 21 |
| [55802](http://www.ncbi.nlm.nih.gov/gene/55802) | DCP1A | DCP1 decapping enzyme homolog A (S. cerevisiae) |
| [195827](http://www.ncbi.nlm.nih.gov/gene/195827) | AAED1 | AhpC/TSA antioxidant enzyme domain containing 1 |
| [26781](http://www.ncbi.nlm.nih.gov/gene/26781) | SNORA67 | small nucleolar RNA, H/ACA box 67 |
| [23521](http://www.ncbi.nlm.nih.gov/gene/23521) | RPL13A | ribosomal protein L13a |
| [57683](http://www.ncbi.nlm.nih.gov/gene/57683) | ZDBF2 | zinc finger, DBF-type containing 2 |
| [10912](http://www.ncbi.nlm.nih.gov/gene/10912) | GADD45G | growth arrest and DNA-damage-inducible, gamma |
| [56681](http://www.ncbi.nlm.nih.gov/gene/56681) | SAR1A | SAR1 homolog A (S. cerevisiae) |
| [133](http://www.ncbi.nlm.nih.gov/gene/133) | ADM | adrenomedullin |
| [25977](http://www.ncbi.nlm.nih.gov/gene/25977) | NECAP1 | NECAP endocytosis associated 1 |
| [619505](http://www.ncbi.nlm.nih.gov/gene/619505) | SNORA21 | small nucleolar RNA, H/ACA box 21 |
| [27342](http://www.ncbi.nlm.nih.gov/gene/27342) | RABGEF1 | RAB guanine nucleotide exchange factor (GEF) 1 |
| [388272](http://www.ncbi.nlm.nih.gov/gene/388272) | C16orf87 | chromosome 16 open reading frame 87 |
| [26792](http://www.ncbi.nlm.nih.gov/gene/26792) | SNORD57 | small nucleolar RNA, C/D box 57 |
| [150094](http://www.ncbi.nlm.nih.gov/gene/150094) | SIK1 | salt-inducible kinase 1 |
| [5837](http://www.ncbi.nlm.nih.gov/gene/5837) | PYGM | phosphorylase, glycogen, muscle |
| [8361](http://www.ncbi.nlm.nih.gov/gene/8361) | HIST1H4F | histone cluster 1, H4f |
| [340206](http://www.ncbi.nlm.nih.gov/gene/340206) | TREML3P | triggering receptor expressed on myeloid cells-like 3, pseudogene |
| [51444](http://www.ncbi.nlm.nih.gov/gene/51444) | RNF138 | ring finger protein 138, E3 ubiquitin protein ligase |
| [51602](http://www.ncbi.nlm.nih.gov/gene/51602) | NOP58 | NOP58 ribonucleoprotein homolog (yeast) |
| [317772](http://www.ncbi.nlm.nih.gov/gene/317772) | HIST2H2AB | histone cluster 2, H2ab |
| [51429](http://www.ncbi.nlm.nih.gov/gene/51429) | SNX9 | sorting nexin 9 |
| [339883](http://www.ncbi.nlm.nih.gov/gene/339883) | C3orf35 | chromosome 3 open reading frame 35 |
| [114049](http://www.ncbi.nlm.nih.gov/gene/114049) | WBSCR22 | Williams Beuren syndrome chromosome region 22 |
| [196383](http://www.ncbi.nlm.nih.gov/gene/196383) | RILPL2 | Rab interacting lysosomal protein-like 2 |
| [5127](http://www.ncbi.nlm.nih.gov/gene/5127) | CDK16 | cyclin-dependent kinase 16 |
| [406896](http://www.ncbi.nlm.nih.gov/gene/406896) | MIR103A2 | microRNA 103a-2 |
| [8731](http://www.ncbi.nlm.nih.gov/gene/8731) | RNMT | RNA (guanine-7-) methyltransferase |
| [51119](http://www.ncbi.nlm.nih.gov/gene/51119) | SBDS | Shwachman-Bodian-Diamond syndrome |
| [7525](http://www.ncbi.nlm.nih.gov/gene/7525) | YES1 | v-yes-1 Yamaguchi sarcoma viral oncogene homolog 1 |
| [90011](http://www.ncbi.nlm.nih.gov/gene/90011) | KIR3DX1 | killer cell immunoglobulin-like receptor, three domains, X1 |
| [8336](http://www.ncbi.nlm.nih.gov/gene/8336) | HIST1H2AM | histone cluster 1, H2am |
| [3306](http://www.ncbi.nlm.nih.gov/gene/3306) | HSPA2 | heat shock 70kDa protein 2 |
| [548645](http://www.ncbi.nlm.nih.gov/gene/548645) | DNAJC25 | DnaJ (Hsp40) homolog, subfamily C , member 25 |
| [6782](http://www.ncbi.nlm.nih.gov/gene/6782) | HSPA13 | heat shock protein 70kDa family, member 13 |
| [25855](http://www.ncbi.nlm.nih.gov/gene/25855) | BRMS1 | breast cancer metastasis suppressor 1 |
| [26810](http://www.ncbi.nlm.nih.gov/gene/26810) | SNORD41 | small nucleolar RNA, C/D box 41 |
| [54557](http://www.ncbi.nlm.nih.gov/gene/54557) | SGTB | small glutamine-rich tetratricopeptide repeat (TPR)-containing, beta |
| [57602](http://www.ncbi.nlm.nih.gov/gene/57602) | USP36 | ubiquitin specific peptidase 36 |
| [4256](http://www.ncbi.nlm.nih.gov/gene/4256) | MGP | matrix Gla protein |
| [6434](http://www.ncbi.nlm.nih.gov/gene/6434) | TRA2B | transformer 2 beta homolog (Drosophila) |
| [692088](http://www.ncbi.nlm.nih.gov/gene/692088) | SNORD50B | small nucleolar RNA, C/D box 50B |
| [8870](http://www.ncbi.nlm.nih.gov/gene/8870) | IER3 | immediate early response 3 |
| [6090](http://www.ncbi.nlm.nih.gov/gene/6090) | RNY5 | RNA, Ro-associated Y5 |
| [22936](http://www.ncbi.nlm.nih.gov/gene/22936) | ELL2 | elongation factor, RNA polymerase II, 2 |
| [2923](http://www.ncbi.nlm.nih.gov/gene/2923) | PDIA3 | protein disulfide isomerase family A, member 3 |
| [319139](http://www.ncbi.nlm.nih.gov/gene/319139) | SNORD56B | small nucleolar RNA, C/D box 56B |
| [11040](http://www.ncbi.nlm.nih.gov/gene/11040) | PIM2 | pim-2 oncogene |
| [692075](http://www.ncbi.nlm.nih.gov/gene/692075) | SNORD6 | small nucleolar RNA, C/D box 6 |
| [6617](http://www.ncbi.nlm.nih.gov/gene/6617) | SNAPC1 | small nuclear RNA activating complex, polypeptide 1, 43kDa |
| [677837](http://www.ncbi.nlm.nih.gov/gene/677837) | SNORA60 | small nucleolar RNA, H/ACA box 60 |
| [64376](http://www.ncbi.nlm.nih.gov/gene/64376) | IKZF5 | IKAROS family zinc finger 5 (Pegasus) |
| [8367](http://www.ncbi.nlm.nih.gov/gene/8367) | HIST1H4E | histone cluster 1, H4e |
| [5966](http://www.ncbi.nlm.nih.gov/gene/5966) | REL | v-rel reticuloendotheliosis viral oncogene homolog (avian) |
| [64092](http://www.ncbi.nlm.nih.gov/gene/64092) | SAMSN1 | SAM domain, SH3 domain and nuclear localization signals 1 |
| [7187](http://www.ncbi.nlm.nih.gov/gene/7187) | TRAF3 | TNF receptor-associated factor 3 |
| [1105](http://www.ncbi.nlm.nih.gov/gene/1105) | CHD1 | chromodomain helicase DNA binding protein 1 |
| [7124](http://www.ncbi.nlm.nih.gov/gene/7124) | TNF | tumor necrosis factor |
| [56965](http://www.ncbi.nlm.nih.gov/gene/56965) | PARP6 | poly (ADP-ribose) polymerase family, member 6 |
| [4814](http://www.ncbi.nlm.nih.gov/gene/4814) | NINJ1 | ninjurin 1 |
| [64940](http://www.ncbi.nlm.nih.gov/gene/64940) | STAG3L4 | stromal antigen 3-like 4 |
| [3091](http://www.ncbi.nlm.nih.gov/gene/3091) | HIF1A | hypoxia inducible factor 1, alpha subunit (basic helix-loop-helix transcription factor) |
| [26795](http://www.ncbi.nlm.nih.gov/gene/26795) | SNORD54 | small nucleolar RNA, C/D box 54 |
| [1407](http://www.ncbi.nlm.nih.gov/gene/1407) | CRY1 | cryptochrome 1 (photolyase-like) |
| [26813](http://www.ncbi.nlm.nih.gov/gene/26813) | SNORD36C | small nucleolar RNA, C/D box 36C |
| [91746](http://www.ncbi.nlm.nih.gov/gene/91746) | YTHDC1 | YTH domain containing 1 |
| [55234](http://www.ncbi.nlm.nih.gov/gene/55234) | SMU1 | smu-1 suppressor of mec-8 and unc-52 homolog (C. elegans) |
| [26809](http://www.ncbi.nlm.nih.gov/gene/26809) | SNORD42A | small nucleolar RNA, C/D box 42A |
| [2907](http://www.ncbi.nlm.nih.gov/gene/2907) | GRINA | glutamate receptor, ionotropic, N-methyl D-aspartate-associated protein 1 (glutamate binding) |
| [8341](http://www.ncbi.nlm.nih.gov/gene/8341) | HIST1H2BN | histone cluster 1, H2bn |
| [10561](http://www.ncbi.nlm.nih.gov/gene/10561) | IFI44 | interferon-induced protein 44 |
| [3018](http://www.ncbi.nlm.nih.gov/gene/3018) | HIST1H2BB | histone cluster 1, H2bb |
| [55937](http://www.ncbi.nlm.nih.gov/gene/55937) | APOM | apolipoprotein M |
| [4793](http://www.ncbi.nlm.nih.gov/gene/4793) | NFKBIB | nuclear factor of kappa light polypeptide gene enhancer in B-cells inhibitor, beta |
| [1906](http://www.ncbi.nlm.nih.gov/gene/1906) | EDN1 | endothelin 1 |
| [221079](http://www.ncbi.nlm.nih.gov/gene/221079) | ARL5B | ADP-ribosylation factor-like 5B |
| [6935](http://www.ncbi.nlm.nih.gov/gene/6935) | ZEB1 | zinc finger E-box binding homeobox 1 |
| [8878](http://www.ncbi.nlm.nih.gov/gene/8878) | SQSTM1 | sequestosome 1 |
| [23710](http://www.ncbi.nlm.nih.gov/gene/23710) | GABARAPL1 | GABA(A) receptor-associated protein like 1 |
| [10318](http://www.ncbi.nlm.nih.gov/gene/10318) | TNIP1 | TNFAIP3 interacting protein 1 |
| [57414](http://www.ncbi.nlm.nih.gov/gene/57414) | RHBDD2 | rhomboid domain containing 2 |
| [7185](http://www.ncbi.nlm.nih.gov/gene/7185) | TRAF1 | TNF receptor-associated factor 1 |
| [8342](http://www.ncbi.nlm.nih.gov/gene/8342) | HIST1H2BM | histone cluster 1, H2bm |
| [904](http://www.ncbi.nlm.nih.gov/gene/904) | CCNT1 | cyclin T1 |
| [26807](http://www.ncbi.nlm.nih.gov/gene/26807) | SNORD43 | small nucleolar RNA, C/D box 43 |
| [11171](http://www.ncbi.nlm.nih.gov/gene/11171) | STRAP | serine/threonine kinase receptor associated protein |
| [5713](http://www.ncbi.nlm.nih.gov/gene/5713) | PSMD7 | proteasome (prosome, macropain) 26S subunit, non-ATPase, 7 |
| [57162](http://www.ncbi.nlm.nih.gov/gene/57162) | PELI1 | pellino E3 ubiquitin protein ligase 1 |
| [5329](http://www.ncbi.nlm.nih.gov/gene/5329) | PLAUR | plasminogen activator, urokinase receptor |
| [652965](http://www.ncbi.nlm.nih.gov/gene/652965) | SNORA48 | small nucleolar RNA, H/ACA box 48 |
| [25917](http://www.ncbi.nlm.nih.gov/gene/25917) | THUMPD3 | THUMP domain containing 3 |
| [9792](http://www.ncbi.nlm.nih.gov/gene/9792) | SERTAD2 | SERTA domain containing 2 |
| [84749](http://www.ncbi.nlm.nih.gov/gene/84749) | USP30 | ubiquitin specific peptidase 30 |
| [134429](http://www.ncbi.nlm.nih.gov/gene/134429) | STARD4 | StAR-related lipid transfer (START) domain containing 4 |
| [6210](http://www.ncbi.nlm.nih.gov/gene/6210) | RPS15A | ribosomal protein S15a |
| [51537](http://www.ncbi.nlm.nih.gov/gene/51537) | MTFP1 | mitochondrial fission process 1 |
| [127829](http://www.ncbi.nlm.nih.gov/gene/127829) | ARL8A | ADP-ribosylation factor-like 8A |
| [3096](http://www.ncbi.nlm.nih.gov/gene/3096) | HIVEP1 | human immunodeficiency virus type I enhancer binding protein 1 |
| [353322](http://www.ncbi.nlm.nih.gov/gene/353322) | ANKRD37 | ankyrin repeat domain 37 |
| [9304](http://www.ncbi.nlm.nih.gov/gene/9304) | SNORD22 | small nucleolar RNA, C/D box 22 |
| [58496](http://www.ncbi.nlm.nih.gov/gene/58496) | LY6G5B | lymphocyte antigen 6 complex, locus G5B |
| [4189](http://www.ncbi.nlm.nih.gov/gene/4189) | DNAJB9 | DnaJ (Hsp40) homolog, subfamily B, member 9 |
| [54883](http://www.ncbi.nlm.nih.gov/gene/54883) | CWC25 | CWC25 spliceosome-associated protein homolog (S. cerevisiae) |
| [84248](http://www.ncbi.nlm.nih.gov/gene/84248) | FYTTD1 | forty-two-three domain containing 1 |
| [8366](http://www.ncbi.nlm.nih.gov/gene/8366) | HIST1H4B | histone cluster 1, H4b |
| [51256](http://www.ncbi.nlm.nih.gov/gene/51256) | TBC1D7 | TBC1 domain family, member 7 |
| [8345](http://www.ncbi.nlm.nih.gov/gene/8345) | HIST1H2BH | histone cluster 1, H2bh |
| [3313](http://www.ncbi.nlm.nih.gov/gene/3313) | HSPA9 | heat shock 70kDa protein 9 (mortalin) |
| [8896](http://www.ncbi.nlm.nih.gov/gene/8896) | BUD31 | BUD31 homolog (S. cerevisiae) |
| [84981](http://www.ncbi.nlm.nih.gov/gene/84981) | MIR22HG | MIR22 host gene (non-protein coding) |
| [200186](http://www.ncbi.nlm.nih.gov/gene/200186) | CRTC2 | CREB regulated transcription coactivator 2 |
| [8638](http://www.ncbi.nlm.nih.gov/gene/8638) | OASL | 2'-5'-oligoadenylate synthetase-like |
| [9303](http://www.ncbi.nlm.nih.gov/gene/9303) | SNORD25 | small nucleolar RNA, C/D box 25 |
| [64960](http://www.ncbi.nlm.nih.gov/gene/64960) | MRPS15 | mitochondrial ribosomal protein S15 |
| [762](http://www.ncbi.nlm.nih.gov/gene/762) | CA4 | carbonic anhydrase IV |
| [6130](http://www.ncbi.nlm.nih.gov/gene/6130) | RPL7A | ribosomal protein L7a |
| [55818](http://www.ncbi.nlm.nih.gov/gene/55818) | KDM3A | lysine (K)-specific demethylase 3A |
| [64412](http://www.ncbi.nlm.nih.gov/gene/64412) | GZF1 | GDNF-inducible zinc finger protein 1 |
| [6189](http://www.ncbi.nlm.nih.gov/gene/6189) | RPS3A | ribosomal protein S3A |
| [2010](http://www.ncbi.nlm.nih.gov/gene/2010) | EMD | emerin |
| [57062](http://www.ncbi.nlm.nih.gov/gene/57062) | DDX24 | DEAD (Asp-Glu-Ala-Asp) box polypeptide 24 |
| [10010](http://www.ncbi.nlm.nih.gov/gene/10010) | TANK | TRAF family member-associated NFKB activator |
| [10043](http://www.ncbi.nlm.nih.gov/gene/10043) | TOM1 | target of myb1 (chicken) |
| [55081](http://www.ncbi.nlm.nih.gov/gene/55081) | IFT57 | intraflagellar transport 57 homolog (Chlamydomonas) |
| [85403](http://www.ncbi.nlm.nih.gov/gene/85403) | EAF1 | ELL associated factor 1 |
| [340542](http://www.ncbi.nlm.nih.gov/gene/340542) | BEX5 | brain expressed, X-linked 5 |
| [39](http://www.ncbi.nlm.nih.gov/gene/39) | ACAT2 | acetyl-CoA acetyltransferase 2 |
| [8233](http://www.ncbi.nlm.nih.gov/gene/8233) | ZRSR2 | zinc finger (CCCH type), RNA-binding motif and serine/arginine rich 2 |
| [64419](http://www.ncbi.nlm.nih.gov/gene/64419) | MTMR14 | myotubularin related protein 14 |
| [2553](http://www.ncbi.nlm.nih.gov/gene/2553) | GABPB1 | GA binding protein transcription factor, beta subunit 1 |
| [26769](http://www.ncbi.nlm.nih.gov/gene/26769) | SNORD81 | small nucleolar RNA, C/D box 81 |
| [54210](http://www.ncbi.nlm.nih.gov/gene/54210) | TREM1 | triggering receptor expressed on myeloid cells 1 |
| [402573](http://www.ncbi.nlm.nih.gov/gene/402573) | C7orf61 | chromosome 7 open reading frame 61 |
| [26355](http://www.ncbi.nlm.nih.gov/gene/26355) | FAM162A | family with sequence similarity 162, member A |
| [26292](http://www.ncbi.nlm.nih.gov/gene/26292) | MYCBP | c-myc binding protein |
| [25976](http://www.ncbi.nlm.nih.gov/gene/25976) | TIPARP | TCDD-inducible poly(ADP-ribose) polymerase |
| [4974](http://www.ncbi.nlm.nih.gov/gene/4974) | OMG | oligodendrocyte myelin glycoprotein |
| [5631](http://www.ncbi.nlm.nih.gov/gene/5631) | PRPS1 | phosphoribosyl pyrophosphate synthetase 1 |
| [142891](http://www.ncbi.nlm.nih.gov/gene/142891) | SAMD8 | sterile alpha motif domain containing 8 |
| [3985](http://www.ncbi.nlm.nih.gov/gene/3985) | LIMK2 | LIM domain kinase 2 |
| [246243](http://www.ncbi.nlm.nih.gov/gene/246243) | RNASEH1 | ribonuclease H1 |
| [79630](http://www.ncbi.nlm.nih.gov/gene/79630) | C1orf54 | chromosome 1 open reading frame 54 |
| [10534](http://www.ncbi.nlm.nih.gov/gene/10534) | SSSCA1 | Sjogren syndrome/scleroderma autoantigen 1 |
| [57018](http://www.ncbi.nlm.nih.gov/gene/57018) | CCNL1 | cyclin L1 |
| [79786](http://www.ncbi.nlm.nih.gov/gene/79786) | KLHL36 | kelch-like 36 (Drosophila) |
| [29105](http://www.ncbi.nlm.nih.gov/gene/29105) | C16orf80 | chromosome 16 open reading frame 80 |
| [51526](http://www.ncbi.nlm.nih.gov/gene/51526) | C20orf111 | chromosome 20 open reading frame 111 |
| [5209](http://www.ncbi.nlm.nih.gov/gene/5209) | PFKFB3 | 6-phosphofructo-2-kinase/fructose-2,6-biphosphatase 3 |
| [51726](http://www.ncbi.nlm.nih.gov/gene/51726) | DNAJB11 | DnaJ (Hsp40) homolog, subfamily B, member 11 |
| [8351](http://www.ncbi.nlm.nih.gov/gene/8351) | HIST1H3D | histone cluster 1, H3d |
| [399](http://www.ncbi.nlm.nih.gov/gene/399) | RHOH | ras homolog family member H |
| [11319](http://www.ncbi.nlm.nih.gov/gene/11319) | ECD | ecdysoneless homolog (Drosophila) |
| [27289](http://www.ncbi.nlm.nih.gov/gene/27289) | RND1 | Rho family GTPase 1 |
| [388701](http://www.ncbi.nlm.nih.gov/gene/388701) | C1orf189 | chromosome 1 open reading frame 189 |
| [114915](http://www.ncbi.nlm.nih.gov/gene/114915) | EPB41L4A-AS1 | EPB41L4A antisense RNA 1 |
| [692233](http://www.ncbi.nlm.nih.gov/gene/692233) | SNORD117 | small nucleolar RNA, C/D box 117 |
| [2204](http://www.ncbi.nlm.nih.gov/gene/2204) | FCAR | Fc fragment of IgA, receptor for |
| [219988](http://www.ncbi.nlm.nih.gov/gene/219988) | PATL1 | protein associated with topoisomerase II homolog 1 (yeast) |
| [1111](http://www.ncbi.nlm.nih.gov/gene/1111) | CHEK1 | checkpoint kinase 1 |
| [26799](http://www.ncbi.nlm.nih.gov/gene/26799) | SNORD50A | small nucleolar RNA, C/D box 50A |
| [9296](http://www.ncbi.nlm.nih.gov/gene/9296) | ATP6V1F | ATPase, H+ transporting, lysosomal 14kDa, V1 subunit F |
| [135458](http://www.ncbi.nlm.nih.gov/gene/135458) | HUS1B | HUS1 checkpoint homolog b (S. pombe) |
| [84444](http://www.ncbi.nlm.nih.gov/gene/84444) | DOT1L | DOT1-like, histone H3 methyltransferase (S. cerevisiae) |
| [5971](http://www.ncbi.nlm.nih.gov/gene/5971) | RELB | v-rel reticuloendotheliosis viral oncogene homolog B |
| [29946](http://www.ncbi.nlm.nih.gov/gene/29946) | SERTAD3 | SERTA domain containing 3 |
| [54520](http://www.ncbi.nlm.nih.gov/gene/54520) | CCDC93 | coiled-coil domain containing 93 |
| [619570](http://www.ncbi.nlm.nih.gov/gene/619570) | SNORD95 | small nucleolar RNA, C/D box 95 |
| [29066](http://www.ncbi.nlm.nih.gov/gene/29066) | ZC3H7A | zinc finger CCCH-type containing 7A |
| [121793](http://www.ncbi.nlm.nih.gov/gene/121793) | TEX29 | testis expressed 29 |
| [9921](http://www.ncbi.nlm.nih.gov/gene/9921) | RNF10 | ring finger protein 10 |
| [1106](http://www.ncbi.nlm.nih.gov/gene/1106) | CHD2 | chromodomain helicase DNA binding protein 2 |
| [25983](http://www.ncbi.nlm.nih.gov/gene/25983) | NGDN | neuroguidin, EIF4E binding protein |
| [1026](http://www.ncbi.nlm.nih.gov/gene/1026) | CDKN1A | cyclin-dependent kinase inhibitor 1A (p21, Cip1) |
| [5597](http://www.ncbi.nlm.nih.gov/gene/5597) | MAPK6 | mitogen-activated protein kinase 6 |
| [201627](http://www.ncbi.nlm.nih.gov/gene/201627) | FAM116A | family with sequence similarity 116, member A |
| [51389](http://www.ncbi.nlm.nih.gov/gene/51389) | RWDD1 | RWD domain containing 1 |
| [148022](http://www.ncbi.nlm.nih.gov/gene/148022) | TICAM1 | toll-like receptor adaptor molecule 1 |
| [150776](http://www.ncbi.nlm.nih.gov/gene/150776) | LOC150776 | sphingomyelin phosphodiesterase 4, neutral membrane (neutral sphingomyelinase-3) pseudogene |
| [5970](http://www.ncbi.nlm.nih.gov/gene/5970) | RELA | v-rel reticuloendotheliosis viral oncogene homolog A (avian) |
| [10519](http://www.ncbi.nlm.nih.gov/gene/10519) | CIB1 | calcium and integrin binding 1 (calmyrin) |
| [5805](http://www.ncbi.nlm.nih.gov/gene/5805) | PTS | 6-pyruvoyltetrahydropterin synthase |
| [26819](http://www.ncbi.nlm.nih.gov/gene/26819) | SNORD32A | small nucleolar RNA, C/D box 32A |
| [6095](http://www.ncbi.nlm.nih.gov/gene/6095) | RORA | RAR-related orphan receptor A |
| [51303](http://www.ncbi.nlm.nih.gov/gene/51303) | FKBP11 | FK506 binding protein 11, 19 kDa |
| [5005](http://www.ncbi.nlm.nih.gov/gene/5005) | ORM2 | orosomucoid 2 |
| [1915](http://www.ncbi.nlm.nih.gov/gene/1915) | EEF1A1 | eukaryotic translation elongation factor 1 alpha 1 |
| [80895](http://www.ncbi.nlm.nih.gov/gene/80895) | ILKAP | integrin-linked kinase-associated serine/threonine phosphatase |
| [94120](http://www.ncbi.nlm.nih.gov/gene/94120) | SYTL3 | synaptotagmin-like 3 |
| [729080](http://www.ncbi.nlm.nih.gov/gene/729080) | LOC729080 | glycine cleavage system protein H (aminomethyl carrier) pseudogene |
| [8340](http://www.ncbi.nlm.nih.gov/gene/8340) | HIST1H2BL | histone cluster 1, H2bl |
| [54331](http://www.ncbi.nlm.nih.gov/gene/54331) | GNG2 | guanine nucleotide binding protein (G protein), gamma 2 |
| [10226](http://www.ncbi.nlm.nih.gov/gene/10226) | PLIN3 | perilipin 3 |
| [3809](http://www.ncbi.nlm.nih.gov/gene/3809) | KIR2DS4 | killer cell immunoglobulin-like receptor, two domains, short cytoplasmic tail, 4 |
| [10668](http://www.ncbi.nlm.nih.gov/gene/10668) | CGRRF1 | cell growth regulator with ring finger domain 1 |
| [3659](http://www.ncbi.nlm.nih.gov/gene/3659) | IRF1 | interferon regulatory factor 1 |
| [3337](http://www.ncbi.nlm.nih.gov/gene/3337) | DNAJB1 | DnaJ (Hsp40) homolog, subfamily B, member 1 |
| [619499](http://www.ncbi.nlm.nih.gov/gene/619499) | SNORA27 | small nucleolar RNA, H/ACA box 27 |
| [256126](http://www.ncbi.nlm.nih.gov/gene/256126) | SYCE2 | synaptonemal complex central element protein 2 |
| [200298](http://www.ncbi.nlm.nih.gov/gene/200298) | LINC00528 | long intergenic non-protein coding RNA 528 |
| [51296](http://www.ncbi.nlm.nih.gov/gene/51296) | SLC15A3 | solute carrier family 15, member 3 |
| [2355](http://www.ncbi.nlm.nih.gov/gene/2355) | FOSL2 | FOS-like antigen 2 |
| [5553](http://www.ncbi.nlm.nih.gov/gene/5553) | PRG2 | proteoglycan 2, bone marrow (natural killer cell activator, eosinophil granule major basic protein) |
| [51690](http://www.ncbi.nlm.nih.gov/gene/51690) | LSM7 | LSM7 homolog, U6 small nuclear RNA associated (S. cerevisiae) |
| [84844](http://www.ncbi.nlm.nih.gov/gene/84844) | PHF5A | PHD finger protein 5A |
| [55905](http://www.ncbi.nlm.nih.gov/gene/55905) | RNF114 | ring finger protein 114 |
| [57599](http://www.ncbi.nlm.nih.gov/gene/57599) | WDR48 | WD repeat domain 48 |
| [5816](http://www.ncbi.nlm.nih.gov/gene/5816) | PVALB | parvalbumin |
| [8354](http://www.ncbi.nlm.nih.gov/gene/8354) | HIST1H3I | histone cluster 1, H3i |
| [2029](http://www.ncbi.nlm.nih.gov/gene/2029) | ENSA | endosulfine alpha |
| [23207](http://www.ncbi.nlm.nih.gov/gene/23207) | PLEKHM2 | pleckstrin homology domain containing, family M (with RUN domain) member 2 |
| [84002](http://www.ncbi.nlm.nih.gov/gene/84002) | B3GNT5 | UDP-GlcNAc:betaGal beta-1,3-N-acetylglucosaminyltransferase 5 |
| [3298](http://www.ncbi.nlm.nih.gov/gene/3298) | HSF2 | heat shock transcription factor 2 |
| [9337](http://www.ncbi.nlm.nih.gov/gene/9337) | CNOT8 | CCR4-NOT transcription complex, subunit 8 |
| [9689](http://www.ncbi.nlm.nih.gov/gene/9689) | BZW1 | basic leucine zipper and W2 domains 1 |
| [79796](http://www.ncbi.nlm.nih.gov/gene/79796) | ALG9 | asparagine-linked glycosylation 9, alpha-1,2-mannosyltransferase homolog (S. cerevisiae) |
| [9451](http://www.ncbi.nlm.nih.gov/gene/9451) | EIF2AK3 | eukaryotic translation initiation factor 2-alpha kinase 3 |
| [100302743](http://www.ncbi.nlm.nih.gov/gene/100302743) | SNORA80B | small nucleolar RNA, H/ACA box 80B |
| [3183](http://www.ncbi.nlm.nih.gov/gene/3183) | HNRNPC | heterogeneous nuclear ribonucleoprotein C (C1/C2) |
| [5411](http://www.ncbi.nlm.nih.gov/gene/5411) | PNN | pinin, desmosome associated protein |
| [7159](http://www.ncbi.nlm.nih.gov/gene/7159) | TP53BP2 | tumor protein p53 binding protein, 2 |
| [8775](http://www.ncbi.nlm.nih.gov/gene/8775) | NAPA | N-ethylmaleimide-sensitive factor attachment protein, alpha |
| [8178](http://www.ncbi.nlm.nih.gov/gene/8178) | ELL | elongation factor RNA polymerase II |
| [4601](http://www.ncbi.nlm.nih.gov/gene/4601) | MXI1 | MAX interactor 1 |
| [163479](http://www.ncbi.nlm.nih.gov/gene/163479) | FNDC7 | fibronectin type III domain containing 7 |
| [26785](http://www.ncbi.nlm.nih.gov/gene/26785) | SNORD63 | small nucleolar RNA, C/D box 63 |
| [677811](http://www.ncbi.nlm.nih.gov/gene/677811) | SNORA28 | small nucleolar RNA, H/ACA box 28 |
| [1506](http://www.ncbi.nlm.nih.gov/gene/1506) | CTRL | chymotrypsin-like |
| [5876](http://www.ncbi.nlm.nih.gov/gene/5876) | RABGGTB | Rab geranylgeranyltransferase, beta subunit |
| [90121](http://www.ncbi.nlm.nih.gov/gene/90121) | TSR2 | TSR2, 20S rRNA accumulation, homolog (S. cerevisiae) |
| [6811](http://www.ncbi.nlm.nih.gov/gene/6811) | STX5 | syntaxin 5 |
| [6498](http://www.ncbi.nlm.nih.gov/gene/6498) | SKIL | SKI-like oncogene |
| [55647](http://www.ncbi.nlm.nih.gov/gene/55647) | RAB20 | RAB20, member RAS oncogene family |
| [729966](http://www.ncbi.nlm.nih.gov/gene/729966) | LOC729966 | uncharacterized LOC729966 |
| [22863](http://www.ncbi.nlm.nih.gov/gene/22863) | ATG14 | autophagy related 14 |
| [9643](http://www.ncbi.nlm.nih.gov/gene/9643) | MORF4L2 | mortality factor 4 like 2 |
| [10693](http://www.ncbi.nlm.nih.gov/gene/10693) | CCT6B | chaperonin containing TCP1, subunit 6B (zeta 2) |
| [51582](http://www.ncbi.nlm.nih.gov/gene/51582) | AZIN1 | antizyme inhibitor 1 |
| [56985](http://www.ncbi.nlm.nih.gov/gene/56985) | ADPRM | ADP-ribose/CDP-alcohol diphosphatase, manganese-dependent |
| [57592](http://www.ncbi.nlm.nih.gov/gene/57592) | ZNF687 | zinc finger protein 687 |
| [51654](http://www.ncbi.nlm.nih.gov/gene/51654) | CDK5RAP1 | CDK5 regulatory subunit associated protein 1 |
| [199675](http://www.ncbi.nlm.nih.gov/gene/199675) | C19orf59 | chromosome 19 open reading frame 59 |
| [100129196](http://www.ncbi.nlm.nih.gov/gene/100129196) | MATN1-AS1 | MATN1 antisense RNA 1 |
| [158160](http://www.ncbi.nlm.nih.gov/gene/158160) | HSD17B7P2 | hydroxysteroid (17-beta) dehydrogenase 7 pseudogene 2 |
| [6844](http://www.ncbi.nlm.nih.gov/gene/6844) | VAMP2 | vesicle-associated membrane protein 2 (synaptobrevin 2) |
| [26959](http://www.ncbi.nlm.nih.gov/gene/26959) | HBP1 | HMG-box transcription factor 1 |
| [941](http://www.ncbi.nlm.nih.gov/gene/941) | CD80 | CD80 molecule |
| [8365](http://www.ncbi.nlm.nih.gov/gene/8365) | HIST1H4H | histone cluster 1, H4h |
| [3554](http://www.ncbi.nlm.nih.gov/gene/3554) | IL1R1 | interleukin 1 receptor, type I |
| [1022](http://www.ncbi.nlm.nih.gov/gene/1022) | CDK7 | cyclin-dependent kinase 7 |
| [677777](http://www.ncbi.nlm.nih.gov/gene/677777) | SCARNA12 | small Cajal body-specific RNA 12 |
| [130074](http://www.ncbi.nlm.nih.gov/gene/130074) | FAM168B | family with sequence similarity 168, member B |
| [29950](http://www.ncbi.nlm.nih.gov/gene/29950) | SERTAD1 | SERTA domain containing 1 |
| [7458](http://www.ncbi.nlm.nih.gov/gene/7458) | EIF4H | eukaryotic translation initiation factor 4H |
| [4282](http://www.ncbi.nlm.nih.gov/gene/4282) | MIF | macrophage migration inhibitory factor (glycosylation-inhibiting factor) |
| [3309](http://www.ncbi.nlm.nih.gov/gene/3309) | HSPA5 | heat shock 70kDa protein 5 (glucose-regulated protein, 78kDa) |
| [51729](http://www.ncbi.nlm.nih.gov/gene/51729) | WBP11 | WW domain binding protein 11 |
| [10211](http://www.ncbi.nlm.nih.gov/gene/10211) | FLOT1 | flotillin 1 |
| [7386](http://www.ncbi.nlm.nih.gov/gene/7386) | UQCRFS1 | ubiquinol-cytochrome c reductase, Rieske iron-sulfur polypeptide 1 |
| [677793](http://www.ncbi.nlm.nih.gov/gene/677793) | SNORA2A | small nucleolar RNA, H/ACA box 2A |
| [881](http://www.ncbi.nlm.nih.gov/gene/881) | CCIN | calicin |
| [26797](http://www.ncbi.nlm.nih.gov/gene/26797) | SNORD52 | small nucleolar RNA, C/D box 52 |
| [677839](http://www.ncbi.nlm.nih.gov/gene/677839) | SNORA71C | small nucleolar RNA, H/ACA box 71C |
| [8013](http://www.ncbi.nlm.nih.gov/gene/8013) | NR4A3 | nuclear receptor subfamily 4, group A, member 3 |
| [3949](http://www.ncbi.nlm.nih.gov/gene/3949) | LDLR | low density lipoprotein receptor |
| [153020](http://www.ncbi.nlm.nih.gov/gene/153020) | RASGEF1B | RasGEF domain family, member 1B |
| [23588](http://www.ncbi.nlm.nih.gov/gene/23588) | KLHDC2 | kelch domain containing 2 |
| [51465](http://www.ncbi.nlm.nih.gov/gene/51465) | UBE2J1 | ubiquitin-conjugating enzyme E2, J1 |
| [201283](http://www.ncbi.nlm.nih.gov/gene/201283) | AMZ2P1 | archaelysin family metallopeptidase 2 pseudogene 1 |
| [4000](http://www.ncbi.nlm.nih.gov/gene/4000) | LMNA | lamin A/C |
| [26816](http://www.ncbi.nlm.nih.gov/gene/26816) | SNORD35A | small nucleolar RNA, C/D box 35A |
| [4790](http://www.ncbi.nlm.nih.gov/gene/4790) | NFKB1 | nuclear factor of kappa light polypeptide gene enhancer in B-cells 1 |
| [2810](http://www.ncbi.nlm.nih.gov/gene/2810) | SFN | stratifin |
| [6322](http://www.ncbi.nlm.nih.gov/gene/6322) | SCML1 | sex comb on midleg-like 1 (Drosophila) |
| [6139](http://www.ncbi.nlm.nih.gov/gene/6139) | RPL17 | ribosomal protein L17 |
| [55186](http://www.ncbi.nlm.nih.gov/gene/55186) | SLC25A36 | solute carrier family 25 (pyrimidine nucleotide carrier ), member 36 |
| [65117](http://www.ncbi.nlm.nih.gov/gene/65117) | RSRC2 | arginine/serine-rich coiled-coil 2 |
| [100873755](http://www.ncbi.nlm.nih.gov/gene/100873755) | RNU6-23 | RNA, U6 small nuclear 23 |
| [28670](http://www.ncbi.nlm.nih.gov/gene/28670) | TRAV13-2 | T cell receptor alpha variable 13-2 |
| [23011](http://www.ncbi.nlm.nih.gov/gene/23011) | RAB21 | RAB21, member RAS oncogene family |
| [90427](http://www.ncbi.nlm.nih.gov/gene/90427) | BMF | Bcl2 modifying factor |
| [7158](http://www.ncbi.nlm.nih.gov/gene/7158) | TP53BP1 | tumor protein p53 binding protein 1 |
| [677825](http://www.ncbi.nlm.nih.gov/gene/677825) | SNORA44 | small nucleolar RNA, H/ACA box 44 |
| [81873](http://www.ncbi.nlm.nih.gov/gene/81873) | ARPC5L | actin related protein 2/3 complex, subunit 5-like |
| [83871](http://www.ncbi.nlm.nih.gov/gene/83871) | RAB34 | RAB34, member RAS oncogene family |
| [10250](http://www.ncbi.nlm.nih.gov/gene/10250) | SRRM1 | serine/arginine repetitive matrix 1 |
| [27291](http://www.ncbi.nlm.nih.gov/gene/27291) | R3HCC1L | R3H domain and coiled-coil containing 1-like |
| [92856](http://www.ncbi.nlm.nih.gov/gene/92856) | IMP4 | IMP4, U3 small nucleolar ribonucleoprotein, homolog (yeast) |
| [5222](http://www.ncbi.nlm.nih.gov/gene/5222) | PGA5 | pepsinogen 5, group I (pepsinogen A) |
| [23764](http://www.ncbi.nlm.nih.gov/gene/23764) | MAFF | v-maf musculoaponeurotic fibrosarcoma oncogene homolog F (avian) |
| [121268](http://www.ncbi.nlm.nih.gov/gene/121268) | RHEBL1 | Ras homolog enriched in brain like 1 |
| [65986](http://www.ncbi.nlm.nih.gov/gene/65986) | ZBTB10 | zinc finger and BTB domain containing 10 |
| [6829](http://www.ncbi.nlm.nih.gov/gene/6829) | SUPT5H | suppressor of Ty 5 homolog (S. cerevisiae) |
| [4839](http://www.ncbi.nlm.nih.gov/gene/4839) | NOP2 | NOP2 nucleolar protein homolog (yeast) |
| [23479](http://www.ncbi.nlm.nih.gov/gene/23479) | ISCU | iron-sulfur cluster scaffold homolog (E. coli) |
| [128308](http://www.ncbi.nlm.nih.gov/gene/128308) | MRPL55 | mitochondrial ribosomal protein L55 |
| [26051](http://www.ncbi.nlm.nih.gov/gene/26051) | PPP1R16B | protein phosphatase 1, regulatory subunit 16B |
| [283518](http://www.ncbi.nlm.nih.gov/gene/283518) | KCNRG | potassium channel regulator |
| [9536](http://www.ncbi.nlm.nih.gov/gene/9536) | PTGES | prostaglandin E synthase |
| [26783](http://www.ncbi.nlm.nih.gov/gene/26783) | SNORA65 | small nucleolar RNA, H/ACA box 65 |
| [391192](http://www.ncbi.nlm.nih.gov/gene/391192) | OR2L3 | olfactory receptor, family 2, subfamily L, member 3 |
| [51276](http://www.ncbi.nlm.nih.gov/gene/51276) | ZNF571 | zinc finger protein 571 |
| [57335](http://www.ncbi.nlm.nih.gov/gene/57335) | ZNF286A | zinc finger protein 286A |
| [6990](http://www.ncbi.nlm.nih.gov/gene/6990) | DYNLT3 | dynein, light chain, Tctex-type 3 |
| [57862](http://www.ncbi.nlm.nih.gov/gene/57862) | ZNF410 | zinc finger protein 410 |
| [100506667](http://www.ncbi.nlm.nih.gov/gene/100506667) | LOC100506667 | uncharacterized LOC100506667 |
| [55108](http://www.ncbi.nlm.nih.gov/gene/55108) | BSDC1 | BSD domain containing 1 |
| [5861](http://www.ncbi.nlm.nih.gov/gene/5861) | RAB1A | RAB1A, member RAS oncogene family |
| [55370](http://www.ncbi.nlm.nih.gov/gene/55370) | PPP4R1L | protein phosphatase 4, regulatory subunit 1-like |
| [64786](http://www.ncbi.nlm.nih.gov/gene/64786) | TBC1D15 | TBC1 domain family, member 15 |
| [6920](http://www.ncbi.nlm.nih.gov/gene/6920) | TCEA3 | transcription elongation factor A (SII), 3 |
| [163049](http://www.ncbi.nlm.nih.gov/gene/163049) | ZNF791 | zinc finger protein 791 |
| [162989](http://www.ncbi.nlm.nih.gov/gene/162989) | DEDD2 | death effector domain containing 2 |
| [143689](http://www.ncbi.nlm.nih.gov/gene/143689) | PIWIL4 | piwi-like 4 (Drosophila) |
| [10212](http://www.ncbi.nlm.nih.gov/gene/10212) | DDX39A | DEAD (Asp-Glu-Ala-Asp) box polypeptide 39A |
| [7430](http://www.ncbi.nlm.nih.gov/gene/7430) | EZR | ezrin |
| [401466](http://www.ncbi.nlm.nih.gov/gene/401466) | C8orf59 | chromosome 8 open reading frame 59 |
| [94081](http://www.ncbi.nlm.nih.gov/gene/94081) | SFXN1 | sideroflexin 1 |
| [22932](http://www.ncbi.nlm.nih.gov/gene/22932) | POMZP3 | POM121 and ZP3 fusion |
| [3422](http://www.ncbi.nlm.nih.gov/gene/3422) | IDI1 | isopentenyl-diphosphate delta isomerase 1 |
| [80714](http://www.ncbi.nlm.nih.gov/gene/80714) | PBX4 | pre-B-cell leukemia homeobox 4 |
| [29093](http://www.ncbi.nlm.nih.gov/gene/29093) | MRPL22 | mitochondrial ribosomal protein L22 |
| [677819](http://www.ncbi.nlm.nih.gov/gene/677819) | SNORA37 | small nucleolar RNA, H/ACA box 37 |
| [51593](http://www.ncbi.nlm.nih.gov/gene/51593) | SRRT | serrate RNA effector molecule homolog (Arabidopsis) |
| [120103](http://www.ncbi.nlm.nih.gov/gene/120103) | SLC36A4 | solute carrier family 36 (proton/amino acid symporter), member 4 |
| [10286](http://www.ncbi.nlm.nih.gov/gene/10286) | BCAS2 | breast carcinoma amplified sequence 2 |
| [116937](http://www.ncbi.nlm.nih.gov/gene/116937) | SNORD83A | small nucleolar RNA, C/D box 83A |
| [8774](http://www.ncbi.nlm.nih.gov/gene/8774) | NAPG | N-ethylmaleimide-sensitive factor attachment protein, gamma |
| [3638](http://www.ncbi.nlm.nih.gov/gene/3638) | INSIG1 | insulin induced gene 1 |
| [30836](http://www.ncbi.nlm.nih.gov/gene/30836) | DNTTIP2 | deoxynucleotidyltransferase, terminal, interacting protein 2 |
| [25820](http://www.ncbi.nlm.nih.gov/gene/25820) | ARIH1 | ariadne homolog, ubiquitin-conjugating enzyme E2 binding protein, 1 (Drosophila) |
| [3622](http://www.ncbi.nlm.nih.gov/gene/3622) | ING2 | inhibitor of growth family, member 2 |
| [606500](http://www.ncbi.nlm.nih.gov/gene/606500) | SNORD68 | small nucleolar RNA, C/D box 68 |
| [6832](http://www.ncbi.nlm.nih.gov/gene/6832) | SUPV3L1 | suppressor of var1, 3-like 1 (S. cerevisiae) |
| [56143](http://www.ncbi.nlm.nih.gov/gene/56143) | PCDHA5 | protocadherin alpha 5 |
| [10124](http://www.ncbi.nlm.nih.gov/gene/10124) | ARL4A | ADP-ribosylation factor-like 4A |
| [79441](http://www.ncbi.nlm.nih.gov/gene/79441) | HAUS3 | HAUS augmin-like complex, subunit 3 |
| [10625](http://www.ncbi.nlm.nih.gov/gene/10625) | IVNS1ABP | influenza virus NS1A binding protein |
| [64645](http://www.ncbi.nlm.nih.gov/gene/64645) | HIAT1 | hippocampus abundant transcript 1 |
| [22853](http://www.ncbi.nlm.nih.gov/gene/22853) | LMTK2 | lemur tyrosine kinase 2 |
| [60468](http://www.ncbi.nlm.nih.gov/gene/60468) | BACH2 | BTB and CNC homology 1, basic leucine zipper transcription factor 2 |
| [6449](http://www.ncbi.nlm.nih.gov/gene/6449) | SGTA | small glutamine-rich tetratricopeptide repeat (TPR)-containing, alpha |
| [83667](http://www.ncbi.nlm.nih.gov/gene/83667) | SESN2 | sestrin 2 |
| [54858](http://www.ncbi.nlm.nih.gov/gene/54858) | PGPEP1 | pyroglutamyl-peptidase I |
| [9567](http://www.ncbi.nlm.nih.gov/gene/9567) | GTPBP1 | GTP binding protein 1 |
| [140901](http://www.ncbi.nlm.nih.gov/gene/140901) | STK35 | serine/threonine kinase 35 |
| [54575](http://www.ncbi.nlm.nih.gov/gene/54575) | UGT1A10 | UDP glucuronosyltransferase 1 family, polypeptide A10 |
| [441150](http://www.ncbi.nlm.nih.gov/gene/441150) | C6orf226 | chromosome 6 open reading frame 226 |
| [84971](http://www.ncbi.nlm.nih.gov/gene/84971) | ATG4D | autophagy related 4D, cysteine peptidase |
| [8350](http://www.ncbi.nlm.nih.gov/gene/8350) | HIST1H3A | histone cluster 1, H3a |
| [54800](http://www.ncbi.nlm.nih.gov/gene/54800) | KLHL24 | kelch-like 24 (Drosophila) |
| [26793](http://www.ncbi.nlm.nih.gov/gene/26793) | SNORD56 | small nucleolar RNA, C/D box 56 |
| [26049](http://www.ncbi.nlm.nih.gov/gene/26049) | FAM169A | family with sequence similarity 169, member A |
| [2114](http://www.ncbi.nlm.nih.gov/gene/2114) | ETS2 | v-ets erythroblastosis virus E26 oncogene homolog 2 (avian) |
| [29115](http://www.ncbi.nlm.nih.gov/gene/29115) | SAP30BP | SAP30 binding protein |
| [10179](http://www.ncbi.nlm.nih.gov/gene/10179) | RBM7 | RNA binding motif protein 7 |
| [9782](http://www.ncbi.nlm.nih.gov/gene/9782) | MATR3 | matrin 3 |
| [9985](http://www.ncbi.nlm.nih.gov/gene/9985) | REC8 | REC8 homolog (yeast) |
| [2804](http://www.ncbi.nlm.nih.gov/gene/2804) | GOLGB1 | golgin B1 |
| [200916](http://www.ncbi.nlm.nih.gov/gene/200916) | RPL22L1 | ribosomal protein L22-like 1 |
| [26787](http://www.ncbi.nlm.nih.gov/gene/26787) | SNORD61 | small nucleolar RNA, C/D box 61 |
| [9975](http://www.ncbi.nlm.nih.gov/gene/9975) | NR1D2 | nuclear receptor subfamily 1, group D, member 2 |
| [9589](http://www.ncbi.nlm.nih.gov/gene/9589) | WTAP | Wilms tumor 1 associated protein |
| [415116](http://www.ncbi.nlm.nih.gov/gene/415116) | PIM3 | pim-3 oncogene |
| [10289](http://www.ncbi.nlm.nih.gov/gene/10289) | EIF1B | eukaryotic translation initiation factor 1B |
| [6884](http://www.ncbi.nlm.nih.gov/gene/6884) | TAF13 | TAF13 RNA polymerase II, TATA box binding protein (TBP)-associated factor, 18kDa |
| [27314](http://www.ncbi.nlm.nih.gov/gene/27314) | RAB30 | RAB30, member RAS oncogene family |
| [2119](http://www.ncbi.nlm.nih.gov/gene/2119) | ETV5 | ets variant 5 |
| [2298](http://www.ncbi.nlm.nih.gov/gene/2298) | FOXD4 | forkhead box D4 |
| [23541](http://www.ncbi.nlm.nih.gov/gene/23541) | SEC14L2 | SEC14-like 2 (S. cerevisiae) |
| [282617](http://www.ncbi.nlm.nih.gov/gene/282617) | IL28B | interleukin 28B (interferon, lambda 3) |
| [6615](http://www.ncbi.nlm.nih.gov/gene/6615) | SNAI1 | snail homolog 1 (Drosophila) |
| [10978](http://www.ncbi.nlm.nih.gov/gene/10978) | CLP1 | CLP1, cleavage and polyadenylation factor I subunit, homolog (S. cerevisiae) |
| [4209](http://www.ncbi.nlm.nih.gov/gene/4209) | MEF2D | myocyte enhancer factor 2D |
| [57600](http://www.ncbi.nlm.nih.gov/gene/57600) | FNIP2 | folliculin interacting protein 2 |
| [9318](http://www.ncbi.nlm.nih.gov/gene/9318) | COPS2 | COP9 constitutive photomorphogenic homolog subunit 2 (Arabidopsis) |
| [29896](http://www.ncbi.nlm.nih.gov/gene/29896) | TRA2A | transformer 2 alpha homolog (Drosophila) |
| [146456](http://www.ncbi.nlm.nih.gov/gene/146456) | TMED6 | transmembrane emp24 protein transport domain containing 6 |
| [100132341](http://www.ncbi.nlm.nih.gov/gene/100132341) | KIAA0664L3 | KIAA0664-like 3 |
| [3024](http://www.ncbi.nlm.nih.gov/gene/3024) | HIST1H1A | histone cluster 1, H1a |
| [56900](http://www.ncbi.nlm.nih.gov/gene/56900) | TMEM167B | transmembrane protein 167B |
| [51608](http://www.ncbi.nlm.nih.gov/gene/51608) | GET4 | golgi to ER traffic protein 4 homolog (S. cerevisiae) |
| [348094](http://www.ncbi.nlm.nih.gov/gene/348094) | ANKDD1A | ankyrin repeat and death domain containing 1A |
| [55909](http://www.ncbi.nlm.nih.gov/gene/55909) | BIN3 | bridging integrator 3 |
| [10622](http://www.ncbi.nlm.nih.gov/gene/10622) | POLR3G | polymerase (RNA) III (DNA directed) polypeptide G (32kD) |
| [80758](http://www.ncbi.nlm.nih.gov/gene/80758) | PRR7 | proline rich 7 (synaptic) |
| [80273](http://www.ncbi.nlm.nih.gov/gene/80273) | GRPEL1 | GrpE-like 1, mitochondrial (E. coli) |
| [57192](http://www.ncbi.nlm.nih.gov/gene/57192) | MCOLN1 | mucolipin 1 |
| [26765](http://www.ncbi.nlm.nih.gov/gene/26765) | SNORD12C | small nucleolar RNA, C/D box 12C |
| [55142](http://www.ncbi.nlm.nih.gov/gene/55142) | HAUS2 | HAUS augmin-like complex, subunit 2 |
| [84707](http://www.ncbi.nlm.nih.gov/gene/84707) | BEX2 | brain expressed X-linked 2 |
| [6309](http://www.ncbi.nlm.nih.gov/gene/6309) | SC5DL | sterol-C5-desaturase (ERG3 delta-5-desaturase homolog, S. cerevisiae)-like |
| [359948](http://www.ncbi.nlm.nih.gov/gene/359948) | IRF2BP2 | interferon regulatory factor 2 binding protein 2 |
| [8565](http://www.ncbi.nlm.nih.gov/gene/8565) | YARS | tyrosyl-tRNA synthetase |
| [23753](http://www.ncbi.nlm.nih.gov/gene/23753) | SDF2L1 | stromal cell-derived factor 2-like 1 |
| [5718](http://www.ncbi.nlm.nih.gov/gene/5718) | PSMD12 | proteasome (prosome, macropain) 26S subunit, non-ATPase, 12 |
| [8507](http://www.ncbi.nlm.nih.gov/gene/8507) | ENC1 | ectodermal-neural cortex 1 (with BTB-like domain) |
| [144195](http://www.ncbi.nlm.nih.gov/gene/144195) | SLC2A14 | solute carrier family 2 (facilitated glucose transporter), member 14 |
| [26806](http://www.ncbi.nlm.nih.gov/gene/26806) | SNORD44 | small nucleolar RNA, C/D box 44 |
| [57226](http://www.ncbi.nlm.nih.gov/gene/57226) | LYRM2 | LYR motif containing 2 |
| [1553](http://www.ncbi.nlm.nih.gov/gene/1553) | CYP2A13 | cytochrome P450, family 2, subfamily A, polypeptide 13 |
| [58516](http://www.ncbi.nlm.nih.gov/gene/58516) | FAM60A | family with sequence similarity 60, member A |
| [127544](http://www.ncbi.nlm.nih.gov/gene/127544) | RNF19B | ring finger protein 19B |
| [164](http://www.ncbi.nlm.nih.gov/gene/164) | AP1G1 | adaptor-related protein complex 1, gamma 1 subunit |
| [101059928](http://www.ncbi.nlm.nih.gov/gene/101059928) |  |  |
| [79893](http://www.ncbi.nlm.nih.gov/gene/79893) | GGNBP2 | gametogenetin binding protein 2 |
| [27000](http://www.ncbi.nlm.nih.gov/gene/27000) | DNAJC2 | DnaJ (Hsp40) homolog, subfamily C, member 2 |
| [692158](http://www.ncbi.nlm.nih.gov/gene/692158) | SNORA57 | small nucleolar RNA, H/ACA box 57 |
| [50640](http://www.ncbi.nlm.nih.gov/gene/50640) | PNPLA8 | patatin-like phospholipase domain containing 8 |
| [373856](http://www.ncbi.nlm.nih.gov/gene/373856) | USP41 | ubiquitin specific peptidase 41 |
| [100528018](http://www.ncbi.nlm.nih.gov/gene/100528018) | ARL2-SNX15 | ARL2-SNX15 readthrough |
| [51562](http://www.ncbi.nlm.nih.gov/gene/51562) | MBIP | MAP3K12 binding inhibitory protein 1 |
| [902](http://www.ncbi.nlm.nih.gov/gene/902) | CCNH | cyclin H |
| [9846](http://www.ncbi.nlm.nih.gov/gene/9846) | GAB2 | GRB2-associated binding protein 2 |
| [662](http://www.ncbi.nlm.nih.gov/gene/662) | BNIP1 | BCL2/adenovirus E1B 19kDa interacting protein 1 |
| [29774](http://www.ncbi.nlm.nih.gov/gene/29774) | POM121L9P | POM121 transmembrane nucleoporin-like 9, pseudogene |
| [10804](http://www.ncbi.nlm.nih.gov/gene/10804) | GJB6 | gap junction protein, beta 6, 30kDa |
| [53371](http://www.ncbi.nlm.nih.gov/gene/53371) | NUP54 | nucleoporin 54kDa |
| [402055](http://www.ncbi.nlm.nih.gov/gene/402055) | SRRD | SRR1 domain containing |
| [51759](http://www.ncbi.nlm.nih.gov/gene/51759) | C9orf78 | chromosome 9 open reading frame 78 |
| [692090](http://www.ncbi.nlm.nih.gov/gene/692090) | SNORD59B | small nucleolar RNA, C/D box 59B |
| [10569](http://www.ncbi.nlm.nih.gov/gene/10569) | SLU7 | SLU7 splicing factor homolog (S. cerevisiae) |
| [5341](http://www.ncbi.nlm.nih.gov/gene/5341) | PLEK | pleckstrin |
| [101059922](http://www.ncbi.nlm.nih.gov/gene/101059922) |  |  |
| [10209](http://www.ncbi.nlm.nih.gov/gene/10209) | EIF1 | eukaryotic translation initiation factor 1 |
| [163154](http://www.ncbi.nlm.nih.gov/gene/163154) | PRR22 | proline rich 22 |
| [10208](http://www.ncbi.nlm.nih.gov/gene/10208) | USPL1 | ubiquitin specific peptidase like 1 |
| [1454](http://www.ncbi.nlm.nih.gov/gene/1454) | CSNK1E | casein kinase 1, epsilon |
| [5900](http://www.ncbi.nlm.nih.gov/gene/5900) | RALGDS | ral guanine nucleotide dissociation stimulator |
| [221756](http://www.ncbi.nlm.nih.gov/gene/221756) | MGC39372 | serpin peptidase inhibitor, clade B (ovalbumin), member 9 pseudogene |
| [6830](http://www.ncbi.nlm.nih.gov/gene/6830) | SUPT6H | suppressor of Ty 6 homolog (S. cerevisiae) |
| [386685](http://www.ncbi.nlm.nih.gov/gene/386685) | KRTAP10-12 | keratin associated protein 10-12 |
| [340591](http://www.ncbi.nlm.nih.gov/gene/340591) | CA5BP1 | carbonic anhydrase VB pseudogene 1 |
| [10488](http://www.ncbi.nlm.nih.gov/gene/10488) | CREB3 | cAMP responsive element binding protein 3 |
| [2935](http://www.ncbi.nlm.nih.gov/gene/2935) | GSPT1 | G1 to S phase transition 1 |
| [10440](http://www.ncbi.nlm.nih.gov/gene/10440) | TIMM17A | translocase of inner mitochondrial membrane 17 homolog A (yeast) |
| [283694](http://www.ncbi.nlm.nih.gov/gene/283694) | OR4N4 | olfactory receptor, family 4, subfamily N, member 4 |
| [8204](http://www.ncbi.nlm.nih.gov/gene/8204) | NRIP1 | nuclear receptor interacting protein 1 |
| [9804](http://www.ncbi.nlm.nih.gov/gene/9804) | TOMM20 | translocase of outer mitochondrial membrane 20 homolog (yeast) |
| [10342](http://www.ncbi.nlm.nih.gov/gene/10342) | TFG | TRK-fused gene |
| [387748](http://www.ncbi.nlm.nih.gov/gene/387748) | OR56B1 | olfactory receptor, family 56, subfamily B, member 1 |
| [10933](http://www.ncbi.nlm.nih.gov/gene/10933) | MORF4L1 | mortality factor 4 like 1 |
| [114548](http://www.ncbi.nlm.nih.gov/gene/114548) | NLRP3 | NLR family, pyrin domain containing 3 |
| [28666](http://www.ncbi.nlm.nih.gov/gene/28666) | TRAV17 | T cell receptor alpha variable 17 |
| [26024](http://www.ncbi.nlm.nih.gov/gene/26024) | PTCD1 | pentatricopeptide repeat domain 1 |
| [2146](http://www.ncbi.nlm.nih.gov/gene/2146) | EZH2 | enhancer of zeste homolog 2 (Drosophila) |
| [6421](http://www.ncbi.nlm.nih.gov/gene/6421) | SFPQ | splicing factor proline/glutamine-rich |
| [619562](http://www.ncbi.nlm.nih.gov/gene/619562) | SNORA3 | small nucleolar RNA, H/ACA box 3 |
| [113791](http://www.ncbi.nlm.nih.gov/gene/113791) | PIK3IP1 | phosphoinositide-3-kinase interacting protein 1 |
| [10189](http://www.ncbi.nlm.nih.gov/gene/10189) | ALYREF | Aly/REF export factor |
| [54509](http://www.ncbi.nlm.nih.gov/gene/54509) | RHOF | ras homolog family member F (in filopodia) |
| [6631](http://www.ncbi.nlm.nih.gov/gene/6631) | SNRPC | small nuclear ribonucleoprotein polypeptide C |
| [6432](http://www.ncbi.nlm.nih.gov/gene/6432) | SRSF7 | serine/arginine-rich splicing factor 7 |
| [1390](http://www.ncbi.nlm.nih.gov/gene/1390) | CREM | cAMP responsive element modulator |
| [55432](http://www.ncbi.nlm.nih.gov/gene/55432) | YOD1 | YOD1 OTU deubiquinating enzyme 1 homolog (S. cerevisiae) |
| [400870](http://www.ncbi.nlm.nih.gov/gene/400870) | FLJ41733 | FLJ41733 protein |
| [5423](http://www.ncbi.nlm.nih.gov/gene/5423) | POLB | polymerase (DNA directed), beta |
| [26774](http://www.ncbi.nlm.nih.gov/gene/26774) | SNORD80 | small nucleolar RNA, C/D box 80 |
| [143684](http://www.ncbi.nlm.nih.gov/gene/143684) | FAM76B | family with sequence similarity 76, member B |
| [26088](http://www.ncbi.nlm.nih.gov/gene/26088) | GGA1 | golgi-associated, gamma adaptin ear containing, ARF binding protein 1 |
| [285966](http://www.ncbi.nlm.nih.gov/gene/285966) | FAM115C | family with sequence similarity 115, member C |
| [9541](http://www.ncbi.nlm.nih.gov/gene/9541) | CIR1 | corepressor interacting with RBPJ, 1 |
| [10482](http://www.ncbi.nlm.nih.gov/gene/10482) | NXF1 | nuclear RNA export factor 1 |
| [4809](http://www.ncbi.nlm.nih.gov/gene/4809) | NHP2L1 | NHP2 non-histone chromosome protein 2-like 1 (S. cerevisiae) |
| [388796](http://www.ncbi.nlm.nih.gov/gene/388796) | LOC388796 | uncharacterized LOC388796 |
| [83443](http://www.ncbi.nlm.nih.gov/gene/83443) | SF3B5 | splicing factor 3b, subunit 5, 10kDa |
| [56261](http://www.ncbi.nlm.nih.gov/gene/56261) | GPCPD1 | glycerophosphocholine phosphodiesterase GDE1 homolog (S. cerevisiae) |
| [5290](http://www.ncbi.nlm.nih.gov/gene/5290) | PIK3CA | phosphatidylinositol-4,5-bisphosphate 3-kinase, catalytic subunit alpha |
| [3563](http://www.ncbi.nlm.nih.gov/gene/3563) | IL3RA | interleukin 3 receptor, alpha (low affinity) |
| [7307](http://www.ncbi.nlm.nih.gov/gene/7307) | U2AF1 | U2 small nuclear RNA auxiliary factor 1 |
| [55773](http://www.ncbi.nlm.nih.gov/gene/55773) | TBC1D23 | TBC1 domain family, member 23 |
| [7134](http://www.ncbi.nlm.nih.gov/gene/7134) | TNNC1 | troponin C type 1 (slow) |
| [4291](http://www.ncbi.nlm.nih.gov/gene/4291) | MLF1 | myeloid leukemia factor 1 |
| [9572](http://www.ncbi.nlm.nih.gov/gene/9572) | NR1D1 | nuclear receptor subfamily 1, group D, member 1 |
| [54840](http://www.ncbi.nlm.nih.gov/gene/54840) | APTX | aprataxin |
| [57157](http://www.ncbi.nlm.nih.gov/gene/57157) | PHTF2 | putative homeodomain transcription factor 2 |
| [8754](http://www.ncbi.nlm.nih.gov/gene/8754) | ADAM9 | ADAM metallopeptidase domain 9 |
| [51042](http://www.ncbi.nlm.nih.gov/gene/51042) | ZNF593 | zinc finger protein 593 |
| [527](http://www.ncbi.nlm.nih.gov/gene/527) | ATP6V0C | ATPase, H+ transporting, lysosomal 16kDa, V0 subunit c |
| [9232](http://www.ncbi.nlm.nih.gov/gene/9232) | PTTG1 | pituitary tumor-transforming 1 |
| [5427](http://www.ncbi.nlm.nih.gov/gene/5427) | POLE2 | polymerase (DNA directed), epsilon 2, accessory subunit |
| [1847](http://www.ncbi.nlm.nih.gov/gene/1847) | DUSP5 | dual specificity phosphatase 5 |
| [7316](http://www.ncbi.nlm.nih.gov/gene/7316) | UBC | ubiquitin C |
| [3732](http://www.ncbi.nlm.nih.gov/gene/3732) | CD82 | CD82 molecule |
| [90288](http://www.ncbi.nlm.nih.gov/gene/90288) | EFCAB12 | EF-hand calcium binding domain 12 |
| [9819](http://www.ncbi.nlm.nih.gov/gene/9819) | TSC22D2 | TSC22 domain family, member 2 |
| [1153](http://www.ncbi.nlm.nih.gov/gene/1153) | CIRBP | cold inducible RNA binding protein |
| [100130967](http://www.ncbi.nlm.nih.gov/gene/100130967) | C6orf99 | chromosome 6 open reading frame 99 |
| [23576](http://www.ncbi.nlm.nih.gov/gene/23576) | DDAH1 | dimethylarginine dimethylaminohydrolase 1 |
| [8315](http://www.ncbi.nlm.nih.gov/gene/8315) | BRAP | BRCA1 associated protein |
| [84268](http://www.ncbi.nlm.nih.gov/gene/84268) | RPAIN | RPA interacting protein |
| [51193](http://www.ncbi.nlm.nih.gov/gene/51193) | ZNF639 | zinc finger protein 639 |
| [10767](http://www.ncbi.nlm.nih.gov/gene/10767) | HBS1L | HBS1-like (S. cerevisiae) |
| [100287569](http://www.ncbi.nlm.nih.gov/gene/100287569) | LINC00173 | long intergenic non-protein coding RNA 173 |
| [2787](http://www.ncbi.nlm.nih.gov/gene/2787) | GNG5 | guanine nucleotide binding protein (G protein), gamma 5 |
| [9363](http://www.ncbi.nlm.nih.gov/gene/9363) | RAB33A | RAB33A, member RAS oncogene family |
| [80700](http://www.ncbi.nlm.nih.gov/gene/80700) | UBXN6 | UBX domain protein 6 |
| [9774](http://www.ncbi.nlm.nih.gov/gene/9774) | BCLAF1 | BCL2-associated transcription factor 1 |
| [7403](http://www.ncbi.nlm.nih.gov/gene/7403) | KDM6A | lysine (K)-specific demethylase 6A |
| [374977](http://www.ncbi.nlm.nih.gov/gene/374977) | HEATR8 | HEAT repeat containing 8 |
| [51275](http://www.ncbi.nlm.nih.gov/gene/51275) | MAPKAPK5-AS1 | MAPKAPK5 antisense RNA 1 |
| [5903](http://www.ncbi.nlm.nih.gov/gene/5903) | RANBP2 | RAN binding protein 2 |
| [9086](http://www.ncbi.nlm.nih.gov/gene/9086) | EIF1AY | eukaryotic translation initiation factor 1A, Y-linked |
| [56624](http://www.ncbi.nlm.nih.gov/gene/56624) | ASAH2 | N-acylsphingosine amidohydrolase (non-lysosomal ceramidase) 2 |
| [54413](http://www.ncbi.nlm.nih.gov/gene/54413) | NLGN3 | neuroligin 3 |
| [2197](http://www.ncbi.nlm.nih.gov/gene/2197) | FAU | Finkel-Biskis-Reilly murine sarcoma virus (FBR-MuSV) ubiquitously expressed |
| [1054](http://www.ncbi.nlm.nih.gov/gene/1054) | CEBPG | CCAAT/enhancer binding protein (C/EBP), gamma |
| [100130331](http://www.ncbi.nlm.nih.gov/gene/100130331) | LOC100130331 | POTE ankyrin domain family, member F pseudogene |
| [3191](http://www.ncbi.nlm.nih.gov/gene/3191) | HNRNPL | heterogeneous nuclear ribonucleoprotein L |
| [9322](http://www.ncbi.nlm.nih.gov/gene/9322) | TRIP10 | thyroid hormone receptor interactor 10 |
| [391712](http://www.ncbi.nlm.nih.gov/gene/391712) | TRIM61 | tripartite motif containing 61 |
| [22938](http://www.ncbi.nlm.nih.gov/gene/22938) | SNW1 | SNW domain containing 1 |
| [267010](http://www.ncbi.nlm.nih.gov/gene/267010) | RNU12 | RNA, U12 small nuclear |
| [10758](http://www.ncbi.nlm.nih.gov/gene/10758) | TRAF3IP2 | TRAF3 interacting protein 2 |
| [729830](http://www.ncbi.nlm.nih.gov/gene/729830) | FAM160A1 | family with sequence similarity 160, member A1 |
| [8358](http://www.ncbi.nlm.nih.gov/gene/8358) | HIST1H3B | histone cluster 1, H3b |
| [25873](http://www.ncbi.nlm.nih.gov/gene/25873) | RPL36 | ribosomal protein L36 |
| [1060](http://www.ncbi.nlm.nih.gov/gene/1060) | CENPC1 | centromere protein C 1 |
| [5016](http://www.ncbi.nlm.nih.gov/gene/5016) | OVGP1 | oviductal glycoprotein 1, 120kDa |
| [11143](http://www.ncbi.nlm.nih.gov/gene/11143) | KAT7 | K(lysine) acetyltransferase 7 |
| [5393](http://www.ncbi.nlm.nih.gov/gene/5393) | EXOSC9 | exosome component 9 |
| [9775](http://www.ncbi.nlm.nih.gov/gene/9775) | EIF4A3 | eukaryotic translation initiation factor 4A3 |
| [55544](http://www.ncbi.nlm.nih.gov/gene/55544) | RBM38 | RNA binding motif protein 38 |
| [100287932](http://www.ncbi.nlm.nih.gov/gene/100287932) | TIMM23 | translocase of inner mitochondrial membrane 23 homolog (yeast) |
| [79665](http://www.ncbi.nlm.nih.gov/gene/79665) | DHX40 | DEAH (Asp-Glu-Ala-His) box polypeptide 40 |
| [23268](http://www.ncbi.nlm.nih.gov/gene/23268) | DNMBP | dynamin binding protein |
| [55837](http://www.ncbi.nlm.nih.gov/gene/55837) | EAPP | E2F-associated phosphoprotein |
| [283970](http://www.ncbi.nlm.nih.gov/gene/283970) | PDXDC2P | pyridoxal-dependent decarboxylase domain containing 2, pseudogene |
| [221830](http://www.ncbi.nlm.nih.gov/gene/221830) | TWISTNB | TWIST neighbor |
| [4068](http://www.ncbi.nlm.nih.gov/gene/4068) | SH2D1A | SH2 domain containing 1A |
| [28688](http://www.ncbi.nlm.nih.gov/gene/28688) | TRAV5 | T cell receptor alpha variable 5 |
| [8969](http://www.ncbi.nlm.nih.gov/gene/8969) | HIST1H2AG | histone cluster 1, H2ag |
| [10671](http://www.ncbi.nlm.nih.gov/gene/10671) | DCTN6 | dynactin 6 |
| [389766](http://www.ncbi.nlm.nih.gov/gene/389766) | C9orf153 | chromosome 9 open reading frame 153 |
| [6513](http://www.ncbi.nlm.nih.gov/gene/6513) | SLC2A1 | solute carrier family 2 (facilitated glucose transporter), member 1 |
| [65996](http://www.ncbi.nlm.nih.gov/gene/65996) | MGC2752 | CENPB DNA-binding domains containing 1 pseudogene |
| [8343](http://www.ncbi.nlm.nih.gov/gene/8343) | HIST1H2BF | histone cluster 1, H2bf |
| [58155](http://www.ncbi.nlm.nih.gov/gene/58155) | PTBP2 | polypyrimidine tract binding protein 2 |
| [79753](http://www.ncbi.nlm.nih.gov/gene/79753) | SNIP1 | Smad nuclear interacting protein 1 |
| [90390](http://www.ncbi.nlm.nih.gov/gene/90390) | MED30 | mediator complex subunit 30 |
| [23275](http://www.ncbi.nlm.nih.gov/gene/23275) | POFUT2 | protein O-fucosyltransferase 2 |
| [140775](http://www.ncbi.nlm.nih.gov/gene/140775) | SMCR8 | Smith-Magenis syndrome chromosome region, candidate 8 |
| [3005](http://www.ncbi.nlm.nih.gov/gene/3005) | H1F0 | H1 histone family, member 0 |
| [51430](http://www.ncbi.nlm.nih.gov/gene/51430) | SUCO | SUN domain containing ossification factor |
| [56949](http://www.ncbi.nlm.nih.gov/gene/56949) | XAB2 | XPA binding protein 2 |
| [51710](http://www.ncbi.nlm.nih.gov/gene/51710) | ZNF44 | zinc finger protein 44 |
| [22850](http://www.ncbi.nlm.nih.gov/gene/22850) | ADNP2 | ADNP homeobox 2 |
| [58490](http://www.ncbi.nlm.nih.gov/gene/58490) | RPRD1B | regulation of nuclear pre-mRNA domain containing 1B |
| [482](http://www.ncbi.nlm.nih.gov/gene/482) | ATP1B2 | ATPase, Na+/K+ transporting, beta 2 polypeptide |
| [51061](http://www.ncbi.nlm.nih.gov/gene/51061) | TXNDC11 | thioredoxin domain containing 11 |
| [148423](http://www.ncbi.nlm.nih.gov/gene/148423) | C1orf52 | chromosome 1 open reading frame 52 |
| [64319](http://www.ncbi.nlm.nih.gov/gene/64319) | FBRS | fibrosin |
| [6192](http://www.ncbi.nlm.nih.gov/gene/6192) | RPS4Y1 | ribosomal protein S4, Y-linked 1 |
| [90411](http://www.ncbi.nlm.nih.gov/gene/90411) | MCFD2 | multiple coagulation factor deficiency 2 |
| [55915](http://www.ncbi.nlm.nih.gov/gene/55915) | LANCL2 | LanC lantibiotic synthetase component C-like 2 (bacterial) |
| [3398](http://www.ncbi.nlm.nih.gov/gene/3398) | ID2 | inhibitor of DNA binding 2, dominant negative helix-loop-helix protein |
| [254263](http://www.ncbi.nlm.nih.gov/gene/254263) | CNIH2 | cornichon homolog 2 (Drosophila) |
| [10713](http://www.ncbi.nlm.nih.gov/gene/10713) | USP39 | ubiquitin specific peptidase 39 |
| [54587](http://www.ncbi.nlm.nih.gov/gene/54587) | MXRA8 | matrix-remodelling associated 8 |
| [93643](http://www.ncbi.nlm.nih.gov/gene/93643) | TJAP1 | tight junction associated protein 1 (peripheral) |
| [5277](http://www.ncbi.nlm.nih.gov/gene/5277) | PIGA | phosphatidylinositol glycan anchor biosynthesis, class A |
| [11314](http://www.ncbi.nlm.nih.gov/gene/11314) | CD300A | CD300a molecule |
| [55041](http://www.ncbi.nlm.nih.gov/gene/55041) | PLEKHB2 | pleckstrin homology domain containing, family B (evectins) member 2 |
| [10105](http://www.ncbi.nlm.nih.gov/gene/10105) | PPIF | peptidylprolyl isomerase F |
| [1548](http://www.ncbi.nlm.nih.gov/gene/1548) | CYP2A6 | cytochrome P450, family 2, subfamily A, polypeptide 6 |
| [9275](http://www.ncbi.nlm.nih.gov/gene/9275) | BCL7B | B-cell CLL/lymphoma 7B |
| [25978](http://www.ncbi.nlm.nih.gov/gene/25978) | CHMP2B | charged multivesicular body protein 2B |
| [55254](http://www.ncbi.nlm.nih.gov/gene/55254) | TMEM39A | transmembrane protein 39A |
| [6652](http://www.ncbi.nlm.nih.gov/gene/6652) | SORD | sorbitol dehydrogenase |
| [84331](http://www.ncbi.nlm.nih.gov/gene/84331) | FAM195A | family with sequence similarity 195, member A |
| [144245](http://www.ncbi.nlm.nih.gov/gene/144245) | ALG10B | asparagine-linked glycosylation 10, alpha-1,2-glucosyltransferase homolog B (yeast) |
| [9094](http://www.ncbi.nlm.nih.gov/gene/9094) | UNC119 | unc-119 homolog (C. elegans) |
| [10572](http://www.ncbi.nlm.nih.gov/gene/10572) | SIVA1 | SIVA1, apoptosis-inducing factor |
| [91298](http://www.ncbi.nlm.nih.gov/gene/91298) | C12orf29 | chromosome 12 open reading frame 29 |
| [396](http://www.ncbi.nlm.nih.gov/gene/396) | ARHGDIA | Rho GDP dissociation inhibitor (GDI) alpha |
| [353137](http://www.ncbi.nlm.nih.gov/gene/353137) | LCE1F | late cornified envelope 1F |
| [1164](http://www.ncbi.nlm.nih.gov/gene/1164) | CKS2 | CDC28 protein kinase regulatory subunit 2 |
| [8227](http://www.ncbi.nlm.nih.gov/gene/8227) | AKAP17A | A kinase (PRKA) anchor protein 17A |
| [54468](http://www.ncbi.nlm.nih.gov/gene/54468) | MIOS | missing oocyte, meiosis regulator, homolog (Drosophila) |
| [84838](http://www.ncbi.nlm.nih.gov/gene/84838) | ZNF496 | zinc finger protein 496 |
| [7341](http://www.ncbi.nlm.nih.gov/gene/7341) | SUMO1 | SMT3 suppressor of mif two 3 homolog 1 (S. cerevisiae) |
| [2870](http://www.ncbi.nlm.nih.gov/gene/2870) | GRK6 | G protein-coupled receptor kinase 6 |
| [80219](http://www.ncbi.nlm.nih.gov/gene/80219) | COQ10B | coenzyme Q10 homolog B (S. cerevisiae) |
| [619569](http://www.ncbi.nlm.nih.gov/gene/619569) | SNORA41 | small nucleolar RNA, H/ACA box 41 |
| [7319](http://www.ncbi.nlm.nih.gov/gene/7319) | UBE2A | ubiquitin-conjugating enzyme E2A |
| [9261](http://www.ncbi.nlm.nih.gov/gene/9261) | MAPKAPK2 | mitogen-activated protein kinase-activated protein kinase 2 |
| [23484](http://www.ncbi.nlm.nih.gov/gene/23484) | LEPROTL1 | leptin receptor overlapping transcript-like 1 |
| [272](http://www.ncbi.nlm.nih.gov/gene/272) | AMPD3 | adenosine monophosphate deaminase 3 |
| [643664](http://www.ncbi.nlm.nih.gov/gene/643664) | SLC35G6 | solute carrier family 35, member G6 |
| [54870](http://www.ncbi.nlm.nih.gov/gene/54870) | QRICH1 | glutamine-rich 1 |
| [80331](http://www.ncbi.nlm.nih.gov/gene/80331) | DNAJC5 | DnaJ (Hsp40) homolog, subfamily C, member 5 |
| [7385](http://www.ncbi.nlm.nih.gov/gene/7385) | UQCRC2 | ubiquinol-cytochrome c reductase core protein II |
| [2874](http://www.ncbi.nlm.nih.gov/gene/2874) | GPS2 | G protein pathway suppressor 2 |
| [10252](http://www.ncbi.nlm.nih.gov/gene/10252) | SPRY1 | sprouty homolog 1, antagonist of FGF signaling (Drosophila) |
| [7572](http://www.ncbi.nlm.nih.gov/gene/7572) | ZNF24 | zinc finger protein 24 |
| [8658](http://www.ncbi.nlm.nih.gov/gene/8658) | TNKS | tankyrase, TRF1-interacting ankyrin-related ADP-ribose polymerase |
| [10270](http://www.ncbi.nlm.nih.gov/gene/10270) | AKAP8 | A kinase (PRKA) anchor protein 8 |
| [58515](http://www.ncbi.nlm.nih.gov/gene/58515) | SELK | selenoprotein K |
| [8346](http://www.ncbi.nlm.nih.gov/gene/8346) | HIST1H2BI | histone cluster 1, H2bi |
| [11103](http://www.ncbi.nlm.nih.gov/gene/11103) | KRR1 | KRR1, small subunit (SSU) processome component, homolog (yeast) |
| [1435](http://www.ncbi.nlm.nih.gov/gene/1435) | CSF1 | colony stimulating factor 1 (macrophage) |
| [84246](http://www.ncbi.nlm.nih.gov/gene/84246) | MED10 | mediator complex subunit 10 |
| [10253](http://www.ncbi.nlm.nih.gov/gene/10253) | SPRY2 | sprouty homolog 2 (Drosophila) |
| [8364](http://www.ncbi.nlm.nih.gov/gene/8364) | HIST1H4C | histone cluster 1, H4c |
| [9360](http://www.ncbi.nlm.nih.gov/gene/9360) | PPIG | peptidylprolyl isomerase G (cyclophilin G) |
| [26017](http://www.ncbi.nlm.nih.gov/gene/26017) | FAM32A | family with sequence similarity 32, member A |
| [55565](http://www.ncbi.nlm.nih.gov/gene/55565) | ZNF821 | zinc finger protein 821 |
| [1540](http://www.ncbi.nlm.nih.gov/gene/1540) | CYLD | cylindromatosis (turban tumor syndrome) |
| [84324](http://www.ncbi.nlm.nih.gov/gene/84324) | SARNP | SAP domain containing ribonucleoprotein |
| [26789](http://www.ncbi.nlm.nih.gov/gene/26789) | SNORD59A | small nucleolar RNA, C/D box 59A |
| [1032](http://www.ncbi.nlm.nih.gov/gene/1032) | CDKN2D | cyclin-dependent kinase inhibitor 2D (p19, inhibits CDK4) |
| [11213](http://www.ncbi.nlm.nih.gov/gene/11213) | IRAK3 | interleukin-1 receptor-associated kinase 3 |
| [26784](http://www.ncbi.nlm.nih.gov/gene/26784) | SNORA64 | small nucleolar RNA, H/ACA box 64 |
| [6675](http://www.ncbi.nlm.nih.gov/gene/6675) | UAP1 | UDP-N-acteylglucosamine pyrophosphorylase 1 |
| [124599](http://www.ncbi.nlm.nih.gov/gene/124599) | CD300LB | CD300 molecule-like family member b |
| [58191](http://www.ncbi.nlm.nih.gov/gene/58191) | CXCL16 | chemokine (C-X-C motif) ligand 16 |
| [29107](http://www.ncbi.nlm.nih.gov/gene/29107) | NXT1 | NTF2-like export factor 1 |
| [1039](http://www.ncbi.nlm.nih.gov/gene/1039) | CDR2 | cerebellar degeneration-related protein 2, 62kDa |
| [26099](http://www.ncbi.nlm.nih.gov/gene/26099) | SZRD1 | SUZ RNA binding domain containing 1 |
| [55805](http://www.ncbi.nlm.nih.gov/gene/55805) | LRP2BP | LRP2 binding protein |
| [8087](http://www.ncbi.nlm.nih.gov/gene/8087) | FXR1 | fragile X mental retardation, autosomal homolog 1 |
| [3157](http://www.ncbi.nlm.nih.gov/gene/3157) | HMGCS1 | 3-hydroxy-3-methylglutaryl-CoA synthase 1 (soluble) |
| [10802](http://www.ncbi.nlm.nih.gov/gene/10802) | SEC24A | SEC24 family, member A (S. cerevisiae) |
| [3008](http://www.ncbi.nlm.nih.gov/gene/3008) | HIST1H1E | histone cluster 1, H1e |
| [637](http://www.ncbi.nlm.nih.gov/gene/637) | BID | BH3 interacting domain death agonist |
| [5008](http://www.ncbi.nlm.nih.gov/gene/5008) | OSM | oncostatin M |
| [55969](http://www.ncbi.nlm.nih.gov/gene/55969) | C20orf24 | chromosome 20 open reading frame 24 |
| [5331](http://www.ncbi.nlm.nih.gov/gene/5331) | PLCB3 | phospholipase C, beta 3 (phosphatidylinositol-specific) |
| [200058](http://www.ncbi.nlm.nih.gov/gene/200058) | FLJ23867 | uncharacterized protein FLJ23867 |
| [8480](http://www.ncbi.nlm.nih.gov/gene/8480) | RAE1 | RAE1 RNA export 1 homolog (S. pombe) |
| [8843](http://www.ncbi.nlm.nih.gov/gene/8843) | HCAR3 | hydroxycarboxylic acid receptor 3 |
| [55190](http://www.ncbi.nlm.nih.gov/gene/55190) | NUDT11 | nudix (nucleoside diphosphate linked moiety X)-type motif 11 |
| [3812](http://www.ncbi.nlm.nih.gov/gene/3812) | KIR3DL2 | killer cell immunoglobulin-like receptor, three domains, long cytoplasmic tail, 2 |
| [100130733](http://www.ncbi.nlm.nih.gov/gene/100130733) | LRRC70 | leucine rich repeat containing 70 |
| [3735](http://www.ncbi.nlm.nih.gov/gene/3735) | KARS | lysyl-tRNA synthetase |
| [266812](http://www.ncbi.nlm.nih.gov/gene/266812) | NAP1L5 | nucleosome assembly protein 1-like 5 |
| [51230](http://www.ncbi.nlm.nih.gov/gene/51230) | PHF20 | PHD finger protein 20 |
| [5533](http://www.ncbi.nlm.nih.gov/gene/5533) | PPP3CC | protein phosphatase 3, catalytic subunit, gamma isozyme |
| [653784](http://www.ncbi.nlm.nih.gov/gene/653784) | MZT2A | mitotic spindle organizing protein 2A |
| [55219](http://www.ncbi.nlm.nih.gov/gene/55219) | TMEM57 | transmembrane protein 57 |
| [5153](http://www.ncbi.nlm.nih.gov/gene/5153) | PDE1B | phosphodiesterase 1B, calmodulin-dependent |
| [4683](http://www.ncbi.nlm.nih.gov/gene/4683) | NBN | nibrin |
| [359845](http://www.ncbi.nlm.nih.gov/gene/359845) | FAM101B | family with sequence similarity 101, member B |
| [5935](http://www.ncbi.nlm.nih.gov/gene/5935) | RBM3 | RNA binding motif (RNP1, RRM) protein 3 |
| [101059981](http://www.ncbi.nlm.nih.gov/gene/101059981) |  |  |
| [29028](http://www.ncbi.nlm.nih.gov/gene/29028) | ATAD2 | ATPase family, AAA domain containing 2 |
| [140461](http://www.ncbi.nlm.nih.gov/gene/140461) | ASB8 | ankyrin repeat and SOCS box containing 8 |
| [23533](http://www.ncbi.nlm.nih.gov/gene/23533) | PIK3R5 | phosphoinositide-3-kinase, regulatory subunit 5 |
| [3822](http://www.ncbi.nlm.nih.gov/gene/3822) | KLRC2 | killer cell lectin-like receptor subfamily C, member 2 |
| [115286](http://www.ncbi.nlm.nih.gov/gene/115286) | SLC25A26 | solute carrier family 25 (S-adenosylmethionine carrier), member 26 |
| [10602](http://www.ncbi.nlm.nih.gov/gene/10602) | CDC42EP3 | CDC42 effector protein (Rho GTPase binding) 3 |
| [100288637](http://www.ncbi.nlm.nih.gov/gene/100288637) | LOC100288637 | OTU domain containing 7A pseudogene |
| [6520](http://www.ncbi.nlm.nih.gov/gene/6520) | SLC3A2 | solute carrier family 3 (activators of dibasic and neutral amino acid transport), member 2 |
| [619571](http://www.ncbi.nlm.nih.gov/gene/619571) | SNORD96A | small nucleolar RNA, C/D box 96A |
| [10865](http://www.ncbi.nlm.nih.gov/gene/10865) | ARID5A | AT rich interactive domain 5A (MRF1-like) |
| [944](http://www.ncbi.nlm.nih.gov/gene/944) | TNFSF8 | tumor necrosis factor (ligand) superfamily, member 8 |

**Table 2. A list of vitamin D responsive genes whose expression levels downregulated after 6 months’ vitamin D_3_ supplementation (10,000 IU/d).**

| **Entrez Gene ID** | **Symbol** | **Description** |
| --- | --- | --- |
| [4087](http://www.ncbi.nlm.nih.gov/gene/4087) | SMAD2 | SMAD family member 2 |
| [11238](http://www.ncbi.nlm.nih.gov/gene/11238) | CA5B | carbonic anhydrase VB, mitochondrial |
| [26580](http://www.ncbi.nlm.nih.gov/gene/26580) | BSCL2 | Berardinelli-Seip congenital lipodystrophy 2 (seipin) |
| [7581](http://www.ncbi.nlm.nih.gov/gene/7581) | ZNF33A | zinc finger protein 33A |
| [57091](http://www.ncbi.nlm.nih.gov/gene/57091) | CASS4 | Cas scaffolding protein family member 4 |
| [10114](http://www.ncbi.nlm.nih.gov/gene/10114) | HIPK3 | homeodomain interacting protein kinase 3 |
| [54503](http://www.ncbi.nlm.nih.gov/gene/54503) | ZDHHC13 | zinc finger, DHHC-type containing 13 |
| [2618](http://www.ncbi.nlm.nih.gov/gene/2618) | GART | phosphoribosylglycinamide formyltransferase, phosphoribosylglycinamide synthetase, phosphoribosylaminoimidazole synthetase |
| [3480](http://www.ncbi.nlm.nih.gov/gene/3480) | IGF1R | insulin-like growth factor 1 receptor |
| [6777](http://www.ncbi.nlm.nih.gov/gene/6777) | STAT5B | signal transducer and activator of transcription 5B |
| [22900](http://www.ncbi.nlm.nih.gov/gene/22900) | CARD8 | caspase recruitment domain family, member 8 |
| [26610](http://www.ncbi.nlm.nih.gov/gene/26610) | ELP4 | elongator acetyltransferase complex subunit 4 |
| [63892](http://www.ncbi.nlm.nih.gov/gene/63892) | THADA | thyroid adenoma associated |
| [1666](http://www.ncbi.nlm.nih.gov/gene/1666) | DECR1 | 2,4-dienoyl CoA reductase 1, mitochondrial |
| [25801](http://www.ncbi.nlm.nih.gov/gene/25801) | GCA | grancalcin, EF-hand calcium binding protein |
| [317](http://www.ncbi.nlm.nih.gov/gene/317) | APAF1 | apoptotic peptidase activating factor 1 |
| [100133299](http://www.ncbi.nlm.nih.gov/gene/100133299) | LOC100133299 | GALI1870 |
| [7351](http://www.ncbi.nlm.nih.gov/gene/7351) | UCP2 | uncoupling protein 2 (mitochondrial, proton carrier) |
| [10902](http://www.ncbi.nlm.nih.gov/gene/10902) | BRD8 | bromodomain containing 8 |
| [84314](http://www.ncbi.nlm.nih.gov/gene/84314) | TMEM107 | transmembrane protein 107 |
| [2011](http://www.ncbi.nlm.nih.gov/gene/2011) | MARK2 | MAP/microtubule affinity-regulating kinase 2 |
| [1955](http://www.ncbi.nlm.nih.gov/gene/1955) | MEGF9 | multiple EGF-like-domains 9 |
| [57594](http://www.ncbi.nlm.nih.gov/gene/57594) | HOMEZ | homeobox and leucine zipper encoding |
| [10320](http://www.ncbi.nlm.nih.gov/gene/10320) | IKZF1 | IKAROS family zinc finger 1 (Ikaros) |
| [8243](http://www.ncbi.nlm.nih.gov/gene/8243) | SMC1A | structural maintenance of chromosomes 1A |
| [11025](http://www.ncbi.nlm.nih.gov/gene/11025) | LILRB3 | leukocyte immunoglobulin-like receptor, subfamily B (with TM and ITIM domains), member 3 |
| [3652](http://www.ncbi.nlm.nih.gov/gene/3652) | IPP | intracisternal A particle-promoted polypeptide |
| [23613](http://www.ncbi.nlm.nih.gov/gene/23613) | ZMYND8 | zinc finger, MYND-type containing 8 |
| [9849](http://www.ncbi.nlm.nih.gov/gene/9849) | ZNF518A | zinc finger protein 518A |
| [23708](http://www.ncbi.nlm.nih.gov/gene/23708) | GSPT2 | G1 to S phase transition 2 |
| [8228](http://www.ncbi.nlm.nih.gov/gene/8228) | PNPLA4 | patatin-like phospholipase domain containing 4 |
| [79415](http://www.ncbi.nlm.nih.gov/gene/79415) | C17orf62 | chromosome 17 open reading frame 62 |
| [55676](http://www.ncbi.nlm.nih.gov/gene/55676) | SLC30A6 | solute carrier family 30 (zinc transporter), member 6 |
| [92](http://www.ncbi.nlm.nih.gov/gene/92) | ACVR2A | activin A receptor, type IIA |
| [728661](http://www.ncbi.nlm.nih.gov/gene/728661) | SLC35E2B | solute carrier family 35, member E2B |
| [388558](http://www.ncbi.nlm.nih.gov/gene/388558) | ZNF808 | zinc finger protein 808 |
| [55589](http://www.ncbi.nlm.nih.gov/gene/55589) | BMP2K | BMP2 inducible kinase |
| [353345](http://www.ncbi.nlm.nih.gov/gene/353345) | GPR141 | G protein-coupled receptor 141 |
| [285440](http://www.ncbi.nlm.nih.gov/gene/285440) | CYP4V2 | cytochrome P450, family 4, subfamily V, polypeptide 2 |
| [51258](http://www.ncbi.nlm.nih.gov/gene/51258) | MRPL51 | mitochondrial ribosomal protein L51 |
| [60526](http://www.ncbi.nlm.nih.gov/gene/60526) | C2orf43 | chromosome 2 open reading frame 43 |
| [28449](http://www.ncbi.nlm.nih.gov/gene/28449) | IGHV3-13 | immunoglobulin heavy variable 3-13 |
| [9879](http://www.ncbi.nlm.nih.gov/gene/9879) | DDX46 | DEAD (Asp-Glu-Ala-Asp) box polypeptide 46 |
| [55723](http://www.ncbi.nlm.nih.gov/gene/55723) | ASF1B | ASF1 anti-silencing function 1 homolog B (S. cerevisiae) |
| [80216](http://www.ncbi.nlm.nih.gov/gene/80216) | ALPK1 | alpha-kinase 1 |
| [1021](http://www.ncbi.nlm.nih.gov/gene/1021) | CDK6 | cyclin-dependent kinase 6 |
| [3416](http://www.ncbi.nlm.nih.gov/gene/3416) | IDE | insulin-degrading enzyme |
| [25972](http://www.ncbi.nlm.nih.gov/gene/25972) | UNC50 | unc-50 homolog (C. elegans) |
| [5281](http://www.ncbi.nlm.nih.gov/gene/5281) | PIGF | phosphatidylinositol glycan anchor biosynthesis, class F |
| [345757](http://www.ncbi.nlm.nih.gov/gene/345757) | FAM174A | family with sequence similarity 174, member A |
| [4210](http://www.ncbi.nlm.nih.gov/gene/4210) | MEFV | Mediterranean fever |
| [84641](http://www.ncbi.nlm.nih.gov/gene/84641) | HIATL1 | hippocampus abundant transcript-like 1 |
| [23317](http://www.ncbi.nlm.nih.gov/gene/23317) | DNAJC13 | DnaJ (Hsp40) homolog, subfamily C, member 13 |
| [2213](http://www.ncbi.nlm.nih.gov/gene/2213) | FCGR2B | Fc fragment of IgG, low affinity IIb, receptor (CD32) |
| [4261](http://www.ncbi.nlm.nih.gov/gene/4261) | CIITA | class II, major histocompatibility complex, transactivator |
| [3575](http://www.ncbi.nlm.nih.gov/gene/3575) | IL7R | interleukin 7 receptor |
| [240](http://www.ncbi.nlm.nih.gov/gene/240) | ALOX5 | arachidonate 5-lipoxygenase |
| [5547](http://www.ncbi.nlm.nih.gov/gene/5547) | PRCP | prolylcarboxypeptidase (angiotensinase C) |
| [113251](http://www.ncbi.nlm.nih.gov/gene/113251) | LARP4 | La ribonucleoprotein domain family, member 4 |
| [3903](http://www.ncbi.nlm.nih.gov/gene/3903) | LAIR1 | leukocyte-associated immunoglobulin-like receptor 1 |
| [401884](http://www.ncbi.nlm.nih.gov/gene/401884) | MGC57346 | uncharacterized LOC401884 |
| [8106](http://www.ncbi.nlm.nih.gov/gene/8106) | PABPN1 | poly(A) binding protein, nuclear 1 |
| [51540](http://www.ncbi.nlm.nih.gov/gene/51540) | SCLY | selenocysteine lyase |
| [26263](http://www.ncbi.nlm.nih.gov/gene/26263) | FBXO22 | F-box protein 22 |
| [9786](http://www.ncbi.nlm.nih.gov/gene/9786) | KIAA0586 | KIAA0586 |
| [3097](http://www.ncbi.nlm.nih.gov/gene/3097) | HIVEP2 | human immunodeficiency virus type I enhancer binding protein 2 |
| [10023](http://www.ncbi.nlm.nih.gov/gene/10023) | FRAT1 | frequently rearranged in advanced T-cell lymphomas |
| [283232](http://www.ncbi.nlm.nih.gov/gene/283232) | TMEM80 | transmembrane protein 80 |
| [55601](http://www.ncbi.nlm.nih.gov/gene/55601) | DDX60 | DEAD (Asp-Glu-Ala-Asp) box polypeptide 60 |
| [10636](http://www.ncbi.nlm.nih.gov/gene/10636) | RGS14 | regulator of G-protein signaling 14 |
| [9488](http://www.ncbi.nlm.nih.gov/gene/9488) | PIGB | phosphatidylinositol glycan anchor biosynthesis, class B |
| [54809](http://www.ncbi.nlm.nih.gov/gene/54809) | SAMD9 | sterile alpha motif domain containing 9 |
| [9793](http://www.ncbi.nlm.nih.gov/gene/9793) | CKAP5 | cytoskeleton associated protein 5 |
| [51010](http://www.ncbi.nlm.nih.gov/gene/51010) | EXOSC3 | exosome component 3 |
| [51643](http://www.ncbi.nlm.nih.gov/gene/51643) | TMBIM4 | transmembrane BAX inhibitor motif containing 4 |
| [10161](http://www.ncbi.nlm.nih.gov/gene/10161) | LPAR6 | lysophosphatidic acid receptor 6 |
| [911](http://www.ncbi.nlm.nih.gov/gene/911) | CD1C | CD1c molecule |
| [26289](http://www.ncbi.nlm.nih.gov/gene/26289) | AK5 | adenylate kinase 5 |
| [1612](http://www.ncbi.nlm.nih.gov/gene/1612) | DAPK1 | death-associated protein kinase 1 |
| [348235](http://www.ncbi.nlm.nih.gov/gene/348235) | SKA2 | spindle and kinetochore associated complex subunit 2 |
| [6850](http://www.ncbi.nlm.nih.gov/gene/6850) | SYK | spleen tyrosine kinase |
| [23446](http://www.ncbi.nlm.nih.gov/gene/23446) | SLC44A1 | solute carrier family 44, member 1 |
| [83480](http://www.ncbi.nlm.nih.gov/gene/83480) | PUS3 | pseudouridylate synthase 3 |
| [80010](http://www.ncbi.nlm.nih.gov/gene/80010) | RMI1 | RMI1, RecQ mediated genome instability 1, homolog (S. cerevisiae) |
| [10771](http://www.ncbi.nlm.nih.gov/gene/10771) | ZMYND11 | zinc finger, MYND-type containing 11 |
| [284443](http://www.ncbi.nlm.nih.gov/gene/284443) | ZNF493 | zinc finger protein 493 |
| [154](http://www.ncbi.nlm.nih.gov/gene/154) | ADRB2 | adrenoceptor beta 2, surface |
| [55900](http://www.ncbi.nlm.nih.gov/gene/55900) | ZNF302 | zinc finger protein 302 |
| [2585](http://www.ncbi.nlm.nih.gov/gene/2585) | GALK2 | galactokinase 2 |
| [56144](http://www.ncbi.nlm.nih.gov/gene/56144) | PCDHA4 | protocadherin alpha 4 |
| [337977](http://www.ncbi.nlm.nih.gov/gene/337977) | KRTAP21-1 | keratin associated protein 21-1 |
| [113277](http://www.ncbi.nlm.nih.gov/gene/113277) | TMEM106A | transmembrane protein 106A |
| [25939](http://www.ncbi.nlm.nih.gov/gene/25939) | SAMHD1 | SAM domain and HD domain 1 |
| [91010](http://www.ncbi.nlm.nih.gov/gene/91010) | FMNL3 | formin-like 3 |
| [51170](http://www.ncbi.nlm.nih.gov/gene/51170) | HSD17B11 | hydroxysteroid (17-beta) dehydrogenase 11 |
| [339318](http://www.ncbi.nlm.nih.gov/gene/339318) | ZNF181 | zinc finger protein 181 |
| [9252](http://www.ncbi.nlm.nih.gov/gene/9252) | RPS6KA5 | ribosomal protein S6 kinase, 90kDa, polypeptide 5 |
| [79901](http://www.ncbi.nlm.nih.gov/gene/79901) | CYBRD1 | cytochrome b reductase 1 |
| [3417](http://www.ncbi.nlm.nih.gov/gene/3417) | IDH1 | isocitrate dehydrogenase 1 (NADP+), soluble |
| [219972](http://www.ncbi.nlm.nih.gov/gene/219972) | MPEG1 | macrophage expressed 1 |
| [6654](http://www.ncbi.nlm.nih.gov/gene/6654) | SOS1 | son of sevenless homolog 1 (Drosophila) |
| [56912](http://www.ncbi.nlm.nih.gov/gene/56912) | IFT46 | intraflagellar transport 46 homolog (Chlamydomonas) |
| [90](http://www.ncbi.nlm.nih.gov/gene/90) | ACVR1 | activin A receptor, type I |
| [7132](http://www.ncbi.nlm.nih.gov/gene/7132) | TNFRSF1A | tumor necrosis factor receptor superfamily, member 1A |
| [5795](http://www.ncbi.nlm.nih.gov/gene/5795) | PTPRJ | protein tyrosine phosphatase, receptor type, J |
| [8790](http://www.ncbi.nlm.nih.gov/gene/8790) | FPGT | fucose-1-phosphate guanylyltransferase |
| [2776](http://www.ncbi.nlm.nih.gov/gene/2776) | GNAQ | guanine nucleotide binding protein (G protein), q polypeptide |
| [55374](http://www.ncbi.nlm.nih.gov/gene/55374) | TMCO6 | transmembrane and coiled-coil domains 6 |
| [23225](http://www.ncbi.nlm.nih.gov/gene/23225) | NUP210 | nucleoporin 210kDa |
| [5530](http://www.ncbi.nlm.nih.gov/gene/5530) | PPP3CA | protein phosphatase 3, catalytic subunit, alpha isozyme |
| [8972](http://www.ncbi.nlm.nih.gov/gene/8972) | MGAM | maltase-glucoamylase (alpha-glucosidase) |
| [6120](http://www.ncbi.nlm.nih.gov/gene/6120) | RPE | ribulose-5-phosphate-3-epimerase |
| [538](http://www.ncbi.nlm.nih.gov/gene/538) | ATP7A | ATPase, Cu++ transporting, alpha polypeptide |
| [57552](http://www.ncbi.nlm.nih.gov/gene/57552) | NCEH1 | neutral cholesterol ester hydrolase 1 |
| [5162](http://www.ncbi.nlm.nih.gov/gene/5162) | PDHB | pyruvate dehydrogenase (lipoamide) beta |
| [6252](http://www.ncbi.nlm.nih.gov/gene/6252) | RTN1 | reticulon 1 |
| [122553](http://www.ncbi.nlm.nih.gov/gene/122553) | TRAPPC6B | trafficking protein particle complex 6B |
| [51077](http://www.ncbi.nlm.nih.gov/gene/51077) | FCF1 | FCF1 small subunit (SSU) processome component homolog (S. cerevisiae) |
| [4258](http://www.ncbi.nlm.nih.gov/gene/4258) | MGST2 | microsomal glutathione S-transferase 2 |
| [5550](http://www.ncbi.nlm.nih.gov/gene/5550) | PREP | prolyl endopeptidase |
| [2214](http://www.ncbi.nlm.nih.gov/gene/2214) | FCGR3A | Fc fragment of IgG, low affinity IIIa, receptor (CD16a) |
| [1808](http://www.ncbi.nlm.nih.gov/gene/1808) | DPYSL2 | dihydropyrimidinase-like 2 |
| [58486](http://www.ncbi.nlm.nih.gov/gene/58486) | ZBED5 | zinc finger, BED-type containing 5 |
| [201895](http://www.ncbi.nlm.nih.gov/gene/201895) | C4orf34 | chromosome 4 open reading frame 34 |
| [102](http://www.ncbi.nlm.nih.gov/gene/102) | ADAM10 | ADAM metallopeptidase domain 10 |
| [51106](http://www.ncbi.nlm.nih.gov/gene/51106) | TFB1M | transcription factor B1, mitochondrial |
| [6036](http://www.ncbi.nlm.nih.gov/gene/6036) | RNASE2 | ribonuclease, RNase A family, 2 (liver, eosinophil-derived neurotoxin) |
| [152185](http://www.ncbi.nlm.nih.gov/gene/152185) | SPICE1 | spindle and centriole associated protein 1 |
| [471](http://www.ncbi.nlm.nih.gov/gene/471) | ATIC | 5-aminoimidazole-4-carboxamide ribonucleotide formyltransferase/IMP cyclohydrolase |
| [81704](http://www.ncbi.nlm.nih.gov/gene/81704) | DOCK8 | dedicator of cytokinesis 8 |
| [23344](http://www.ncbi.nlm.nih.gov/gene/23344) | ESYT1 | extended synaptotagmin-like protein 1 |
| [114804](http://www.ncbi.nlm.nih.gov/gene/114804) | RNF157 | ring finger protein 157 |
| [84460](http://www.ncbi.nlm.nih.gov/gene/84460) | ZMAT1 | zinc finger, matrin-type 1 |
| [80195](http://www.ncbi.nlm.nih.gov/gene/80195) | C10orf57 | chromosome 10 open reading frame 57 |
| [27340](http://www.ncbi.nlm.nih.gov/gene/27340) | UTP20 | UTP20, small subunit (SSU) processome component, homolog (yeast) |
| [6502](http://www.ncbi.nlm.nih.gov/gene/6502) | SKP2 | S-phase kinase-associated protein 2, E3 ubiquitin protein ligase |
| [54482](http://www.ncbi.nlm.nih.gov/gene/54482) | TRMT13 | tRNA methyltransferase 13 homolog (S. cerevisiae) |
| [7074](http://www.ncbi.nlm.nih.gov/gene/7074) | TIAM1 | T-cell lymphoma invasion and metastasis 1 |
| [55188](http://www.ncbi.nlm.nih.gov/gene/55188) | RIC8B | resistance to inhibitors of cholinesterase 8 homolog B (C. elegans) |
| [79845](http://www.ncbi.nlm.nih.gov/gene/79845) | RNF122 | ring finger protein 122 |
| [406991](http://www.ncbi.nlm.nih.gov/gene/406991) | MIR21 | microRNA 21 |
| [5437](http://www.ncbi.nlm.nih.gov/gene/5437) | POLR2H | polymerase (RNA) II (DNA directed) polypeptide H |
| [1359](http://www.ncbi.nlm.nih.gov/gene/1359) | CPA3 | carboxypeptidase A3 (mast cell) |
| [113540](http://www.ncbi.nlm.nih.gov/gene/113540) | CMTM1 | CKLF-like MARVEL transmembrane domain containing 1 |
| [2909](http://www.ncbi.nlm.nih.gov/gene/2909) | ARHGAP35 | Rho GTPase activating protein 35 |
| [10008](http://www.ncbi.nlm.nih.gov/gene/10008) | KCNE3 | potassium voltage-gated channel, Isk-related family, member 3 |
| [8473](http://www.ncbi.nlm.nih.gov/gene/8473) | OGT | O-linked N-acetylglucosamine (GlcNAc) transferase |
| [9289](http://www.ncbi.nlm.nih.gov/gene/9289) | GPR56 | G protein-coupled receptor 56 |
| [79188](http://www.ncbi.nlm.nih.gov/gene/79188) | TMEM43 | transmembrane protein 43 |
| [730094](http://www.ncbi.nlm.nih.gov/gene/730094) | C16orf52 | chromosome 16 open reading frame 52 |
| [1232](http://www.ncbi.nlm.nih.gov/gene/1232) | CCR3 | chemokine (C-C motif) receptor 3 |
| [51095](http://www.ncbi.nlm.nih.gov/gene/51095) | TRNT1 | tRNA nucleotidyl transferase, CCA-adding, 1 |
| [81929](http://www.ncbi.nlm.nih.gov/gene/81929) | SEH1L | SEH1-like (S. cerevisiae) |
| [400506](http://www.ncbi.nlm.nih.gov/gene/400506) | C16orf88 | chromosome 16 open reading frame 88 |
| [220988](http://www.ncbi.nlm.nih.gov/gene/220988) | HNRNPA3 | heterogeneous nuclear ribonucleoprotein A3 |
| [57616](http://www.ncbi.nlm.nih.gov/gene/57616) | TSHZ3 | teashirt zinc finger homeobox 3 |
| [55759](http://www.ncbi.nlm.nih.gov/gene/55759) | WDR12 | WD repeat domain 12 |
| [55756](http://www.ncbi.nlm.nih.gov/gene/55756) | INTS9 | integrator complex subunit 9 |
| [56915](http://www.ncbi.nlm.nih.gov/gene/56915) | EXOSC5 | exosome component 5 |
| [128869](http://www.ncbi.nlm.nih.gov/gene/128869) | PIGU | phosphatidylinositol glycan anchor biosynthesis, class U |
| [22908](http://www.ncbi.nlm.nih.gov/gene/22908) | SACM1L | SAC1 suppressor of actin mutations 1-like (yeast) |
| [27304](http://www.ncbi.nlm.nih.gov/gene/27304) | MOCS3 | molybdenum cofactor synthesis 3 |
| [5229](http://www.ncbi.nlm.nih.gov/gene/5229) | PGGT1B | protein geranylgeranyltransferase type I, beta subunit |
| [894](http://www.ncbi.nlm.nih.gov/gene/894) | CCND2 | cyclin D2 |
| [222234](http://www.ncbi.nlm.nih.gov/gene/222234) | FAM185A | family with sequence similarity 185, member A |
| [212](http://www.ncbi.nlm.nih.gov/gene/212) | ALAS2 | aminolevulinate, delta-, synthase 2 |
| [84671](http://www.ncbi.nlm.nih.gov/gene/84671) | ZNF347 | zinc finger protein 347 |
| [140609](http://www.ncbi.nlm.nih.gov/gene/140609) | NEK7 | NIMA (never in mitosis gene a)-related kinase 7 |
| [157247](http://www.ncbi.nlm.nih.gov/gene/157247) | MGC27345 | uncharacterized protein MGC27345 |
| [9241](http://www.ncbi.nlm.nih.gov/gene/9241) | NOG | noggin |
| [10559](http://www.ncbi.nlm.nih.gov/gene/10559) | SLC35A1 | solute carrier family 35 (CMP-sialic acid transporter), member A1 |
| [634](http://www.ncbi.nlm.nih.gov/gene/634) | CEACAM1 | carcinoembryonic antigen-related cell adhesion molecule 1 (biliary glycoprotein) |
| [27010](http://www.ncbi.nlm.nih.gov/gene/27010) | TPK1 | thiamin pyrophosphokinase 1 |
| [23019](http://www.ncbi.nlm.nih.gov/gene/23019) | CNOT1 | CCR4-NOT transcription complex, subunit 1 |
| [25984](http://www.ncbi.nlm.nih.gov/gene/25984) | KRT23 | keratin 23 (histone deacetylase inducible) |
| [29071](http://www.ncbi.nlm.nih.gov/gene/29071) | C1GALT1C1 | C1GALT1-specific chaperone 1 |
| [339324](http://www.ncbi.nlm.nih.gov/gene/339324) | ZNF260 | zinc finger protein 260 |
| [9070](http://www.ncbi.nlm.nih.gov/gene/9070) | ASH2L | ash2 (absent, small, or homeotic)-like (Drosophila) |
| [643836](http://www.ncbi.nlm.nih.gov/gene/643836) | ZFP62 | zinc finger protein 62 homolog (mouse) |
| [10935](http://www.ncbi.nlm.nih.gov/gene/10935) | PRDX3 | peroxiredoxin 3 |
| [27036](http://www.ncbi.nlm.nih.gov/gene/27036) | SIGLEC7 | sialic acid binding Ig-like lectin 7 |
| [5743](http://www.ncbi.nlm.nih.gov/gene/5743) | PTGS2 | prostaglandin-endoperoxide synthase 2 (prostaglandin G/H synthase and cyclooxygenase) |
| [1823](http://www.ncbi.nlm.nih.gov/gene/1823) | DSC1 | desmocollin 1 |
| [526](http://www.ncbi.nlm.nih.gov/gene/526) | ATP6V1B2 | ATPase, H+ transporting, lysosomal 56/58kDa, V1 subunit B2 |
| [60491](http://www.ncbi.nlm.nih.gov/gene/60491) | NIF3L1 | NIF3 NGG1 interacting factor 3-like 1 (S. cerevisiae) |
| [60675](http://www.ncbi.nlm.nih.gov/gene/60675) | PROK2 | prokineticin 2 |
| [79652](http://www.ncbi.nlm.nih.gov/gene/79652) | TMEM204 | transmembrane protein 204 |
| [91351](http://www.ncbi.nlm.nih.gov/gene/91351) | DDX60L | DEAD (Asp-Glu-Ala-Asp) box polypeptide 60-like |
| [11000](http://www.ncbi.nlm.nih.gov/gene/11000) | SLC27A3 | solute carrier family 27 (fatty acid transporter), member 3 |
| [81542](http://www.ncbi.nlm.nih.gov/gene/81542) | TMX1 | thioredoxin-related transmembrane protein 1 |
| [84955](http://www.ncbi.nlm.nih.gov/gene/84955) | NUDCD1 | NudC domain containing 1 |
| [54847](http://www.ncbi.nlm.nih.gov/gene/54847) | SIDT1 | SID1 transmembrane family, member 1 |
| [140564](http://www.ncbi.nlm.nih.gov/gene/140564) | APOBEC3D | apolipoprotein B mRNA editing enzyme, catalytic polypeptide-like 3D |
| [85025](http://www.ncbi.nlm.nih.gov/gene/85025) | TMEM60 | transmembrane protein 60 |
| [84527](http://www.ncbi.nlm.nih.gov/gene/84527) | ZNF559 | zinc finger protein 559 |
| [307](http://www.ncbi.nlm.nih.gov/gene/307) | ANXA4 | annexin A4 |
| [285527](http://www.ncbi.nlm.nih.gov/gene/285527) | FRYL | FRY-like |
| [472](http://www.ncbi.nlm.nih.gov/gene/472) | ATM | ataxia telangiectasia mutated |
| [2592](http://www.ncbi.nlm.nih.gov/gene/2592) | GALT | galactose-1-phosphate uridylyltransferase |
| [6619](http://www.ncbi.nlm.nih.gov/gene/6619) | SNAPC3 | small nuclear RNA activating complex, polypeptide 3, 50kDa |
| [65981](http://www.ncbi.nlm.nih.gov/gene/65981) | CAPRIN2 | caprin family member 2 |
| [8405](http://www.ncbi.nlm.nih.gov/gene/8405) | SPOP | speckle-type POZ protein |
| [3305](http://www.ncbi.nlm.nih.gov/gene/3305) | HSPA1L | heat shock 70kDa protein 1-like |
| [55751](http://www.ncbi.nlm.nih.gov/gene/55751) | TMEM184C | transmembrane protein 184C |
| [140707](http://www.ncbi.nlm.nih.gov/gene/140707) | BRI3BP | BRI3 binding protein |
| [93627](http://www.ncbi.nlm.nih.gov/gene/93627) | TBCK | TBC1 domain containing kinase |
| [9645](http://www.ncbi.nlm.nih.gov/gene/9645) | MICAL2 | microtubule associated monoxygenase, calponin and LIM domain containing 2 |
| [63897](http://www.ncbi.nlm.nih.gov/gene/63897) | HEATR6 | HEAT repeat containing 6 |
| [152189](http://www.ncbi.nlm.nih.gov/gene/152189) | CMTM8 | CKLF-like MARVEL transmembrane domain containing 8 |
| [80350](http://www.ncbi.nlm.nih.gov/gene/80350) | LPAL2 | lipoprotein, Lp(a)-like 2, pseudogene |
| [3676](http://www.ncbi.nlm.nih.gov/gene/3676) | ITGA4 | integrin, alpha 4 (antigen CD49D, alpha 4 subunit of VLA-4 receptor) |
| [10610](http://www.ncbi.nlm.nih.gov/gene/10610) | ST6GALNAC2 | ST6 (alpha-N-acetyl-neuraminyl-2,3-beta-galactosyl-1,3)-N-acetylgalactosaminide alpha-2,6-sialyltransferase 2 |
| [90139](http://www.ncbi.nlm.nih.gov/gene/90139) | TSPAN18 | tetraspanin 18 |
| [79020](http://www.ncbi.nlm.nih.gov/gene/79020) | C7orf25 | chromosome 7 open reading frame 25 |
| [8195](http://www.ncbi.nlm.nih.gov/gene/8195) | MKKS | McKusick-Kaufman syndrome |
| [125893](http://www.ncbi.nlm.nih.gov/gene/125893) | ZNF816 | zinc finger protein 816 |
| [4332](http://www.ncbi.nlm.nih.gov/gene/4332) | MNDA | myeloid cell nuclear differentiation antigen |
| [23450](http://www.ncbi.nlm.nih.gov/gene/23450) | SF3B3 | splicing factor 3b, subunit 3, 130kDa |
| [90649](http://www.ncbi.nlm.nih.gov/gene/90649) | ZNF486 | zinc finger protein 486 |
| [54682](http://www.ncbi.nlm.nih.gov/gene/54682) | MANSC1 | MANSC domain containing 1 |
| [225](http://www.ncbi.nlm.nih.gov/gene/225) | ABCD2 | ATP-binding cassette, sub-family D (ALD), member 2 |
| [1154](http://www.ncbi.nlm.nih.gov/gene/1154) | CISH | cytokine inducible SH2-containing protein |
| [93183](http://www.ncbi.nlm.nih.gov/gene/93183) | PIGM | phosphatidylinositol glycan anchor biosynthesis, class M |
| [7107](http://www.ncbi.nlm.nih.gov/gene/7107) | GPR137B | G protein-coupled receptor 137B |
| [169693](http://www.ncbi.nlm.nih.gov/gene/169693) | TMEM252 | transmembrane protein 252 |
| [56172](http://www.ncbi.nlm.nih.gov/gene/56172) | ANKH | ankylosis, progressive homolog (mouse) |
| [151636](http://www.ncbi.nlm.nih.gov/gene/151636) | DTX3L | deltex 3-like (Drosophila) |
| [1230](http://www.ncbi.nlm.nih.gov/gene/1230) | CCR1 | chemokine (C-C motif) receptor 1 |
| [10205](http://www.ncbi.nlm.nih.gov/gene/10205) | MPZL2 | myelin protein zero-like 2 |
| [8826](http://www.ncbi.nlm.nih.gov/gene/8826) | IQGAP1 | IQ motif containing GTPase activating protein 1 |
| [929](http://www.ncbi.nlm.nih.gov/gene/929) | CD14 | CD14 molecule |
| [10098](http://www.ncbi.nlm.nih.gov/gene/10098) | TSPAN5 | tetraspanin 5 |
| [939](http://www.ncbi.nlm.nih.gov/gene/939) | CD27 | CD27 molecule |
| [86](http://www.ncbi.nlm.nih.gov/gene/86) | ACTL6A | actin-like 6A |
| [84329](http://www.ncbi.nlm.nih.gov/gene/84329) | HVCN1 | hydrogen voltage-gated channel 1 |
| [83605](http://www.ncbi.nlm.nih.gov/gene/83605) | CCM2 | cerebral cavernous malformation 2 |
| [8115](http://www.ncbi.nlm.nih.gov/gene/8115) | TCL1A | T-cell leukemia/lymphoma 1A |
| [51155](http://www.ncbi.nlm.nih.gov/gene/51155) | HN1 | hematological and neurological expressed 1 |
| [51313](http://www.ncbi.nlm.nih.gov/gene/51313) | FAM198B | family with sequence similarity 198, member B |
| [9404](http://www.ncbi.nlm.nih.gov/gene/9404) | LPXN | leupaxin |
| [10890](http://www.ncbi.nlm.nih.gov/gene/10890) | RAB10 | RAB10, member RAS oncogene family |
| [64921](http://www.ncbi.nlm.nih.gov/gene/64921) | CASD1 | CAS1 domain containing 1 |
| [55276](http://www.ncbi.nlm.nih.gov/gene/55276) | PGM2 | phosphoglucomutase 2 |
| [253461](http://www.ncbi.nlm.nih.gov/gene/253461) | ZBTB38 | zinc finger and BTB domain containing 38 |
| [151507](http://www.ncbi.nlm.nih.gov/gene/151507) | MSL3P1 | male-specific lethal 3 homolog (Drosophila) pseudogene 1 |
| [11010](http://www.ncbi.nlm.nih.gov/gene/11010) | GLIPR1 | GLI pathogenesis-related 1 |
| [100302742](http://www.ncbi.nlm.nih.gov/gene/100302742) | RNU6-42 | RNA, U6 small nuclear 42 |
| [51](http://www.ncbi.nlm.nih.gov/gene/51) | ACOX1 | acyl-CoA oxidase 1, palmitoyl |
| [162466](http://www.ncbi.nlm.nih.gov/gene/162466) | PHOSPHO1 | phosphatase, orphan 1 |
| [25896](http://www.ncbi.nlm.nih.gov/gene/25896) | INTS7 | integrator complex subunit 7 |
| [26788](http://www.ncbi.nlm.nih.gov/gene/26788) | SNORD60 | small nucleolar RNA, C/D box 60 |
| [3482](http://www.ncbi.nlm.nih.gov/gene/3482) | IGF2R | insulin-like growth factor 2 receptor |
| [528](http://www.ncbi.nlm.nih.gov/gene/528) | ATP6V1C1 | ATPase, H+ transporting, lysosomal 42kDa, V1 subunit C1 |
| [63901](http://www.ncbi.nlm.nih.gov/gene/63901) | FAM111A | family with sequence similarity 111, member A |
| [22918](http://www.ncbi.nlm.nih.gov/gene/22918) | CD93 | CD93 molecule |
| [3514](http://www.ncbi.nlm.nih.gov/gene/3514) | IGKC | immunoglobulin kappa constant |
| [8794](http://www.ncbi.nlm.nih.gov/gene/8794) | TNFRSF10C | tumor necrosis factor receptor superfamily, member 10c, decoy without an intracellular domain |
| [643418](http://www.ncbi.nlm.nih.gov/gene/643418) | LIPN | lipase, family member N |
| [80267](http://www.ncbi.nlm.nih.gov/gene/80267) | EDEM3 | ER degradation enhancer, mannosidase alpha-like 3 |
| [11191](http://www.ncbi.nlm.nih.gov/gene/11191) | PTENP1 | phosphatase and tensin homolog pseudogene 1 |
| [8402](http://www.ncbi.nlm.nih.gov/gene/8402) | SLC25A11 | solute carrier family 25 (mitochondrial carrier; oxoglutarate carrier), member 11 |
| [3310](http://www.ncbi.nlm.nih.gov/gene/3310) | HSPA6 | heat shock 70kDa protein 6 (HSP70B') |
| [160364](http://www.ncbi.nlm.nih.gov/gene/160364) | CLEC12A | C-type lectin domain family 12, member A |
| [343413](http://www.ncbi.nlm.nih.gov/gene/343413) | FCRL6 | Fc receptor-like 6 |
| [2629](http://www.ncbi.nlm.nih.gov/gene/2629) | GBA | glucosidase, beta, acid |
| [51816](http://www.ncbi.nlm.nih.gov/gene/51816) | CECR1 | cat eye syndrome chromosome region, candidate 1 |
| [6653](http://www.ncbi.nlm.nih.gov/gene/6653) | SORL1 | sortilin-related receptor, L(DLR class) A repeats containing |
| [389607](http://www.ncbi.nlm.nih.gov/gene/389607) | LOC389607 | uncharacterized LOC389607 |
| [1019](http://www.ncbi.nlm.nih.gov/gene/1019) | CDK4 | cyclin-dependent kinase 4 |
| [5893](http://www.ncbi.nlm.nih.gov/gene/5893) | RAD52 | RAD52 homolog (S. cerevisiae) |
| [9881](http://www.ncbi.nlm.nih.gov/gene/9881) | TRANK1 | tetratricopeptide repeat and ankyrin repeat containing 1 |
| [168537](http://www.ncbi.nlm.nih.gov/gene/168537) | GIMAP7 | GTPase, IMAP family member 7 |
| [353088](http://www.ncbi.nlm.nih.gov/gene/353088) | ZNF429 | zinc finger protein 429 |
| [10333](http://www.ncbi.nlm.nih.gov/gene/10333) | TLR6 | toll-like receptor 6 |
| [474344](http://www.ncbi.nlm.nih.gov/gene/474344) | GIMAP6 | GTPase, IMAP family member 6 |
| [3570](http://www.ncbi.nlm.nih.gov/gene/3570) | IL6R | interleukin 6 receptor |
| [55313](http://www.ncbi.nlm.nih.gov/gene/55313) | CPPED1 | calcineurin-like phosphoesterase domain containing 1 |
| [222236](http://www.ncbi.nlm.nih.gov/gene/222236) | NAPEPLD | N-acyl phosphatidylethanolamine phospholipase D |
| [84450](http://www.ncbi.nlm.nih.gov/gene/84450) | ZNF512 | zinc finger protein 512 |
| [91662](http://www.ncbi.nlm.nih.gov/gene/91662) | NLRP12 | NLR family, pyrin domain containing 12 |
| [1436](http://www.ncbi.nlm.nih.gov/gene/1436) | CSF1R | colony stimulating factor 1 receptor |
| [4939](http://www.ncbi.nlm.nih.gov/gene/4939) | OAS2 | 2'-5'-oligoadenylate synthetase 2, 69/71kDa |
| [64863](http://www.ncbi.nlm.nih.gov/gene/64863) | METTL4 | methyltransferase like 4 |
| [23348](http://www.ncbi.nlm.nih.gov/gene/23348) | DOCK9 | dedicator of cytokinesis 9 |
| [100288594](http://www.ncbi.nlm.nih.gov/gene/100288594) | LOC100288594 | uncharacterized LOC100288594 |
| [7587](http://www.ncbi.nlm.nih.gov/gene/7587) | ZNF37A | zinc finger protein 37A |
| [84105](http://www.ncbi.nlm.nih.gov/gene/84105) | PCBD2 | pterin-4 alpha-carbinolamine dehydratase/dimerization cofactor of hepatocyte nuclear factor 1 alpha (TCF1) 2 |
| [162962](http://www.ncbi.nlm.nih.gov/gene/162962) | ZNF836 | zinc finger protein 836 |
| [2153](http://www.ncbi.nlm.nih.gov/gene/2153) | F5 | coagulation factor V (proaccelerin, labile factor) |
| [55791](http://www.ncbi.nlm.nih.gov/gene/55791) | LRIF1 | ligand dependent nuclear receptor interacting factor 1 |
| [10592](http://www.ncbi.nlm.nih.gov/gene/10592) | SMC2 | structural maintenance of chromosomes 2 |
| [5557](http://www.ncbi.nlm.nih.gov/gene/5557) | PRIM1 | primase, DNA, polypeptide 1 (49kDa) |
| [9562](http://www.ncbi.nlm.nih.gov/gene/9562) | MINPP1 | multiple inositol-polyphosphate phosphatase 1 |
| [9674](http://www.ncbi.nlm.nih.gov/gene/9674) | KIAA0040 | KIAA0040 |
| [79056](http://www.ncbi.nlm.nih.gov/gene/79056) | PRRG4 | proline rich Gla (G-carboxyglutamic acid) 4 (transmembrane) |
| [6546](http://www.ncbi.nlm.nih.gov/gene/6546) | SLC8A1 | solute carrier family 8 (sodium/calcium exchanger), member 1 |
| [10238](http://www.ncbi.nlm.nih.gov/gene/10238) | DCAF7 | DDB1 and CUL4 associated factor 7 |
| [353189](http://www.ncbi.nlm.nih.gov/gene/353189) | SLCO4C1 | solute carrier organic anion transporter family, member 4C1 |
| [84689](http://www.ncbi.nlm.nih.gov/gene/84689) | MS4A14 | membrane-spanning 4-domains, subfamily A, member 14 |
| [29887](http://www.ncbi.nlm.nih.gov/gene/29887) | SNX10 | sorting nexin 10 |
| [10384](http://www.ncbi.nlm.nih.gov/gene/10384) | BTN3A3 | butyrophilin, subfamily 3, member A3 |
| [6947](http://www.ncbi.nlm.nih.gov/gene/6947) | TCN1 | transcobalamin I (vitamin B12 binding protein, R binder family) |
| [54497](http://www.ncbi.nlm.nih.gov/gene/54497) | HEATR5B | HEAT repeat containing 5B |
| [729230](http://www.ncbi.nlm.nih.gov/gene/729230) | CCR2 | chemokine (C-C motif) receptor 2 |
| [9526](http://www.ncbi.nlm.nih.gov/gene/9526) | MPDU1 | mannose-P-dolichol utilization defect 1 |
| [51434](http://www.ncbi.nlm.nih.gov/gene/51434) | ANAPC7 | anaphase promoting complex subunit 7 |
| [55140](http://www.ncbi.nlm.nih.gov/gene/55140) | ELP3 | elongator acetyltransferase complex subunit 3 |
| [100132979](http://www.ncbi.nlm.nih.gov/gene/100132979) | GOLGA8DP | golgin A8 family, member D, pseudogene |
| [29058](http://www.ncbi.nlm.nih.gov/gene/29058) | TMEM230 | transmembrane protein 230 |
| [253558](http://www.ncbi.nlm.nih.gov/gene/253558) | LCLAT1 | lysocardiolipin acyltransferase 1 |
| [64430](http://www.ncbi.nlm.nih.gov/gene/64430) | PCNXL4 | pecanex-like 4 (Drosophila) |
| [2188](http://www.ncbi.nlm.nih.gov/gene/2188) | FANCF | Fanconi anemia, complementation group F |
| [10100](http://www.ncbi.nlm.nih.gov/gene/10100) | TSPAN2 | tetraspanin 2 |
| [53829](http://www.ncbi.nlm.nih.gov/gene/53829) | P2RY13 | purinergic receptor P2Y, G-protein coupled, 13 |
| [10146](http://www.ncbi.nlm.nih.gov/gene/10146) | G3BP1 | GTPase activating protein (SH3 domain) binding protein 1 |
| [10269](http://www.ncbi.nlm.nih.gov/gene/10269) | ZMPSTE24 | zinc metallopeptidase STE24 homolog (S. cerevisiae) |
| [79768](http://www.ncbi.nlm.nih.gov/gene/79768) | C15orf29 | chromosome 15 open reading frame 29 |
| [26235](http://www.ncbi.nlm.nih.gov/gene/26235) | FBXL4 | F-box and leucine-rich repeat protein 4 |
| [115352](http://www.ncbi.nlm.nih.gov/gene/115352) | FCRL3 | Fc receptor-like 3 |
| [9194](http://www.ncbi.nlm.nih.gov/gene/9194) | SLC16A7 | solute carrier family 16, member 7 (monocarboxylic acid transporter 2) |
| [2313](http://www.ncbi.nlm.nih.gov/gene/2313) | FLI1 | Friend leukemia virus integration 1 |
| [26119](http://www.ncbi.nlm.nih.gov/gene/26119) | LDLRAP1 | low density lipoprotein receptor adaptor protein 1 |
| [403323](http://www.ncbi.nlm.nih.gov/gene/403323) | FLJ20444 | uncharacterized protein FLJ20444 |
| [9447](http://www.ncbi.nlm.nih.gov/gene/9447) | AIM2 | absent in melanoma 2 |
| [7358](http://www.ncbi.nlm.nih.gov/gene/7358) | UGDH | UDP-glucose 6-dehydrogenase |
| [1439](http://www.ncbi.nlm.nih.gov/gene/1439) | CSF2RB | colony stimulating factor 2 receptor, beta, low-affinity (granulocyte-macrophage) |
| [253430](http://www.ncbi.nlm.nih.gov/gene/253430) | IPMK | inositol polyphosphate multikinase |
| [10261](http://www.ncbi.nlm.nih.gov/gene/10261) | IGSF6 | immunoglobulin superfamily, member 6 |
| [54518](http://www.ncbi.nlm.nih.gov/gene/54518) | APBB1IP | amyloid beta (A4) precursor protein-binding, family B, member 1 interacting protein |
| [8502](http://www.ncbi.nlm.nih.gov/gene/8502) | PKP4 | plakophilin 4 |
| [58473](http://www.ncbi.nlm.nih.gov/gene/58473) | PLEKHB1 | pleckstrin homology domain containing, family B (evectins) member 1 |
| [7100](http://www.ncbi.nlm.nih.gov/gene/7100) | TLR5 | toll-like receptor 5 |
| [348995](http://www.ncbi.nlm.nih.gov/gene/348995) | NUP43 | nucleoporin 43kDa |
| [5175](http://www.ncbi.nlm.nih.gov/gene/5175) | PECAM1 | platelet/endothelial cell adhesion molecule 1 |
| [121457](http://www.ncbi.nlm.nih.gov/gene/121457) | IKBIP | IKBKB interacting protein |
| [83759](http://www.ncbi.nlm.nih.gov/gene/83759) | RBM4B | RNA binding motif protein 4B |
| [170575](http://www.ncbi.nlm.nih.gov/gene/170575) | GIMAP1 | GTPase, IMAP family member 1 |
| [5150](http://www.ncbi.nlm.nih.gov/gene/5150) | PDE7A | phosphodiesterase 7A |
| [4144](http://www.ncbi.nlm.nih.gov/gene/4144) | MAT2A | methionine adenosyltransferase II, alpha |
| [11329](http://www.ncbi.nlm.nih.gov/gene/11329) | STK38 | serine/threonine kinase 38 |
| [8615](http://www.ncbi.nlm.nih.gov/gene/8615) | USO1 | USO1 vesicle docking protein homolog (yeast) |
| [55333](http://www.ncbi.nlm.nih.gov/gene/55333) | SYNJ2BP | synaptojanin 2 binding protein |
| [100130428](http://www.ncbi.nlm.nih.gov/gene/100130428) | LOC100130428 | IGYY565 |
| [57646](http://www.ncbi.nlm.nih.gov/gene/57646) | USP28 | ubiquitin specific peptidase 28 |
| [10773](http://www.ncbi.nlm.nih.gov/gene/10773) | ZBTB6 | zinc finger and BTB domain containing 6 |
| [3512](http://www.ncbi.nlm.nih.gov/gene/3512) | IGJ | immunoglobulin J polypeptide, linker protein for immunoglobulin alpha and mu polypeptides |
| [137994](http://www.ncbi.nlm.nih.gov/gene/137994) | LETM2 | leucine zipper-EF-hand containing transmembrane protein 2 |
| [6038](http://www.ncbi.nlm.nih.gov/gene/6038) | RNASE4 | ribonuclease, RNase A family, 4 |
| [1130](http://www.ncbi.nlm.nih.gov/gene/1130) | LYST | lysosomal trafficking regulator |
| [84924](http://www.ncbi.nlm.nih.gov/gene/84924) | ZNF566 | zinc finger protein 566 |
| [53346](http://www.ncbi.nlm.nih.gov/gene/53346) | TM6SF1 | transmembrane 6 superfamily member 1 |
| [7592](http://www.ncbi.nlm.nih.gov/gene/7592) | ZNF41 | zinc finger protein 41 |
| [79738](http://www.ncbi.nlm.nih.gov/gene/79738) | BBS10 | Bardet-Biedl syndrome 10 |
| [1378](http://www.ncbi.nlm.nih.gov/gene/1378) | CR1 | complement component (3b/4b) receptor 1 (Knops blood group) |
| [93474](http://www.ncbi.nlm.nih.gov/gene/93474) | ZNF670 | zinc finger protein 670 |
| [6404](http://www.ncbi.nlm.nih.gov/gene/6404) | SELPLG | selectin P ligand |
| [55251](http://www.ncbi.nlm.nih.gov/gene/55251) | PCMTD2 | protein-L-isoaspartate (D-aspartate) O-methyltransferase domain containing 2 |
| [5266](http://www.ncbi.nlm.nih.gov/gene/5266) | PI3 | peptidase inhibitor 3, skin-derived |
| [7764](http://www.ncbi.nlm.nih.gov/gene/7764) | ZNF217 | zinc finger protein 217 |
| [608](http://www.ncbi.nlm.nih.gov/gene/608) | TNFRSF17 | tumor necrosis factor receptor superfamily, member 17 |
| [4853](http://www.ncbi.nlm.nih.gov/gene/4853) | NOTCH2 | notch 2 |
| [3931](http://www.ncbi.nlm.nih.gov/gene/3931) | LCAT | lecithin-cholesterol acyltransferase |
| [9725](http://www.ncbi.nlm.nih.gov/gene/9725) | TMEM63A | transmembrane protein 63A |
| [90594](http://www.ncbi.nlm.nih.gov/gene/90594) | ZNF439 | zinc finger protein 439 |
| [28970](http://www.ncbi.nlm.nih.gov/gene/28970) | C11orf54 | chromosome 11 open reading frame 54 |
| [51520](http://www.ncbi.nlm.nih.gov/gene/51520) | LARS | leucyl-tRNA synthetase |
| [27180](http://www.ncbi.nlm.nih.gov/gene/27180) | SIGLEC9 | sialic acid binding Ig-like lectin 9 |
| [11024](http://www.ncbi.nlm.nih.gov/gene/11024) | LILRA1 | leukocyte immunoglobulin-like receptor, subfamily A (with TM domain), member 1 |
| [283588](http://www.ncbi.nlm.nih.gov/gene/283588) | LOC283588 | uncharacterized LOC283588 |
| [123169](http://www.ncbi.nlm.nih.gov/gene/123169) | LEO1 | Leo1, Paf1/RNA polymerase II complex component, homolog (S. cerevisiae) |
| [6693](http://www.ncbi.nlm.nih.gov/gene/6693) | SPN | sialophorin |
| [7263](http://www.ncbi.nlm.nih.gov/gene/7263) | TST | thiosulfate sulfurtransferase (rhodanese) |
| [8904](http://www.ncbi.nlm.nih.gov/gene/8904) | CPNE1 | copine I |
| [80017](http://www.ncbi.nlm.nih.gov/gene/80017) | C14orf159 | chromosome 14 open reading frame 159 |
| [84078](http://www.ncbi.nlm.nih.gov/gene/84078) | KBTBD7 | kelch repeat and BTB (POZ) domain containing 7 |
| [283237](http://www.ncbi.nlm.nih.gov/gene/283237) | TTC9C | tetratricopeptide repeat domain 9C |
| [65123](http://www.ncbi.nlm.nih.gov/gene/65123) | INTS3 | integrator complex subunit 3 |
| [100131827](http://www.ncbi.nlm.nih.gov/gene/100131827) | ZNF717 | zinc finger protein 717 |
| [54980](http://www.ncbi.nlm.nih.gov/gene/54980) | C2orf42 | chromosome 2 open reading frame 42 |
| [81793](http://www.ncbi.nlm.nih.gov/gene/81793) | TLR10 | toll-like receptor 10 |
| [79896](http://www.ncbi.nlm.nih.gov/gene/79896) | THNSL1 | threonine synthase-like 1 (S. cerevisiae) |
| [3577](http://www.ncbi.nlm.nih.gov/gene/3577) | CXCR1 | chemokine (C-X-C motif) receptor 1 |
| [23185](http://www.ncbi.nlm.nih.gov/gene/23185) | LARP4B | La ribonucleoprotein domain family, member 4B |
| [4609](http://www.ncbi.nlm.nih.gov/gene/4609) | MYC | v-myc myelocytomatosis viral oncogene homolog (avian) |
| [6039](http://www.ncbi.nlm.nih.gov/gene/6039) | RNASE6 | ribonuclease, RNase A family, k6 |
| [55070](http://www.ncbi.nlm.nih.gov/gene/55070) | DET1 | de-etiolated homolog 1 (Arabidopsis) |
| [84869](http://www.ncbi.nlm.nih.gov/gene/84869) | CBR4 | carbonyl reductase 4 |
| [55423](http://www.ncbi.nlm.nih.gov/gene/55423) | SIRPG | signal-regulatory protein gamma |
| [1781](http://www.ncbi.nlm.nih.gov/gene/1781) | DYNC1I2 | dynein, cytoplasmic 1, intermediate chain 2 |
| [3759](http://www.ncbi.nlm.nih.gov/gene/3759) | KCNJ2 | potassium inwardly-rectifying channel, subfamily J, member 2 |
| [23155](http://www.ncbi.nlm.nih.gov/gene/23155) | CLCC1 | chloride channel CLIC-like 1 |
| [10162](http://www.ncbi.nlm.nih.gov/gene/10162) | LPCAT3 | lysophosphatidylcholine acyltransferase 3 |
| [162394](http://www.ncbi.nlm.nih.gov/gene/162394) | SLFN5 | schlafen family member 5 |
| [55500](http://www.ncbi.nlm.nih.gov/gene/55500) | ETNK1 | ethanolamine kinase 1 |
| [30061](http://www.ncbi.nlm.nih.gov/gene/30061) | SLC40A1 | solute carrier family 40 (iron-regulated transporter), member 1 |
| [116496](http://www.ncbi.nlm.nih.gov/gene/116496) | FAM129A | family with sequence similarity 129, member A |
| [58477](http://www.ncbi.nlm.nih.gov/gene/58477) | SRPRB | signal recognition particle receptor, B subunit |
| [3655](http://www.ncbi.nlm.nih.gov/gene/3655) | ITGA6 | integrin, alpha 6 |
| [25880](http://www.ncbi.nlm.nih.gov/gene/25880) | TMEM186 | transmembrane protein 186 |
| [25853](http://www.ncbi.nlm.nih.gov/gene/25853) | DCAF12 | DDB1 and CUL4 associated factor 12 |
| [7514](http://www.ncbi.nlm.nih.gov/gene/7514) | XPO1 | exportin 1 (CRM1 homolog, yeast) |
| [219285](http://www.ncbi.nlm.nih.gov/gene/219285) | SAMD9L | sterile alpha motif domain containing 9-like |
| [788](http://www.ncbi.nlm.nih.gov/gene/788) | SLC25A20 | solute carrier family 25 (carnitine/acylcarnitine translocase), member 20 |
| [3772](http://www.ncbi.nlm.nih.gov/gene/3772) | KCNJ15 | potassium inwardly-rectifying channel, subfamily J, member 15 |
| [55017](http://www.ncbi.nlm.nih.gov/gene/55017) | C14orf119 | chromosome 14 open reading frame 119 |
| [1798](http://www.ncbi.nlm.nih.gov/gene/1798) | DPAGT1 | dolichyl-phosphate (UDP-N-acetylglucosamine) N-acetylglucosaminephosphotransferase 1 (GlcNAc-1-P transferase) |
| [3660](http://www.ncbi.nlm.nih.gov/gene/3660) | IRF2 | interferon regulatory factor 2 |
| [120425](http://www.ncbi.nlm.nih.gov/gene/120425) | AMICA1 | adhesion molecule, interacts with CXADR antigen 1 |
| [1545](http://www.ncbi.nlm.nih.gov/gene/1545) | CYP1B1 | cytochrome P450, family 1, subfamily B, polypeptide 1 |
| [55582](http://www.ncbi.nlm.nih.gov/gene/55582) | KIF27 | kinesin family member 27 |
| [28958](http://www.ncbi.nlm.nih.gov/gene/28958) | COA3 | cytochrome C oxidase assembly factor 3 |
| [120892](http://www.ncbi.nlm.nih.gov/gene/120892) | LRRK2 | leucine-rich repeat kinase 2 |
| [55303](http://www.ncbi.nlm.nih.gov/gene/55303) | GIMAP4 | GTPase, IMAP family member 4 |
| [84658](http://www.ncbi.nlm.nih.gov/gene/84658) | EMR3 | egf-like module containing, mucin-like, hormone receptor-like 3 |
| [5921](http://www.ncbi.nlm.nih.gov/gene/5921) | RASA1 | RAS p21 protein activator (GTPase activating protein) 1 |
| [10219](http://www.ncbi.nlm.nih.gov/gene/10219) | KLRG1 | killer cell lectin-like receptor subfamily G, member 1 |
| [84824](http://www.ncbi.nlm.nih.gov/gene/84824) | FCRLA | Fc receptor-like A |
| [158747](http://www.ncbi.nlm.nih.gov/gene/158747) | MOSPD2 | motile sperm domain containing 2 |
| [100505494](http://www.ncbi.nlm.nih.gov/gene/100505494) | ANKRD10-IT1 | ANKRD10 intronic transcript 1 (non-protein coding) |
| [722](http://www.ncbi.nlm.nih.gov/gene/722) | C4BPA | complement component 4 binding protein, alpha |
| [9750](http://www.ncbi.nlm.nih.gov/gene/9750) | FAM65B | family with sequence similarity 65, member B |
| [100862671](http://www.ncbi.nlm.nih.gov/gene/100862671) | LOC100862671 | uncharacterized LOC100862671 |
| [340527](http://www.ncbi.nlm.nih.gov/gene/340527) | NHSL2 | NHS-like 2 |
| [7099](http://www.ncbi.nlm.nih.gov/gene/7099) | TLR4 | toll-like receptor 4 |
| [6041](http://www.ncbi.nlm.nih.gov/gene/6041) | RNASEL | ribonuclease L (2',5'-oligoisoadenylate synthetase-dependent) |
| [10875](http://www.ncbi.nlm.nih.gov/gene/10875) | FGL2 | fibrinogen-like 2 |
| [79626](http://www.ncbi.nlm.nih.gov/gene/79626) | TNFAIP8L2 | tumor necrosis factor, alpha-induced protein 8-like 2 |
| [759](http://www.ncbi.nlm.nih.gov/gene/759) | CA1 | carbonic anhydrase I |
| [131450](http://www.ncbi.nlm.nih.gov/gene/131450) | CD200R1 | CD200 receptor 1 |
| [4967](http://www.ncbi.nlm.nih.gov/gene/4967) | OGDH | oxoglutarate (alpha-ketoglutarate) dehydrogenase (lipoamide) |
| [309](http://www.ncbi.nlm.nih.gov/gene/309) | ANXA6 | annexin A6 |
| [3493](http://www.ncbi.nlm.nih.gov/gene/3493) | IGHA1 | immunoglobulin heavy constant alpha 1 |
| [100529855](http://www.ncbi.nlm.nih.gov/gene/100529855) | ZNF625-ZNF20 | ZNF625-ZNF20 readthrough |
| [11118](http://www.ncbi.nlm.nih.gov/gene/11118) | BTN3A2 | butyrophilin, subfamily 3, member A2 |
| [154807](http://www.ncbi.nlm.nih.gov/gene/154807) | VKORC1L1 | vitamin K epoxide reductase complex, subunit 1-like 1 |
| [79689](http://www.ncbi.nlm.nih.gov/gene/79689) | STEAP4 | STEAP family member 4 |
| [10780](http://www.ncbi.nlm.nih.gov/gene/10780) | ZNF234 | zinc finger protein 234 |
| [57688](http://www.ncbi.nlm.nih.gov/gene/57688) | ZSWIM6 | zinc finger, SWIM-type containing 6 |
| [54097](http://www.ncbi.nlm.nih.gov/gene/54097) | FAM3B | family with sequence similarity 3, member B |
| [100129842](http://www.ncbi.nlm.nih.gov/gene/100129842) | ZNF737 | zinc finger protein 737 |
| [51311](http://www.ncbi.nlm.nih.gov/gene/51311) | TLR8 | toll-like receptor 8 |
| [84984](http://www.ncbi.nlm.nih.gov/gene/84984) | CEP19 | centrosomal protein 19kDa |
| [9581](http://www.ncbi.nlm.nih.gov/gene/9581) | PREPL | prolyl endopeptidase-like |
| [7096](http://www.ncbi.nlm.nih.gov/gene/7096) | TLR1 | toll-like receptor 1 |
| [151888](http://www.ncbi.nlm.nih.gov/gene/151888) | BTLA | B and T lymphocyte associated |
| [285636](http://www.ncbi.nlm.nih.gov/gene/285636) | C5orf51 | chromosome 5 open reading frame 51 |
| [3303](http://www.ncbi.nlm.nih.gov/gene/3303) | HSPA1A | heat shock 70kDa protein 1A |
| [7728](http://www.ncbi.nlm.nih.gov/gene/7728) | ZNF175 | zinc finger protein 175 |
| [26275](http://www.ncbi.nlm.nih.gov/gene/26275) | HIBCH | 3-hydroxyisobutyryl-CoA hydrolase |
| [114836](http://www.ncbi.nlm.nih.gov/gene/114836) | SLAMF6 | SLAM family member 6 |
| [3579](http://www.ncbi.nlm.nih.gov/gene/3579) | CXCR2 | chemokine (C-X-C motif) receptor 2 |
| [388325](http://www.ncbi.nlm.nih.gov/gene/388325) | SCIMP | SLP adaptor and CSK interacting membrane protein |
| [493](http://www.ncbi.nlm.nih.gov/gene/493) | ATP2B4 | ATPase, Ca++ transporting, plasma membrane 4 |
| [407008](http://www.ncbi.nlm.nih.gov/gene/407008) | MIR223 | microRNA 223 |
| [84287](http://www.ncbi.nlm.nih.gov/gene/84287) | ZDHHC16 | zinc finger, DHHC-type containing 16 |
| [27284](http://www.ncbi.nlm.nih.gov/gene/27284) | SULT1B1 | sulfotransferase family, cytosolic, 1B, member 1 |
| [28959](http://www.ncbi.nlm.nih.gov/gene/28959) | TMEM176B | transmembrane protein 176B |
| [440689](http://www.ncbi.nlm.nih.gov/gene/440689) | HIST2H2BF | histone cluster 2, H2bf |
| [150967](http://www.ncbi.nlm.nih.gov/gene/150967) | PKI55 | DKFZp434H1419 |
| [100507191](http://www.ncbi.nlm.nih.gov/gene/100507191) | LOC100507191 | uncharacterized LOC100507191 |
| [1524](http://www.ncbi.nlm.nih.gov/gene/1524) | CX3CR1 | chemokine (C-X3-C motif) receptor 1 |
| [84767](http://www.ncbi.nlm.nih.gov/gene/84767) | TRIM51 | tripartite motif-containing 51 |
| [79168](http://www.ncbi.nlm.nih.gov/gene/79168) | LILRA6 | leukocyte immunoglobulin-like receptor, subfamily A (with TM domain), member 6 |
| [6480](http://www.ncbi.nlm.nih.gov/gene/6480) | ST6GAL1 | ST6 beta-galactosamide alpha-2,6-sialyltranferase 1 |
| [84911](http://www.ncbi.nlm.nih.gov/gene/84911) | ZNF382 | zinc finger protein 382 |
| [51301](http://www.ncbi.nlm.nih.gov/gene/51301) | GCNT4 | glucosaminyl (N-acetyl) transferase 4, core 2 |
| [257144](http://www.ncbi.nlm.nih.gov/gene/257144) | GCSAM | germinal center-associated, signaling and motility |
| [100506144](http://www.ncbi.nlm.nih.gov/gene/100506144) | ZMYM6NB | ZMYM6 neighbor |
| [57535](http://www.ncbi.nlm.nih.gov/gene/57535) | KIAA1324 | KIAA1324 |
| [100507399](http://www.ncbi.nlm.nih.gov/gene/100507399) | HCG8 | HLA complex group 8 |
| [387751](http://www.ncbi.nlm.nih.gov/gene/387751) | GVINP1 | GTPase, very large interferon inducible pseudogene 1 |
| [284371](http://www.ncbi.nlm.nih.gov/gene/284371) | ZNF841 | zinc finger protein 841 |
| [81627](http://www.ncbi.nlm.nih.gov/gene/81627) | TRMT1L | tRNA methyltransferase 1 homolog (S. cerevisiae)-like |
| [79961](http://www.ncbi.nlm.nih.gov/gene/79961) | DENND2D | DENN/MADD domain containing 2D |
| [3494](http://www.ncbi.nlm.nih.gov/gene/3494) | IGHA2 | immunoglobulin heavy constant alpha 2 (A2m marker) |
| [55365](http://www.ncbi.nlm.nih.gov/gene/55365) | TMEM176A | transmembrane protein 176A |
| [284233](http://www.ncbi.nlm.nih.gov/gene/284233) | CYP4F35P | cytochrome P450, family 4, subfamily F, polypeptide 35, pseudogene |
| [492311](http://www.ncbi.nlm.nih.gov/gene/492311) | IGIP | IgA-inducing protein homolog (Bos taurus) |
| [54674](http://www.ncbi.nlm.nih.gov/gene/54674) | LRRN3 | leucine rich repeat neuronal 3 |
| [79971](http://www.ncbi.nlm.nih.gov/gene/79971) | WLS | wntless homolog (Drosophila) |
| [4064](http://www.ncbi.nlm.nih.gov/gene/4064) | CD180 | CD180 molecule |
| [677797](http://www.ncbi.nlm.nih.gov/gene/677797) | SNORA7B | small nucleolar RNA, H/ACA box 7B |
| [9019](http://www.ncbi.nlm.nih.gov/gene/9019) | MPZL1 | myelin protein zero-like 1 |
| [2358](http://www.ncbi.nlm.nih.gov/gene/2358) | FPR2 | formyl peptide receptor 2 |
| [677827](http://www.ncbi.nlm.nih.gov/gene/677827) | SNORA46 | small nucleolar RNA, H/ACA box 46 |
| [2352](http://www.ncbi.nlm.nih.gov/gene/2352) | FOLR3 | folate receptor 3 (gamma) |
| [9906](http://www.ncbi.nlm.nih.gov/gene/9906) | SLC35E2 | solute carrier family 35, member E2 |
| [80862](http://www.ncbi.nlm.nih.gov/gene/80862) | ZNRD1-AS1 | ZNRD1 antisense RNA 1 |
| [84622](http://www.ncbi.nlm.nih.gov/gene/84622) | ZNF594 | zinc finger protein 594 |
